# Cryo-EM structures of the CDK11-cyclin L-SAP30BP complex reveal mechanisms of CDK11 regulation

**DOI:** 10.1038/s41467-026-72329-4

**Published:** 2026-04-25

**Authors:** Amy J. S. McGeoch, Victoria I. Cushing, Theodoros I. Roumeliotis, Nora B. Cronin, Stephen J. Hearnshaw, Jyoti S. Choudhary, Claudio Alfieri, Basil J. Greber

**Affiliations:** 1 Division of Structural Biology, The Institute of Cancer Research, London, UK; 2 Functional Proteomics Group, The Institute of Cancer Research, London, UK; 3 London Consortium for High-Resolution Cryo-EM, The Francis Crick Institute, London, UK; 4 Present Address: The Francis Crick Institute, London, UK

**Keywords:** Cryoelectron microscopy, Kinases

## Abstract

The cyclin-dependent kinase CDK11 functions in transcription, mitotic progression, and mRNA splicing. Specifically, spliceosome activation during the B to B^act^ transition depends on phosphorylation of the U2 snRNP component SF3B1 by the CDK11-cyclin L-SAP30BP complex. Here, we present the structure of this spliceosome-activating CDK-cyclin complex, determined by cryogenic electron microscopy at 2.3 Å resolution. Our structure and biochemical experiments show that SAP30BP forms extensive interactions with cyclin L2, thereby stabilising it, and forms critical interactions with the C-terminal kinase lobe of CDK11 that promote complex assembly. Destabilisation of cyclin L2 in the absence of SAP30BP suggests that these principles are applicable to all CDK11-cyclin L complexes. Furthermore, we identify a pseudo-substrate sequence near the CDK11 C-terminus and provide evidence for a role of this segment in CDK11 auto-regulation. Finally, the structure of the CDK11-cyclin L-SAP30BP complex bound to the clinical high-affinity CDK11 inhibitor OTS964 and a comparison to OTS964-bound off-target complexes provide insight into the mechanism of OTS964 selectivity and specificity.

## Abstract (web summary)

McGeoch and co-authors use cryogenic electron microscopy and biochemistry to determine the structure of the CDK11-cyclin L-SAP30BP complex and elucidate the regulation of CDK11 by SAP30BP within the trimeric complex and by a CDK11 pseudo-substrate.

## Introduction

The cyclin-dependent protein kinase (CDK) family comprises 20 members in human cells, most of which form binary complexes with one or several members of the cyclin family^1^. CDKs play critical roles in cell growth and proliferation by controlling multiple key cellular pathways. The canonical role of CDK-cyclin complexes is in cell cycle control, where the enzymatic activities of CDKs oscillate due to a complex interplay of regulatory mechanisms, including cyclin availability^2^. Additional CDKs that are usually constitutively associated with their partner cyclins perform prominent roles in transcription control and mRNA splicing^1^. CDK11 is a representative of this latter group.

CDK11 is expressed in several variants and isoforms in human cells. Human CDK11 is encoded by two genes, and the resulting full-length proteins, CDK11A^p110^ and CDK11B^p110^, are 97% identical on the sequence level^1^. Further CDK11 diversity originates from alternatively spliced isoforms^3^, and from the expression of a shorter CDK11 variant called CDK11^p58^, which arises from translation initiation at a cell-cycle-regulated internal ribosomal entry site^4^. Therefore, the N-terminus of the resulting protein is truncated compared to the full-length CDK11^p110^ isoform (Supplementary Fig. 1a)^3–5^. The CDK11^p58^ isoform is expressed in a cell-cycle specific manner and is required for progression through mitosis^6,7^ while CDK11^p110^ is constitutively expressed and is involved in transcription and splicing^8–14^. Both CDK11^p110^ and CDK11^p58^ form stable complexes with cyclins L1 and L2, which are encoded by distinct genes^15–17^.

Early work on the involvement of CDK11 in splicing documented its association with splicing factors, such as splicing regulator proteins^8,15,17^. However, a mechanistic understanding of the role of CDK11 in the core splicing pathway remained elusive until the recent discovery that CDK11 inhibition impairs phosphorylation of the protein SF3B1, a key component of the SF3B complex within the U2 small nuclear ribonucleoprotein (U2 snRNP). The resulting deficiency in SF3B1 phosphorylation prevents spliceosomal activation, thereby stalling the spliceosome between the B- and B^act^-complex stages^18^. This causes widespread intron retention^18,19^, revealing CDK11 as a key player in spliceosome activation. This critical activity of CDK11 requires the formation of a trimeric spliceosome-activating CDK-cyclin complex that is composed of CDK11^p110^, cyclin L1/2 and SAP30BP^19^. SAP30BP is a transcription and splicing factor that has been implicated in the splicing of short introns^20,21^. In the context of the CDK11-bound complex, SAP30BP stabilizes the CDK11-cyclin L complex, contributes to CDK11 activation, and mediates interactions with spliceosomal components^19^.

Due to their important roles in cell growth and proliferation, CDKs have been the target of intense drug discovery efforts, with a focus on CDKs 4, 6, 7 and 9^22,23^. In addition to these intensively studied CDKs, CDK11 is an emerging drug target^14,24,25^ because its activity is required for the growth of multiple cancer cell lines^13,14,26,27^. Even though dedicated inhibitors for CDK11 have been discovered^14,28^, one of the clinically most advanced CDK11 inhibitors, OTS964, was originally mischaracterised as a PDZ-binding kinase (PBK)/Lymphokine-activated killer T-cell originated protein kinase inhibitor^29^ and only later discovered to potently inhibit CDK11^30^. This inhibitor is currently in clinical evaluation for its activity against CDK11, and it has been instrumental in characterising the role of CDK11 in splicing^18^.

Despite the critical importance of CDK11 and its cyclin L and SAP30BP partners for our understanding of fundamental biological processes and for cancer drug discovery, structures of full trimeric CDK11-cyclin L-SAP30BP complexes have remained elusive. This hampers our understanding of splicing activation and CDK11 regulation. Here, we report the structure of the fully assembled CDK11B-cyclin L2-SAP30BP trimer in complex with AMP-PNP or the inhibitor OTS964, along with structures of OTS964 bound to the off-targets CDK2-cyclin A2 and CDK7-cyclin H-MAT1 (the CDK-activating kinase, or CAK). Based on these results, we test structure-derived hypotheses on complex assembly and CDK11 regulation by biochemical experiments. Our work provides insight into the architecture and assembly of the activated CDK11 complex, reveals an auto-regulatory pseudo-substrate segment near the CDK11 C-terminus, and contributes to our understanding of the molecular basis of OTS964 selectivity.

## Results

### Structure determination

To investigate the mechanism by which SAP30BP promotes CDK11-cyclin L complex formation and CDK11 activation, we recombinantly expressed trimeric CDK11B-cyclin L2-SAP30BP in insect cells and purified the complex by affinity and gel filtration chromatography (Supplementary Fig. 1b–d). To facilitate purification of homogeneous protein complexes and cryo-EM grid preparation, we truncated the N-terminus of CDK11, generating CDK11^p58^ (CDK11B^p110^ residues 357–795) and removed the C-terminal tail of cyclin L2, including its predicted disordered RS-domain, resulting in expression of cyclin L2 residues 1–319 (Supplementary Fig. 1a, b). Attempts to prepare CDK11-cyclin L2 complexes or express cyclin L2 on its own failed (Supplementary Fig. 1e). This is consistent with previous reports indicating instability of cyclin L in the absence of SAP30BP, both when recombinantly expressed^31^ and in the native context in human cells^19^. These findings indicate that SAP30BP is an obligate binding partner of cyclin L and suggest that SAP30BP is likely to be a component of both CDK11^p110^ and CDK11^p58^-containing complexes.

**Fig. 1:**
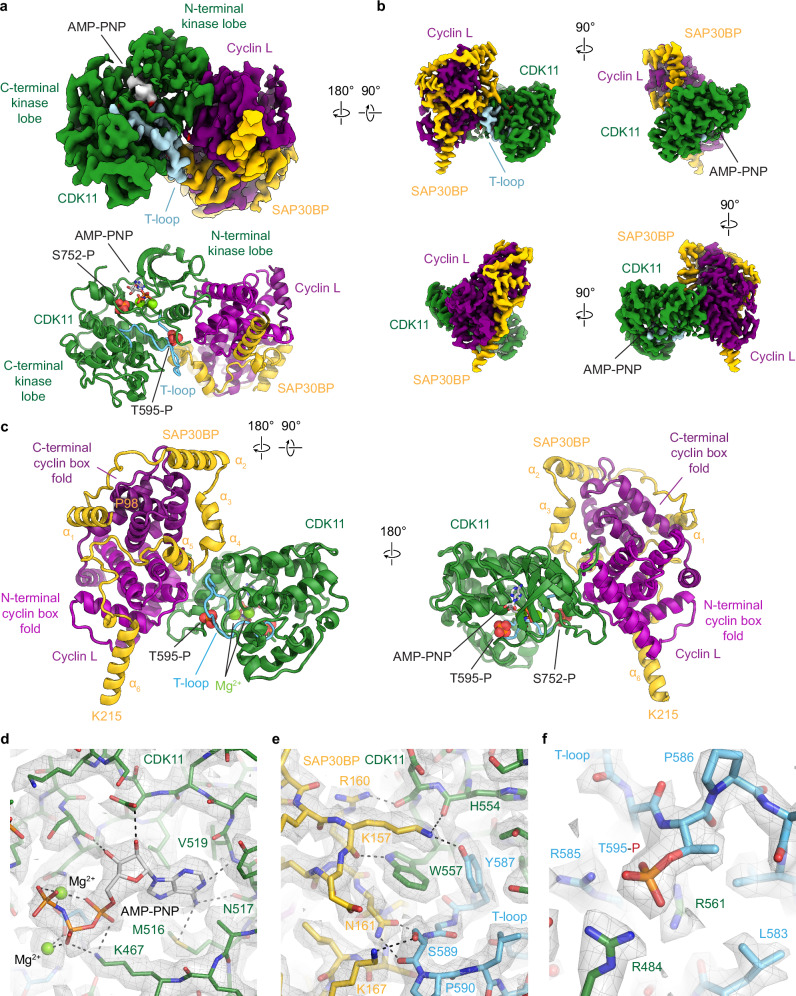
Cryo-EM structure of the CDK11-cyclin L-SAP30BP complex. **a** Cryo-EM map (top) and atomic model (bottom) of the CDK11-cyclin L-SAP30BP complex with subunits distinctly coloured (CDK11 green, T-loop blue, cyclin L purple, SAP30BP yellow and AMP-PNP white). The complex is shown in the canonical kinase view with the N-terminal kinase lobe at the top and the C-terminal kinase lobe at the bottom, and visualising the active site in between the two kinase lobes. T595-P and S752-P denote phosphorylated residues. **b** Cryo-EM map of the CDK11-cyclin L-SAP30BP complex in four orientations (related by 90° rotations). The rotations relating the view shown in (**a**) and the first view in (**b**) are indicated. **c** Detailed view of the atomic model derived from the cryo-EM map. The subunits are coloured as in (**a**, **b**); additionally, the phosphorylated residues T595 in the T-loop and S752, as well as the N- and C-terminal ends of the modelled portion of SAP30BP, are indicated. The first views shown in (**b**, **c**) are the same. **d**, **e** Sections of the cryo-EM map of the CDK11-cyclin L-SAP30BP complex. Density is shown in semi-transparent grey surface and mesh. CDK11 is shown in green (T-loop cyan) and SAP30BP in yellow. **f** Molecular environment of phosphorylated CDK11 T595 within the T-loop (cyan) with surrounding arginine residues.

Using this reconstituted complex, we obtained a 2.3 Å-resolution cryo-EM reconstruction of the core module of the CDK11B-cyclin L2-SAP30BP complex, visualising approx. 76 kDa in molecular weight (Fig. 1a, b and Supplementary Fig. 2a–d). The excellent quality of our cryo-EM map enabled building and refinement of a full atomic model (CDK11B^p110^ residues 427–740 and 751–762; cyclin L2 residues 67–302; SAP30BP residues 98–215) and a detailed analysis of molecular contacts (Fig. 1a, c–f). It reveals the molecular architecture of the CDK11B-cyclin L2-SAP30BP complex, its interactions with the nucleotide analogue AMP-PNP (Fig. 1d), and multiple phosphorylated residues on CDK11 (Fig. 1a, c, f).

Structural modelling using AlphaFold3^32^ suggests that the highly charged N-terminal extension of CDK11^p110^, much of which consists of low-complexity regions (Supplementary Fig. 1a), is disordered in solution and does not contribute to folding and assembly of the core region of the CDK11-cyclin L-SAP30BP complex (Supplementary Fig. 3). Therefore, the parts of CDK11^p110^ that are absent in our cryo-EM specimen are likely disordered in the free trimeric complex, suggesting that our structural analysis is valid for both CDK11^p58^ and CDK11^p110^ in the context of CDK11-cyclin L-SAP30BP complexes. Similarly, the sequence identity between cyclins L1 and L2 within the region observed in our structure is high (82% for residues 67–302), and the general conclusions are valid for both cyclin L variants. We will therefore use the designations CDK11 and cyclin L unless we refer to a certain variant of these proteins specifically.

### Architecture of the CDK11-cyclin L-SAP30BP complex and mechanism of its stabilisation by SAP30BP

**Fig. 2:**
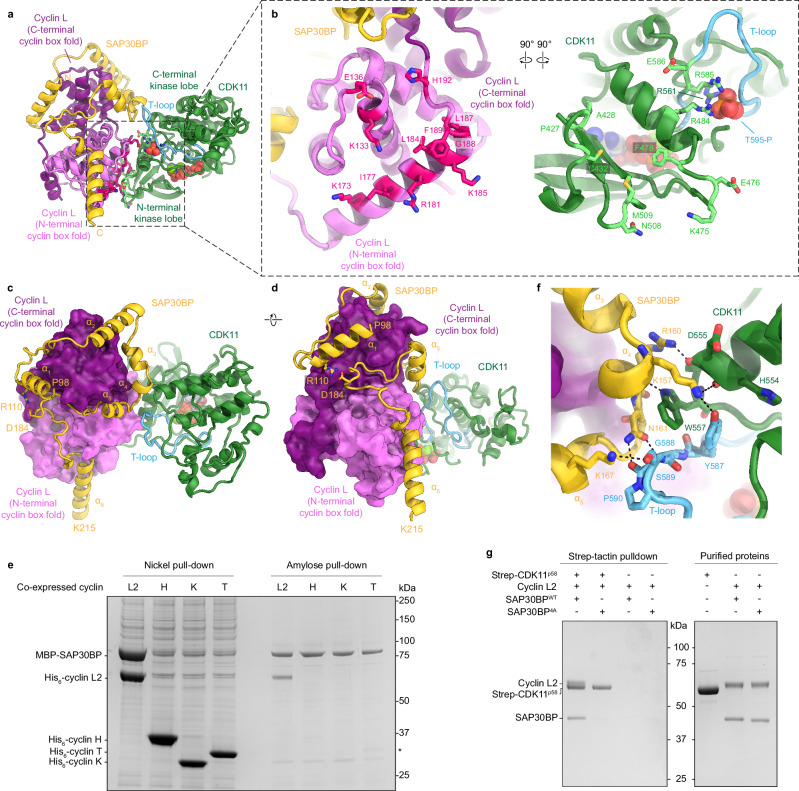
Molecular basis of CDK11-cyclin L-SAP30BP complex assembly. **a**, **b** Interactions between CDK11 and cyclin L. CDK11 is coloured green, with residues forming the interface with cyclin L coloured light green (distance cut-off: 3.6 Å) and the T-loop in blue; cyclin L is coloured pink and purple (N- and C-terminal cyclin box folds, respectively), with residues forming the interface with CDK11 coloured bright red. SAP30BP is coloured yellow, and the N- and C-terminal ends of the model are labelled. **c**, **d** Interactions of SAP30BP with cyclin L (colours as in **a**–**c**). **e** Small-scale co-purification assay from insect cell lysates co-expressing MBP-SAP30BP with His_6_-tagged cyclins L2, H, K and T. One representative result from two biological replicates is shown. Only His_6_-cyclin L2 co-purifies MBP-SAP30BP (nickel-sepharose elution), and MBP-SAP30BP co-purifies His_6_-cyclin L2 (amylose elution; a weak band in the cyclin T elution denoted by an asterisk might correspond to a weakly bound or transiently forming complex). **f** Interactions of SAP30BP with CDK11 (colours as in **a**). Hydrogen bonds are indicated as black dashed lines. **g** In vitro pulldown of purified cyclin L2 in complex with SAP30BP wild type (WT) and 4A mutant. One representative experiment from two independently conducted technical replicates is shown. Immobilised Strep-CDK11^p58^ retains cyclin L2-SAP30BP^WT^ (first lane) but not cyclin L2-SAP30BP^4A^ (second lane). The third and fourth lanes are negative controls without pre-incubation of the resin with Strep-CDK11^p58^. The right-hand panel shows the purified proteins used as input for the pull-down. Source data are provided in a Source data file.

Our structure shows that CDK11 and cyclin L form a canonical CDK-cyclin pair in which direct interactions are primarily mediated by the N-terminal kinase lobe of CDK11 and the N-terminal cyclin box fold of cyclin L (Fig. 2a, b). The T-loop of CDK11 is found in an extended, active conformation, and the features of our cryo-EM map indicate that it is phosphorylated at T595 (CDK11B^p110^ residue numbering; Fig. 1a, c, f). The presence of post-translational modifications of the T-loop is in line with preparations of other CDKs from insect cells^33–35^. The phosphate moiety is coordinated by three arginine residues (R484, R585 and R561), which thereby stabilise the active T-loop conformation, a feature that is commonly found in CDKs (Figs. 1f and 2b).

Our cryo-EM map visualises a SAP30BP segment comprising residues from P98 to K215, which predominantly interacts with cyclin L (Fig. 2c, d). The C-terminal U2AF ligand motif (ULM) of SAP30BP, which engages in an interaction with the U2AF homology motif (UHM) of the splicing factor RBM17 to chaperone RBM17 to phosphorylated SF3B1 (ref. ^21^), is not visualised in our cryo-EM map. Therefore, this binding site for RBM17 on SAP30BP likely remains solvent-exposed and accessible to other proteins in the CDK-cyclin-bound complex. This may allow targeting of the entire trimeric complex to spliceosomal sites of action. Similarly, SAP30BP residues 73–88 encoded by its alternatively spliced exon 3 lie outside the region interacting with CDK11 and cyclin L, making this region available to form isoform-dependent interactions with mRNA biogenesis and processing factors, thereby regulating transcription and the alternative splicing of short introns^20^.

SAP30BP residues 98–184 form five α-helical segments connected by extended linkers that encircle the C-terminal cyclin box fold of cyclin L. This ring around cyclin L is sealed by a salt bridge between SAP30BP residues R110 and D184 (Fig. 2a–d). Subsequent residues of SAP30BP interact with the N-terminal cyclin box fold of cyclin L, terminating in a long, sixth, α-helix that extends into the solvent and likely continues beyond the last residue (K215) modelled in our structure (Figs. 1c and 2c, d). In addition to the interfaces contributed by the α_1_–α_6_ helices, the extended SAP30BP segments forming the α_1_–α_2_ and α_5_–α_6_ connections interact with cyclin L (Supplementary Fig. 4a–f). We note that neither of these segments occupy the hydrophobic groove that is bound by docking sequences known as RxL or Cy motifs in many cyclins^36–38^.

These extensive protein–protein interactions of SAP30BP with both cyclin box folds bury approx. 3530 Å^2^ in surface area^39^, rationalising why cyclin L stability is compromised in the absence of SAP30BP (Supplementary Fig. 1e and refs. ^19,31^). Tight binding and formation of a stable complex between cyclin L and SAP30BP are in agreement with previous biochemical analysis^19^. These interactions appear to be highly specific to cyclin L, as SAP30BP did not form stable complexes when co-expressed with the phylogenetically related cyclins H, K and T1 (Fig. 2e)^40^. Mapping of the SAP30BP-cyclin L interactions onto a multiple sequence alignment shows that many interactions occur with non-conserved cyclin residues (Supplementary Fig. 5). This indicates that sequence divergence between cyclin L and cyclins H, T and K is the likely reason for SAP30BP specificity.

In contrast to the extensive interactions between cyclin L and SAP30BP, the molecular interface between SAP30BP and CDK11 is rather modest in extent; it is limited to an 11-residue segment of SAP30BP (residues 157–167) that contacts the C-terminal kinase lobe of CDK11 and residues Y587 and S589 in the CKD11 T-loop (Figs. 1e and 2f). These interactions bury approximately 360 Å^2^ of surface area^39^. They comprise multiple side chain-side chain and side chain-main chain hydrogen bonds, and a cation–π interaction between CDK11 W557 and SAP30BP K157. Engineered mutations in the SAP30BP residues responsible for forming these interactions (K157A, R160A, N161A, K167A; SAP30BP^4A^) reduce the stability of the interaction of cyclin L-SAP30BP complexes with CDK11 (Fig. 2g). The contacts between SAP30BP and the CDK11 T-loop may be subject to modulation by post-translational modifications. Experiments using *Drosophila* CDK11 indicate that phosphorylation of S589 (human numbering; S712 in *Drosophila*) may play a regulatory role by suppressing CDK11 activity^41^. In our cryo-EM map, this residue is un-phosphorylated and forms a charged hydrogen bond with SAP30BP K167 (Figs. 1e and 2f).

Our structure thus provides a structural view of how SAP30BP promotes both cyclin L stability and CDK11-cyclin L complex formation. These functions of SAP30BP have been shown to critically contribute to CDK11 activity in splicing^19^ and are likely to also support the other cellular functions of CDK11.

### CDK11 contains an auto-regulatory pseudo-substrate segment that can occlude the substrate binding site

**Fig. 3:**
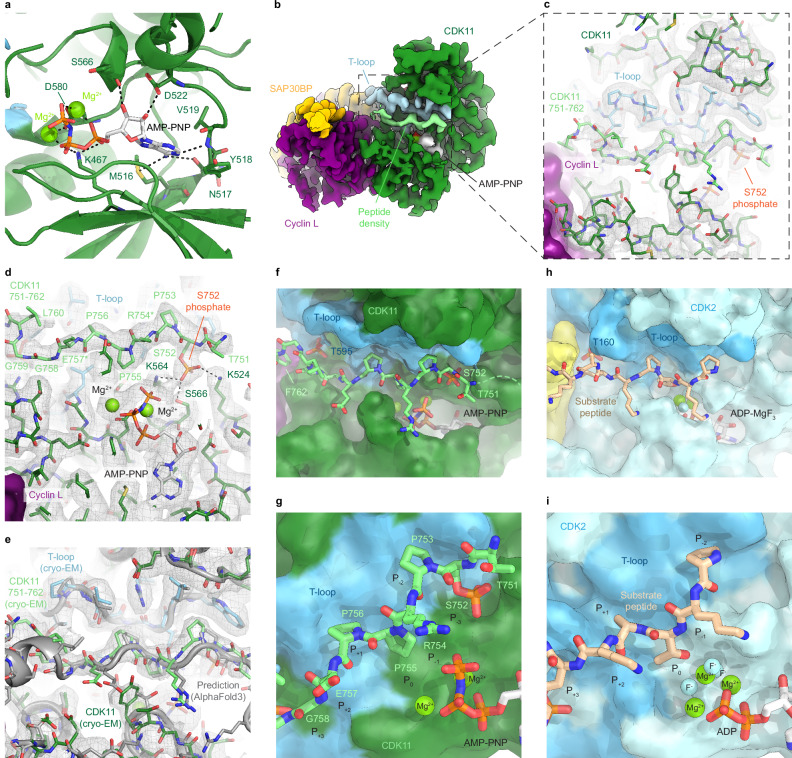
Structural analysis of a pseudo-substrate visualised near the CDK11 active site. **a** Bound AMP-PNP (white) in the active site of CDK11 (green). Residues involved in interactions with the nucleotide are shown as sticks, hydrogen bonds are indicated as black dashed lines, and magnesium ions are shown as green spheres. **b** The pseudo-substrate density occupying the substrate binding site of CDK11 is shown in light green (CDK11 green, T-loop blue, SAP30BP yellow and cyclin L purple). **c**, **d** Cryo-EM map shown as grey mesh and semi-transparent surface, with fitted model coloured as in (**b**). **e** Superposition of the AlphaFold 3 model (grey) onto the refined atomic model (shown in colours as in **b**), shown with the cryo-EM map. **f**, **g** Molecular model of the CDK11 pseudo-substrate segment (residues 751–762, light green) occluding the substrate binding site. **f** Is shown in the same view as (**c**). The N-terminal kinase lobe is omitted in (**g**) to reveal accommodation of the pseudo-substrate at its binding site in the C-terminal kinase lobe. Positions of pseudo-substrate residues relative to the phospho-acceptor position (P_0_, occupied by a proline in the pseudo-substrate) are indicated. **h**, **i** CDK2-cyclin A (cyan and yellow) in complex with a substrate peptide (sand) in the same views as **f**, **g** (PDB ID 3QHR)^42^.

The CDK11 active site in our cryo-EM structure is found in a state previously observed in complexes primed for phosphoryl transfer^42^, in which the phosphates of the bound AMP-PNP molecule are coordinated to two magnesium ions and the catalytic lysine (K467 in CDK11^p110^) (Fig. 3a). The base of the AMP-PNP molecule is bound to backbone atoms of N517 and V519 in the hinge region of CDK11 (Figs. 1d and 3a), as is typically observed in kinase structures^43^, and its exocyclic amino group is within hydrogen bonding distance of the sulphur atom of the gatekeeper residue M516 (Fig. 3a).

The cryo-EM map in this region is generally of excellent quality (Fig. 1d), allowing unambiguous assignment of side-chain positions and molecular contacts. One exception to this is the G-rich loop of CDK11 (residues 445–450, sequence EEGTYG), which exhibits weak density, indicating flexibility. A second element of weak density is positioned just beyond the G-rich loop and the γ-phosphate of the AMP-PNP molecule. While fragmented in fully sharpened cryo-EM maps, this segment becomes continuous in more modestly sharpened maps (sharpening *b*-factor = −10 Å^2^) and indicates the presence of an extended peptide occupying the substrate binding site of the kinase (Fig. 3b, c). Features of the observed density agree with the identity of CDK11 residues 751–762 (TSPRPPEGGLGF; Fig. 3c, d). This sequence is predicted to occupy this site by AlphaFold3^32^ (Fig. 3e). Strikingly, our cryo-EM map and a mass spectrometry analysis of our purified complexes both indicate that S752 is partially phosphorylated in our sample (Fig. 3c, d, Supplementary Table 1 and Supplementary Data 1). The phosphate is accommodated in a pocket near the CDK11 active site, forming interactions with the side chains of CDK11 K524, K564 and S566 (Fig. 3c, d). This pocket is distinct from the active site pocket itself, and the phosphorylated S752 does not form direct interactions with the bound AMP-PNP nucleotide, the catalytic aspartate (CDK11^p110^ D580), or the bound magnesium ions (Fig. 3f, g). Prior biochemical analysis has shown that the enzyme responsible for phosphorylation of S752 (S737 in isoform 1 studied in ref. ^44^) is checkpoint kinase 2 (CHK2), and that this modification enhances splicing in a cell-based assay^44^.

The positioning of this CDK11 segment (residues 751–762) coincides with substrate peptides visualised in other CDK structures^36,42^ and thus precludes substrate access to the active site (Fig. 3f–i). The position of the phosphorylatable residue in actual substrates (denoted P_0_) is occupied by P755 in the active site-occluding peptide in CDK11, precluding catalysis from occurring. The neighbouring P_+1_, P_−1_, P_−2_ and P_−3_ positions are occupied by P756, R754, P753 and the phosphorylatable S752 (Fig. 3f, g). Proline in the P_+1_ position satisfies the canonical sequence preference of CDKs, which are proline-directed^36,45–47^. Indeed, the configuration of the CDK11 active site-occluding peptide with prolines in the P_+1_ and P_−2_ positions and a positively charged residue in the P_−1_ position closely resembles the structure of a CDK2 complex bound to a synthetic peptide (Fig. 3f–i)^42^. This indicates that the CDK11 segment occupying its own substrate-binding site contains residues mimicking the properties of true CDK substrates in several positions in the immediate vicinity of the P_0_ acceptor site. In positions P_+4_ and P_+6_ at the C-terminal side of the P_0_ position, two glycine residues (G759 and G761) are positioned such that their amide protons can form interactions with the phosphorylated side chain of T595 within the CDK11 T-loop (Supplementary Fig. 6a, b). These interactions appear to replace the salt bridge observed between the T-loop phosphate and the positively charged P_+3_ residue in substrates bearing the full (S/T)-P-X-(R/K) consensus sequence (Supplementary Fig. 6c)^36,42^.

Given that the density for this segment is weaker than for the core fold of CDK11, this structural element is likely dynamic. Overall, these observations indicate that the C-terminal region of CDK11 harbours a pseudo-substrate. This pseudo-substrate might be involved in the modulation of substrate access to the active site, which might suggest a possible auto-regulatory role, in line with the documented roles of pseudo-substrates in the regulation of kinases^48^.

**Fig. 4:**
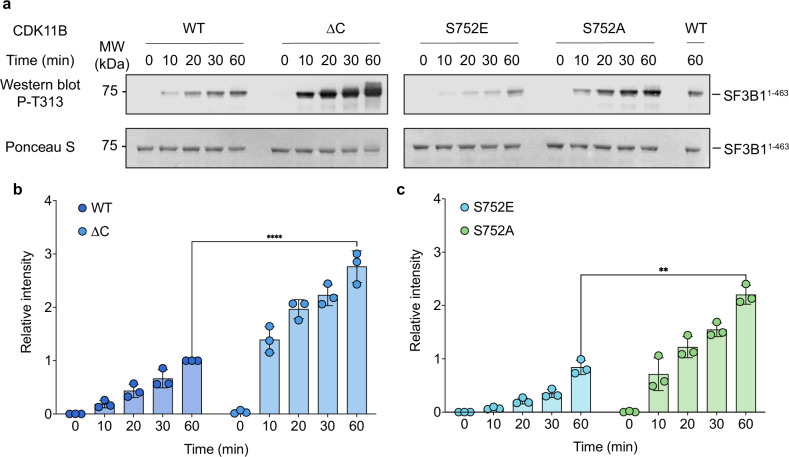
Biochemical characterisation of the CDK11 pseudo-substrate. **a** Kinase assay using an SF3B1^1–463^ substrate and trimeric kinase complexes containing CDK11 wild type (WT) and ∆C, S752E and S752A mutants. SF3B1 phospho-T313 (P-T313) was detected using Western blot. A loading control stained with Ponceau S is provided. The assay was performed as *N* = 3 biological replicates. One representative experiment is shown here; the remaining two replicates used for quantification are shown in Supplementary Fig. 6f, g. **b**, **c** Quantification of Western blot band intensities, presented as the mean ± standard deviation and individual data points of *N* = 3 biological replicates of the kinase assay. The significance level for activity comparisons between complexes, determined by 2-way ANOVA and Sidak’s multiple comparisons test, is provided (**: *p* < 0.01 (*p* = 0.0032); ****: *p* < 0.0001). Source data are provided in a Source data file.

To test the effect of this putative pseudo-substrate on CDK11 activity, we performed kinase assays using wild-type CDK11^p58^-cyclin L2-SAP30BP and complexes harbouring a C-terminal deletion in CDK11, removing the putative pseudo-substrate segment (CDK11 ∆C), the phospho-mimetic CDK11 S752E mutation, and the non-phosphorylatable CDK11 S752A mutation (CDK11B^p110^ residue numbering is used for consistency; Supplementary Fig. 6d). The CDK11 ∆C truncation, eliminating the pseudo-substrate, increases CDK11 activity against the spliceosomal SF3B1^1–463^ substrate to above wild-type level (Fig. 4a, b and Supplementary Fig. 6e–g). This is in line with an inhibitory effect of the active site-occluding conformation of the CDK11 pseudo-substrate observed in our structure. Furthermore, CDK11 carrying the non-phosphorylatable S752A mutation within the pseudo-substrate is more active than CDK11 harbouring the phospho-mimetic S752E mutation (Fig. 4a, c and Supplementary Fig. 6e–g). This is consistent with a possible role of a negative charge on S752 in promoting pseudo-substrate accommodation, suggesting a possible modulatory role for S752 phosphorylation. We note that substantial kinase activity is observed in all CDK11 variants tested. This indicates that none of the tested configurations of S752 results in complete inhibition, probably because true substrates can outcompete all configurations of the pseudo-substrate, albeit with variable efficiency.

While our data establish the presence of an active site-occluding segment in CDK11 that resembles the pseudo-substrates documented in other systems^48,49^, a comprehensive mechanistic understanding of the ways in which this segment impacts CDK11 activity or specificity towards its different substrates will require further study (see “Discussion”). We note that, unlike for CDK11, we did not observe reliably predicted pseudo-substrates in models of the remaining human CDKs produced using AlphaFold 3 (Supplementary Fig. 7 and Supplementary Data 2). Therefore, the regulation of CDK11 by a pseudo-substrate might be unique among CDKs.

### Cyclins are versatile docking platforms for non-enzymatic CDK modulators

**Fig. 5:**
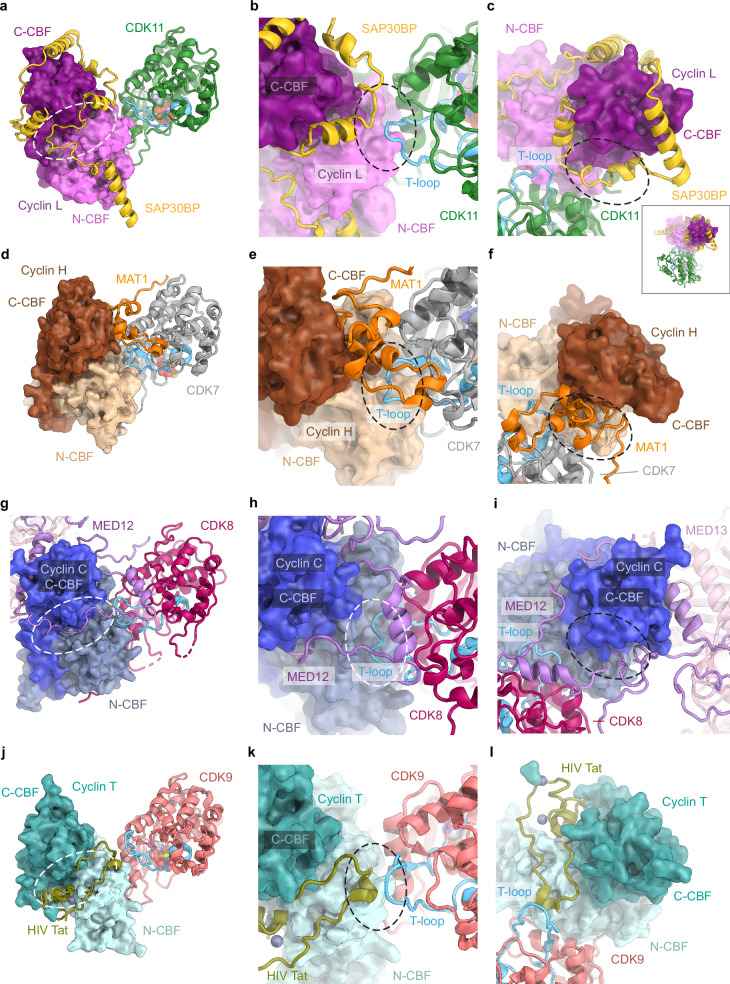
Comparison of CDK-cyclin complexes bound by accessory factors that aid CDK assembly and activity. **a**–**c** CDK11-cyclin L-SAP30BP, coloured as in Fig. 2. Throughout the figure, protein subunits are labelled, and areas of interest are denoted by dashed outlines. T-loops are shown in cyan, and N- and C-terminal cyclin box folds (abbreviated N-CBF and C-CBF) are coloured in distinct shades. An inset shows the complex in the same view as in (**c**), but zoomed out for orientation. **d**–**f** The human CAK (CDK7-cyclin H-MAT1). CDK7 is shown in grey, MAT1 in orange and cyclin H in brown. **g**–**i** The CKM module of mediator (CDK8-cyclin C-MED12-MED13). CDK8 is shown in red, MED12 in violet, and cyclin C in blue. **j**–**l** The HIV protein Tat hijacking the pTEF-B complex (CDK9-cyclin T). CDK9 is shown in salmon, cyclin T in teal, and Tat in olive green. For visualisation, the complexes were superposed on the CDK subunit, and each column in the figure shows all complexes from the same viewing direction.

Stably interacting protein factors serve to regulate the assembly and activity of several CDK-cyclin complexes. In addition to the CDK11-cyclin L-SAP30BP complex described here (Fig. 5a–c), these include the metazoan CAK (CDK7-cyclin H-MAT1, Fig. 5d–f)^33^, the CKM module of mediator (CDK8-cyclin C-MED12-MED13, Fig. 5g–i)^50^, and pTEF-B (CDK9-cyclin T, Fig. 5j–l), which is captured and effectively hijacked by retroviral proteins, such as HIV Tat^51^. We performed a structural comparison to determine if shared mechanistic principles or interaction motifs underlie the apparent functional similarities between these accessory factors. Because SAP30BP is primarily a cyclin-binding protein, we did not include exclusively CDK-binding proteins, such as Cks1/2 (e.g. ref. ^52^) in this analysis.

Our structure of the CDK11-cyclin L-SAP30BP complex reveals that SAP30BP in the CDK11-containing complex, MAT1 in the CDK7-containing CAK complex (PDB ID 6XBZ)^33^, and MED12 in the CDK8-containing CKM complex (PDB ID 7KPV)^50^ do not share meaningful structural resemblance even though their functions—complex assembly and enhancing CDK activity—are similar (Fig. 5a, d, g). However, there are some shared interaction regions on their respective CDK and cyclin partners used by SAP30BP, MAT1 and MED12. All three accessory subunits contact the T-loop of their partner CDK (Fig. 5b, e, h) and the region around the first and third conserved helices of the C-terminal cyclin box fold of their partner cyclins (Fig. 5c, f, i), albeit in structurally distinct ways. Furthermore, both SAP30BP and MED12 contact the region near the interface between the N- and C-terminal cyclin box folds of their partner cyclins (Fig. 5a, g), though again without appreciable similarity in the sequence elements used to form these interactions.

The N-terminal half of the HIV Tat protein also exploits the latter interface region when binding to cyclin T within P-TEFb (PDB ID 3MIA)^51^, thereby activating CDK9, changing its substrate specificity, and recruiting it to a sequence element within the HIV mRNA to promote transcription^51^. However, on the molecular level, the details of the interaction again differ from the examples discussed above (Fig. 5j–l). These considerations suggest that CDK-cyclin-binding proteins use a combination of a few shared and additional unique interaction surfaces on their targets, but that the molecular details of these interactions and the CDK-activating interactions employed differ in each case. Our comparison additionally shows that the extent of the interaction interface formed by SAP30BP on cyclin L is uniquely large (3530 Å^2^), compared to the buried surface area in the other complexes (1200, 2640 and 1420 Å^2^ for cyclin H-MAT1, MED12-cyclin C and Tat-cyclin T, respectively)^39^. Conversely, the interaction interface between SAP30BP and CDK11 (360 Å^2^) is substantially smaller than for MAT1 and CDK7 (1220 Å^2^)^33^. These differences likely reflect the unique function of SAP30BP in stabilisation of cyclin L, whereas MAT1 primarily promotes CDK7-cyclin H complex assembly and activation^33,53,54^.

### Structure of CDK11-cyclin L-SAP30BP-OTS964

OTS964 is a non-covalent, ATP-competitive inhibitor that binds to the active site of its target kinases (Supplementary Fig. 8a). While the structure of the isolated kinase domain of CDK11B in complex with OTS964 has been determined previously (PDB ID 7UKZ)^31^, results reported in that study indicate that cyclin binding improves OTS964 affinity for CDKs and suggest that the isolated kinase domain might not be able to fully recapitulate the compound-binding properties of the full-length cellular CDK11 protein^31^. This is in agreement with the general idea that the inhibitor-binding properties of CDKs depend on the state of the regulatory elements of the kinase domain, which are controlled by cyclin binding and T-loop phosphorylation^55^. We therefore performed isothermal titration calorimetry (ITC) to determine the affinity of OTS964 to CDK11B^p58^-cyclin L2-SAP30BP and the isolated CDK11B^p58^ monomer. These measurements indicate that the trimeric CDK11-cyclin L-SAP30BP complex binds OTS964 with slightly higher affinity than the isolated CDK11 monomer does (*K*_D, complex_ = 4.3 nM and *K*_D, monomer_ = 9.8 nM; Supplementary Fig. 8b–f). Despite the small magnitude of this affinity difference, we decided to investigate the structural basis of the high affinity of OTS964 for CDK11 and its mechanism of selectivity in the context of the activating binding partners of the kinase. To this end, we determined the structure of CDK11-cyclin L-SAP30BP-OTS964 at 2.4 Å resolution (Supplementary Fig. 9a–f).

**Fig. 6:**
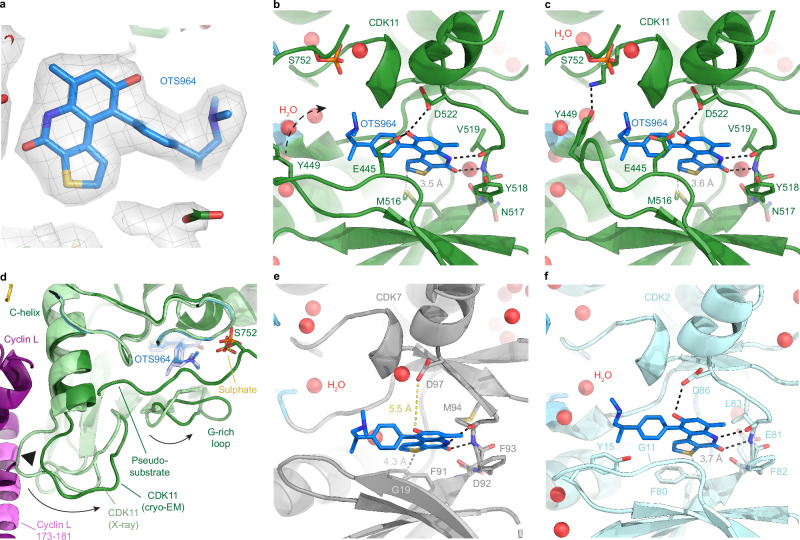
Structure of OTS964 bound to CDK11-cyclin L-SAP30BP and off-target kinases. **a** Rendering of CDK11-bound OTS964 (blue) in the cryo-EM map (shown as semi-transparent grey mesh and surface). **b**, **c** Active site views of the CDK11-cyclin L-SAP30BP-OTS964 complex in the two alternative conformations observed in the cryo-EM reconstruction. Hydrogen bonds (donor-acceptor distance 3.5 Å or less) are indicated by black dashed lines. The shortest distance between inhibitor and gatekeeper residue (M516 in CDK11) is indicated and shown by a white dashed line. The change in position of Y449 between (**b**, **c**) is indicated with a dashed arrow in (**b**). **d** Comparison of the structure of CDK11-cyclin L-SAP30BP-OTS964 (CDK11 in green, cyclin L in purple with residues 173–181 in pink, OTS964 in blue) to the structure of isolated CDK11B-OTS964 (PDB ID 7UKZ^31^; light green and light blue, respectively). The site of steric incompatibility of the isolated CDK11B conformation with the presence of cyclin L is indicated by a black triangle. Shifts in adjacent loops because of cyclin binding are indicated by arrows. **e** Active site view of the CAK-OTS964 complex (CDK7 is shown in grey, water molecules as red spheres). Only the hydrogen bonds to the hinge region are preserved because several hydrogen bonding partners are absent or beyond the hydrogen bonding distance (distance to D97 indicated by a yellow dashed line). The distance to the gatekeeper residue (F91 in CDK7) is enlarged (white dashed line). **f** Active site view of the CDK2-cyclin A2-OTS964 complex (CDK2 is shown in cyan). OTS964 forms three hydrogen bonds to CDK2, as it is within hydrogen bonding distance of CDK2 D86. Y15 is tucked under the dimethylamino-propyl group of OTS964.

The pose of OTS964 in our cryo-EM structure of the CDK11-cyclin L-SAP30BP complex (Fig. 6a and Supplementary Fig. 9b) is similar to the previously reported CDK11B-OTS964 X-ray crystal structure (PDB ID 7UKZ)^31^. The tricyclic ring system of the inhibitor is positioned such as to enable formation of three hydrogen bonds: two hydrogen bonds to the backbone carbonyl and amide nitrogen of the CDK11 hinge residue V519, and one hydrogen bond either to D522, which is a highly conserved residue among CDKs, or to E445 located within the N-terminal kinase lobe (Fig. 6b). Both D552 and E445 are within hydrogen bonding distance to the exocyclic hydroxy group of OTS964. Considering the expected ionisation state of carboxylic acids at physiological pH, only one of these two possible hydrogen bonds can be formed at a time, and E445 may engage in hydrophobic interactions with the nearby phenyl or dimethylamino groups of the inhibitor instead. D552, being the primary hydrogen-bonding partner in this interaction, is also consistent with the previous observation that the CDK11 E445G mutation did not impair OTS964 binding^31^.

The density for the G-rich loop in the OTS964-bound complex is weaker than neighbouring regions near the CDK11 active site. Interestingly, our cryo-EM density suggest that two alternative conformations of this loop exist. One of the conformations brings CDK11 residue Y449 to within hydrogen bonding distance of CDK11 K564, thereby bookending the dimethylamino group of OTS964 (Fig. 6c and Supplementary Fig. 9g, h). It is worth noting that phosphorylation of residues equivalent to Y449 in other CDKs (Y15 in CDK1/2) are known to serve to inactivate these kinases^2^. However, our mass spectrometry analysis (Supplementary Table 1 and Supplementary Data 1) did not detect phosphorylation of Y449 or the neighbouring residue T448 (T14 in CDK1/2). This suggests that the alternative G-loop conformations observed in our structure are likely to be independent of post-translational modifications of this region.

Compared to the X-ray crystal structure of the isolated CDK11B-OTS964 complex, there are modest motions within the CDK11 kinase domain upon association with cyclin L and SAP30BP (Fig. 6d). The motions of the αC-helix are minimal, but due to steric interference with cyclin L residues 173–181, the loop preceding the αC-helix is pushed towards the active site, which in turns impacts the conformation of the G-rich loop, with possible implications for binding of inhibitors and nucleotides. Importantly, the G-rich loop conformation in which Y449 points towards the inhibitor and K564 (Fig. 6c) was not observed in the X-ray crystal structure. Therefore, formation of the CDK11-cyclin L complex may influence inhibitor binding by affecting the conformational space that loop regions in the N-terminal kinase domain can access. Consistent with the modest effect of cyclin binding on OTS964 affinity observed in our ITC experiments (Supplementary Fig. 8), these conformational differences do not directly impact any of the key stabilising interactions between OTS964 and the CDK11 active site.

The pseudo-substrate segment of CDK11 is still visualised in the inhibitor-bound structure (Fig. 6b–d and Supplementary Fig. 9i), ruling out that nucleotide binding is required for it to assume this position. Indeed, applying limited *b*-factor sharpening of −10 Å^2^ and low-pass filtering the cryo-EM density of the OTS964-bound complex to 3.3 Å resolution shows further density extending from the pseudo-substrate segment towards its connection to the C-terminal kinase lobe (Supplementary Fig. 9j). Even though the connecting density is too weak to enable building of a reliable model, this observation supports the assignment of the density occupying the CDK11 substrate binding site to a sequence element of the kinase itself. Additionally, the position of the phosphate moiety in phosphorylated S572 coincides with the position of a sulphate molecule visualised in the CDK11-OTS964 X-ray crystal structure (PDB ID 7UKZ)^31^, further supporting the assignment of this density to a similarly negatively charged phosphate in our structure (Fig. 6d).

### Mechanism of OTS964 selectivity for CDK11 over other CDKs

Due to the high sequence and structural homology among the CDK family, the discovery of inhibitors that specifically target a single CDK poses a substantial challenge^1,43^. Therefore, we determined the structures of CAK-OTS964 and CDK2-cyclin A-OTS964, representing possible off-target complexes, to obtain further insight into OTS964 selectivity for CDK11 relative to other CDKs (Fig. 6e, f and Supplementary Fig. 10a–n). The CAK-OTS964 structure reveals conformational flexibility of the inhibitor in the CDK7 active site and the absence of several key contacts, including those formed by CDK11 E445 and D522. This explains the poor affinity of OTS964 for CDK7 (Supplementary Fig. 11a, b and Supplementary Note 1). Similarly, CDK2 is unable to contribute the stabilising interaction formed by CDK11 E445 with OTS964. Combined with differences in shape complementarity and the substitution of CDK11 G579 with an alanine in CDK2, this helps explain the comparably poor ability of OTS964 to inhibit CDK2 (Supplementary Fig. 11c–e and Supplementary Note 2).

## Discussion

**Fig. 7:**
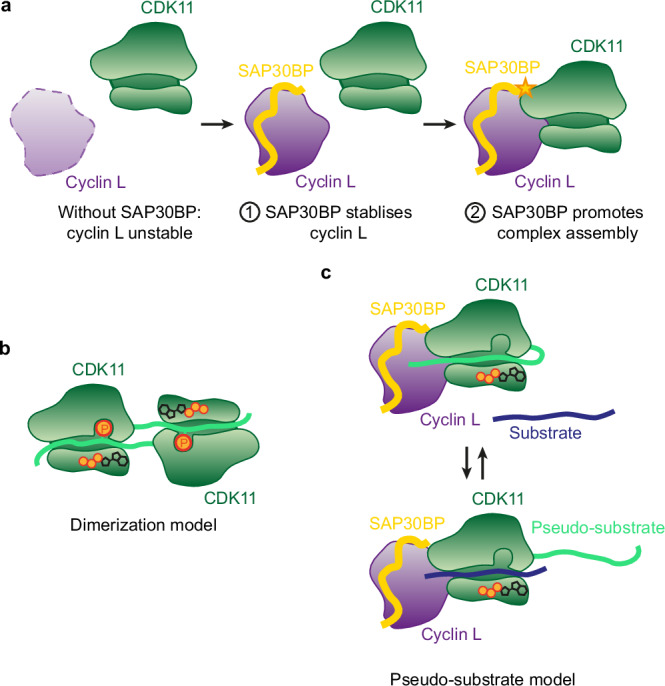
Roles of SAP30BP and the CDK11 pseudo-substrate in complex assembly and kinase regulation. **a** Schematic illustration of the dual role of SAP30BP in CDK11 activation by (1) stabilising cyclin L and (2) enhancing complex formation with CDK11. CDK11 and cyclin L are represented by green and purple shapes, and SAP30BP is represented by a yellow ribbon. **b** Model of how pseudo-substrate phosphorylation might lead to CDK11 dimerization. **c** Schematic illustration of the pseudo-substrate model applied to the binding of a simple model substrate to CDK11. The substrate binding site is schematically represented as a cleft, and the active site is indicated by a stylised ATP molecule. Release of the pseudo-substrate segment from the substrate-binding cleft is a prerequisite for substrate binding.

Taken together, our structural and biochemical data provide detailed insight into the molecular mechanisms governing CDK11 activity, regulation and inhibition. Our finding that cyclin L2 on its own is unstable—in agreement with prior results^19,31^—but becomes stabilised when expressed with its SAP30BP partner indicates that SAP30BP is a general cofactor for CDK11-cyclin L, in addition to its more specific functions in certain splicing pathways (Fig. 7a). This explains the unusually extensive but highly specific interaction interface between cyclin L and SAP30BP.

Furthermore, we have identified a pseudo-substrate within the C-terminal region of CDK11. This has possible implications for the regulation of CDK11 activity and substrate specificity. Our structures and mass spectrometry analysis indicate that this pseudo-substrate segment can be phosphorylated on S752, and that this phosphorylation can be accommodated in a pocket near the active site. Together with our data indicating that elimination of the negative charge from CDK11 residue 752 increases kinase activity in kinase assays, this raises the possibility that post-translational modifications might further modulate the regulatory effects of the pseudo-substrate (Supplementary Fig. 12a). However, the specific mechanisms by which CDK11 pseudo-substrate modifications might affect its biological function remain to be established. We note that phosphorylation of S752 within this pseudo-substrate segment has previously been linked to CDK11 dimerization^44^. While S752 phosphorylation might be able to promote the reciprocal binding of two CDK11 molecules to each other’s C-terminal segments, analogous to a domain-swap (Fig. 7b), our structural and biochemical results are more consistent with a pseudo-substrate function of this sequence (Fig. 7c).

The interplay between the CDK11 pseudo-substrate, its post-translational modification state, and its effects on the regulation of cellular processes is likely to be complex and will require further characterisation. More specifically, given our biochemical results, it is not immediately clear how S752 phosphorylation would enhance splicing in cellular assays as reported previously^44^. One hypothesis that might reconcile these observations is that some CDK11 substrates in the splicing pathway might contain binding sites for phosphorylated S752 and the surrounding CDK11 pseudo-substrate sequence. Binding to these substrates would withdraw the pseudo-substrate from its inhibitory position, thereby simultaneously de-repressing the kinase and aiding substrate binding (Supplementary Fig. 12b). Alternatively, modulation of the affinity of the pseudo-substrate to the CDK11 substrate binding site could fine-tune which substrates are preferentially phosphorylated by CDK11. S752 phosphorylation could thereby contribute to substrate specificity, in line with proposed models for the role of a phosphorylated pseudo-substrate segment in casein kinase 1 enzymes (ref. ^49^). In the context of this hypothesis, it is important to note that both spliceosomal CDK11 substrates such as SF3B1 (ref. ^18^) and an important transcriptional substrate^11^, the C-terminal Y_1_S_2_P_3_T_4_S_5_P_6_S_7_ heptapeptide repeat region of the RNA polymerase II subunit RPB1 (the so-called Pol II-CTD)^56^, contain multiple phosphorylation sites. Pre-existing phosphorylations in these targets could possibly enhance their ability to compete with the phosphorylated pseudo-substrate for CDK11 binding, thereby supporting their further phosphorylation (Supplementary Fig. 12c).

Finally, our structures of inhibitor-bound kinase complexes provide insight into OTS964 specificity for CDK11 and uncover conformational differences between the fully assembled CDK11-cyclin L-SAP30BP complex and the isolated CDK11B kinase domain. Thereby, we provide a structural framework for rational design and discovery of next-generation cancer therapeutics targeting CDK11.

## Methods

### Protein expression and purification

#### CDK11-cyclin L-SAP30BP

Synthetic, codon optimised genes encoding CDK11B, cyclin L2, and SAP30BP (Twist Bioscience) were cloned into 438-series vectors for baculovirus-based expression in insect cells^57^, resulting in a multi-protein construct expressing Twin-Strep-tagged CDK11B^p58^ (CDK11B^p110^ residues 357–795), His_6_-tagged Cyclin L2 (1–319), and MBP-tagged SAP30BP (Supplementary Fig. 1a, b; synthetic DNA sequences used in this study are provided in Supplementary Data 3). Assembled constructs were transformed into EMBacY cells for transposition into baculoviral bacmids^58^. Bacmids were prepared by isopropanol precipitation before transfection into Sf9 (*Spodoptera frugiperda*) insect cells (Thermo Fisher Scientific, Cat. no. 11496015) using Insect GeneJuice Transfection Reagent (Sigma-Aldrich). After two rounds of baculovirus amplification, 20 mL of insect cell culture supernatant was used to infect 1-L cultures of High5 (*Trichoplusia* ni) insect cells (Thermo Fisher Scientific, Cat. no. B85502). After 72 h of incubation, insect cells were harvested and stored at −80 °C for later use.

The CDK11-cyclin L-SAP30BP complex was purified by Strep-Tactin-affinity and size exclusion chromatography (SEC). Cells were thawed and resuspended in purification buffer (250 mM KCl, 2 mM MgCl_2_, 40 mM HEPES-KOH pH 7.9, 5 mM β-mercaptoethanol, 10% (v/v) glycerol) supplemented with protease inhibitors and DNase I before lysis by successive cycles of freeze-thaw. Cell debris were pelleted by centrifugation at 18,000 rpm (approx. 26,600 × *g*) for 30 min at 4 °C in a JA-25.50 rotor (Beckman Coulter), and the cleared lysate was incubated for 45 min with 2.5 mL of Strep-Tactin Superflow Plus resin (Qiagen). Beads were washed in purification buffer, and purified protein was eluted in purification buffer supplemented with 10 mM desthiobiotin (Sigma-Aldrich). Affinity tags were cleaved by Tobacco Etch Virus (TEV) protease at 4 °C for 1 h before proceeding directly to SEC using a Superdex 200 Increase 10/300 GL column (GE Healthcare) in size exclusion buffer (200 mM KCl, 2 mM MgCl_2_, 20 mM HEPES-KOH pH 7.9, 5 mM β-mercaptoethanol, 5% (v/v) glycerol). Peak fractions were pooled, concentrated to approx. 2 mg/mL, snap frozen in liquid N_2_, and transferred to −80 °C for long-term storage.

#### CDK11 and cyclin L-SAP30BP for pull-down assays

An expression construct encoding Twin-Strep-tagged CDK11B^p58^ (CDK11B^p110^ residues 357–795) was cloned and used to prepare bacmids, which were then transfected into Sf9 insect cells (Thermo Fisher Scientific). Two multi-expression constructs were also cloned for expression of His_6_-tagged cyclin L2 (residues 1–319) and either the WT or 4A mutant (K157A, R160A, N161A, K167A) of MBP-tagged SAP30BP and similarly used to prepare bacmids. After transfection and two rounds of baculovirus amplification, 20 mL (cyclin L2-SAP30BP) or 40 mL (CDK11B) of insect cell culture supernatant was used to infect 1-L (cyclin L2-SAP30BP) or 2-L (CDK11B) cultures of High5 insect cells (Thermo Fisher Scientific). After 72 h of incubation, insect cells were harvested and stored at −80 °C for later use.

Strep-tagged CDK11B^p58^ was purified as described for the CDK11-cyclin L-SAP30BP complex above, only omitting the cleavage of the affinity tags. WT and mutant cyclin L2 (1–319)-SAP30BP complexes were purified by nickel-affinity and gel filtration chromatography. Cells were thawed and resuspended in purification buffer (250 mM KCl, 2 mM MgCl_2_, 40 mM HEPES-KOH pH 7.9, 5 mM β-mercaptoethanol, 10% (v/v) glycerol, 10 mM imidazole) supplemented with protease inhibitors and DNase I before lysis by successive cycles of freeze-thaw. Cell debris were pelleted by centrifugation at 18,000 rpm (approx. 26,600 × *g*) for 30 min at 4 °C in a JA-25.50 rotor (Beckman Coulter), and cleared lysate was incubated for 45 min with 2.5 mL of Ni-NTA Superflow resin (Qiagen). Beads were washed in wash buffer (purification buffer with 25 mM imidazole), and purified protein was eluted in elution buffer (purification buffer with 300 mM imidazole). Affinity tags were cleaved by TEV protease at 4 °C for 1 h before proceeding directly to SEC, which was performed on a Superdex 200 Increase 10/300 GL column (GE Healthcare) as described above. Peak fractions were pooled, concentrated to approx. 2 mg/mL, snap frozen in liquid N_2_, and transferred to −80 °C for long-term storage.

#### CDK11-cyclin L-SAP30BP complexes for kinase assays

CDK11^p58^ complexes for kinase assays were expressed in insect cells. Point mutations were cloned into an existing construct of CDK11B^p58^ in a 438-series vector, and the truncation mutant (CDK11 ∆C, corresponding to CDK11B^p110^ residues 357–743) was generated through the introduction of a premature stop codon. Complete complexes were obtained by infecting Sf9 insect cells with bacmid DNA to generate separate baculoviruses encoding either Strep-tagged CDK11B^p58^ (WT or mutant) or His_6_-tagged cyclin L2 (residues 1–319) and MBP-tagged SAP30BP. After two rounds of baculovirus amplification, these were combined for co-expression in High5 insect cells by inoculating 1-L cultures with 20 mL of each baculovirus. After 72 h of incubation at 27 °C, cultures were harvested and pellets stored at −80 °C for later use.

WT and mutant complexes were purified by Strep-Tactin affinity and gel filtration chromatography as described for the CDK11-cyclin L-SAP30BP complex used for structure determination, with affinity tags cleaved by incubation with TEV protease. Peak fractions were pooled, concentrated to approx. 2 mg/mL, snap frozen and stored at −80 °C for later use.

#### CDK11 for ITC

CDK11B^p58^ (monomeric or in-complex) for ITC were expressed in insect cells. The complete complex was expressed from the multi-expression construct described above for structure determination, containing Twin-Strep-tagged CDK11B^p58^, His_6_-tagged Cyclin L2 (1–319), and MBP-tagged SAP30BP. Monomeric CDK11B^p58^ was expressed from the construct containing only Twin-Strep-tagged CDK11B^p58^, described above for use in pull-down assays. Two liter cultures of High5 insect cells were inoculated with 40 mL of baculovirus (multi-protein or monomeric CDK11). After 72 h of incubation at 27 °C, cultures were harvested and pellets stored at −80 °C for later use.

Proteins were purified by Strep-Tactin affinity and gel filtration chromatography as described for the CDK11-cyclin L-SAP30BP complex used for structure determination, with affinity tags cleaved by incubation with TEV protease, with only minor changes. Size exclusion buffer was adjusted for ITC (200 mM KCl, 2 mM MgCl_2_, 20 mM HEPES-KOH pH 7.9, 1 mM Tris (2-carboxyethyl) phosphine hydrochloride (TCEP), 4% (v/v) glycerol). Peak fractions were pooled, concentrated to approx. 3 mg/mL (monomeric) or 6 mg/mL (complex), snap frozen and stored at −80 °C for later use.

#### SF3B1 for kinase assays

GST-SF3B1 (SF3B1 residues 1–463) was expressed from a synthetic, codon-optimised plasmid in a pGEX-4T-1 backbone (Genscript) in bacterial *E. coli* BL21-RIL cells. Two 10 mL cultures of LB containing 100 µg/mL ampicillin and 34 µg/mL chloramphenicol were inoculated with a single colony of BL21-RIL cells transformed with pGEX-4T-1-SF3B1^1–463^ and grown overnight at 37 °C with vigorous shaking. Two 1-L cultures of LB containing 100 µg/mL ampicillin and 34 µg/mL chloramphenicol were each inoculated with the entirety of a 10 mL overnight culture and incubated at 37 °C until the optical density (OD) had reached 0.6. Cultures were induced with 0.5 mM isopropyl-thiogalactoside (IPTG) and incubated at 37 °C for a further 4 h. Cultures were harvested by spinning at 6000 × *g* for 18 min at 4 °C using a JLA 8.1 rotor, and pellets were snap frozen in liquid nitrogen and stored at −80 °C for later use.

GST-SF3B1^1–463^ was purified by GST-affinity resin and SEC. Pellets were thawed and resuspended in purification buffer (150 mM NaCl, 2 mM MgCl_2_, 50 mM HEPES-KOH pH 7.5, 5 mM β-mercaptoethanol, 10% (v/v) glycerol) supplemented with protease inhibitors and DNase I before lysis by sonication. Cell debris were pelleted by centrifugation at 18,000 rpm (approx. 26,600 × *g*) for 30 min at 4 °C in a JA-25.50 rotor (Beckman Coulter), and the cleared lysate was incubated for 45 min with 2.5 mL of Glutathione Sepharose 4B resin (Cytiva) at 4 °C. Beads were washed in purification buffer and ATP-wash buffer (5 mM ATP, 150 mM NaCl, 20 mM MgCl_2_, 50 mM HEPES-KOH pH 7.5, 5 mM β-mercaptoethanol, 10% (v/v) glycerol). Protein was eluted in purification buffer supplemented with 10 mM reduced glutathione (Sigma) before proceeding directly to SEC, which was performed using a Superdex 200 Increase 10/300 GL column (GE Healthcare) in size exclusion buffer (150 mM NaCl, 2 mM MgCl_2_, 20 mM HEPES-KOH pH 7.9, 5 mM β-mercaptoethanol, 5% (v/v) glycerol). Peak fractions were pooled, concentrated to approx. 5 mg/mL, snap frozen in liquid N_2_, and transferred to −80 °C for long-term storage.

#### CDK7-cyclin H-MAT1(Δ219)

Structure determination of the CAK-OTS964 complex employed a construct lacking the N-terminal, flexibly attached helical and RING domains of MAT1 (ref. ^33^), removal of which had previously been observed to improve the orientation distribution of CAK particles in cryo-EM experiments^59^. The complex was expressed and purified as described above for CDK11-cyclin L-SAP30BP, except that a nickel affinity purification step using Ni-NTA Superflow beads (Qiagen) was added before incubation with the Strep-Tactin Superflow resin, and the cleaved tags and TEV protease were removed by incubation with Ni-NTA Superflow beads before gel filtration.

#### CDK2-cyclin A2

CDK2 and cyclin A2 were expressed separately in Rosetta DE3 pLysS cells. CDK2 was expressed with an N-terminal His_10_-tag (Addgene plasmid #79726; http://n2t.net/addgene:79726; RRID:Addgene_79726, provided by John Chodera, Nicholas Levinson and Markus Seeliger)^60^, and cyclin A2 was expressed with an N-terminal His_6_-tag. The human cyclin A2 sequence was provided by Jonathon Pines, and the 15-H bacterial expression vector was a gift from Avinash Patel. For protein expression, Rosetta DE3 pLysS cells harbouring the individual expression constructs were grown at 37 °C to OD ~0.6, induced with 1 mM IPTG, and then incubated at 24 °C overnight. The cultures were harvested by centrifugation, frozen in liquid nitrogen, and stored at −80 °C.

For protein purification, cell pellets of CDK2- and cyclin A2-expressing cells were combined and resuspended in purification buffer (50 mM HEPES pH 7.5, 180 mM NaCl, 5 % (v/v) glycerol, 2 mM MgCl_2_, 2 mM DTT) supplemented with protease inhibitors and DNase I. The cells were lysed by sonication, and the lysate was clarified by two centrifugation steps at 18,000 rpm (approx. 26,600 × *g*) in a JA-25.50 rotor at 4 °C. The cleared lysate was supplemented with 10 mM imidazole and loaded onto a 5 mL HisTrap column (Cytiva), which was then attached to an Akta Pure M25 instrument (Cytiva), washed using purification buffer supplemented with 20 mM imidazole, and eluted using a linear imidazole gradient from 20 to 300 mM across 12 column volumes. The column was further eluted using 30 mL of buffer at 300 mM, and fractions containing both proteins were combined, concentrated to 4 mg/mL, and incubated with TEV protease at 4 °C overnight to cleave off the His_6/10_ tags. After incubation with Ni-NTA Superflow beads (Qiagen) for removal of the cleaved tags and the TEV protease, the supernatant was concentrated to 1.5 mL, frozen in liquid nitrogen, and stored overnight. This frozen protein aliquot was thawed, and the protein complex was then further purified using a Superdex 200 Increase 10/300 column (Cytiva). Peak fractions were combined, yielding a concentration of approx. 2 mg/mL, and flash frozen in liquid nitrogen for storage at −80 °C.

### Pull-down assay with and without cyclin L-SAP30BP co-expression

Cloning was performed to generate 438-series vectors for insect cell expression encoding His_6_-tagged cyclin L2, MBP-tagged SAP30BP and Strep-tagged CDK11B^p110^. A multi-protein co-expression construct was also made containing all three subunits with their respective affinity tags in one vector. These constructs were used to prepare bacmids and then transfected into Sf9 insect cells to generate baculoviruses. After one round of baculovirus amplification, a 50 mL culture of Sf9 cells was inoculated for each co-expression test. For expressions of cyclin L2 alone, or cyclin L2 with one other protein subunit, the culture was inoculated with 1 mL of each virus encoding a protein (1–2 mL). For the co-expression of all three proteins, 1 mL of baculovirus encoding the single multi-protein construct was added to the culture. Inoculated cultures were incubated at 27 °C for 72 h, and cell pellets were harvested by spinning at 1000 × *g* for 15 min, before being stored at −80 °C for later use.

The pull-down assay was performed using the nickel-binding capability of His_6_-tagged cyclin L2. Pellets were resuspended in 3 mL of assay buffer (250 mM KCl, 2 mM MgCl_2_, 40 mM HEPES-KOH pH 7.9, 5 mM β-mercaptoethanol, 10% (v/v) glycerol, 10 mM imidazole), and 1.25 mL of this resuspension was then used in the pull-down. Resuspended cells were lysed by three successive freeze-thaw cycles, and the lysate cleared by spinning at 20,000 × *g* for 10 min at 4 °C. Cleared lysate was incubated with 80 µL Ni-NTA Superflow resin for 15 min at 4 °C. Three washes were performed with 1 mL of wash buffer (assay buffer supplemented with imidazole to 25 mM). Bound proteins were eluted in 100 µL of elution buffer (assay buffer supplemented with imidazole to 300 mM). Twenty-microliter samples were analysed by SDS-PAGE.

### Pull-down assays with purified proteins and mutant SAP30BP

Pull-down assays were performed using Strep-Tactin binding affinity of purified Twin-Strep-tagged CDK11^p110^. Nine micrograms of purified Twin-Strep-tagged CDK11^p110^ was incubated with 200 µL Strep-Tactin Superflow Plus resin (Qiagen) for 30 min at 4 °C. Two washes with 750 µL binding buffer (250 mM KCL, 2 mM MgCl_2_, 40 mM HEPES-KOH, 10% glycerol, 5 mM β-mercaptoethanol) were performed. CDK11-bound resin was then incubated with 27 µg of purified cyclin L-SAP30BP (WT or 4A mutant) for 15 min at 4 °C. Two washes with 750 µL binding buffer were performed. Bound proteins were eluted in 100 µL of binding buffer supplemented with 10 mM desthiobiotin (Sigma-Aldrich). Twenty-microliter samples were analysed by SDS-PAGE.

### Pull-down assay with cyclins H, K and T

Synthetic, codon-optimised genes for cyclins H, K and T1 were cloned into 438-series vectors for insect cell expression, resulting in constructs encoding each cyclin with an N-terminal His_6_ tag. An additional construct was prepared encoding MBP-tagged SAP30BP. Constructs were transformed into EMBacY cells for transposition into baculoviral bacmids, and the resulting bacmids were used to transfect Sf9 cells for production of baculovirus. After one round of baculovirus amplification, pull-down co-expressions were made by infecting 50 mL cultures of Sf9 insect cells with 1 mL each of virus encoding MBP-tagged SAP30BP and one of the His_6_-tagged cyclin variants. After cultures were incubated for 72 h at 27 °C, pellets were harvested by centrifugation at 1000 x *g* for 10 min and stored at −80 °C for later use.

Separate pull-down assays were performed using nickel and amylose- affinity resin. Co-expression cell pellets were resuspended in 3 mL assay buffer (250 mM KCl, 2 mM MgCl_2_, 40 mM HEPES-KOH pH 7.9, 5 mM β-mercaptoethanol, 10% (v/v) glycerol) and this was then divided into 1.5 mL for each of the different affinity pull-downs. The nickel pull-down suspension was supplemented with imidazole to a concentration of 10 mM. Cells were lysed by 3 successive freeze-thaw cycles of resuspended pellets, and lysate was cleared by spinning at 20,000 × *g* for 15 min at 4 °C. Cleared lysate was incubated with 200 µL Ni-NTA Superflow resin or 500 µL amylose resin, rotating for 30 min at 4 °C. Three 1 mL washes were performed using assay buffer (supplemented with 25 mM imidazole for Ni affinity pull-downs). Bound proteins were eluted in 100 µL of assay buffer supplemented with 300 mM imidazole (nickel-affinity pull-downs) or 200 µL of assay buffer supplemented with 10 mM maltose. Twenty-microliter samples were analysed by SDS-PAGE.

### Kinase assays for pseudo-substrate characterisation

Fifty nanomolar CDK11^p58^-cyclin L (1–319)-SAP30BP complex (WT or mutant) was incubated with 6 µM GST-SF3B1^1–463^ and 2 mM ATP in a total volume of 100 µL, in assay buffer (150 mM NaCl, 25 mM HEPES-KOH pH 7.9, 9 mM MgCl_2_, 2 mM DTT, 2.5% (v/v) glycerol) for 60 min at RT. Samples were taken at 0-, 10-, 20-, 30- and 60 min time points, diluted 1:10 in assay buffer, and quenched by combination with SDS-loading dye. *N* = 3 biological replicates were performed for each CDK11 complex. Fifteen microliter samples were analysed by SDS-PAGE using Nu-PAGE 4–12% Bis-Tris gels (Thermo Fisher Scientific) such that all time points from all four complexes were run across two gels, for each repeat, with the WT 60 min time point loaded on both gels for normalisation.

Western blotting was performed following transfer to nitrocellulose membranes. Membranes were stained using Ponceau S and imaged using a ChemiDoc Imaging System (BioRad; running the Image Lab Touch software, version 3.0.1.14) to obtain loading controls. Membranes were destained in PBST (1x phosphate-buffered saline (PBS) supplemented with 0.1% v/v Tween 20), then blocked in 5% BSA/PBST (5% w/v bovine serum albumin dissolved in PBST) at 4 °C overnight. Primary incubation was performed for 1 h at RT using a rabbit anti-phospho-SF3B1 (Thr313) primary antibody (Cell Signalling Technology, Cat. no. 25009, clone D8D8V, lot no. 1) diluted 1:2000 in 5% BSA/PBST. Membranes were washed in PBST before incubation with a goat anti-rabbit secondary antibody conjugated to IRDye 800CW (LICORbio, Cat. no. 926-32211, lot no. D50528-08, RRID: AB_2651127) diluted 1:20,000 in 5% BSA/PBST. Membranes were washed in PBST, then imaged using the Odyssey CLx imaging system (LICORbio, running Image Studio version 5.2.5), using the 800 nm channel.

Band intensities were quantified using ImageJ 1.54 g^61^ and blot bands were normalised to those of loading controls (SF3B1 substrate). The WT 60 min signal was set to a relative intensity of 1 for each replicate. Statistical analysis of *N* = 3 biological replicates was performed by 2-way ANOVA and Šídák’s multiple comparisons test, as implemented in PRISM (version 10.6.1; GraphPad Software), comparing either CDK11B^p58^ WT vs. ∆C or S752E vs. S752A.

### Enzyme inhibition assays

In-vitro kinase assays of OTS964 with CDK11-cyclin K, CDK2-cyclin A2 and CDK7-cyclin H-MAT1 were performed by Reaction Biology GmbH (Freiburg, Germany). The assays were performed as radiometric kinase assays, measuring incorporation of ^33^P from [γ-^33^P]-ATP. ATP concentrations were set to the apparent ATP-K_m_ of the respective enzyme. Reactions were run for 60 min at 30 °C before quenching with 2% (v/v) H_3_PO_4_. Incorporation of ^33^P_i_ was determined using a microplate scintillation counter. Raw data were normalized using high controls (full enzyme activity without inhibitor) and low controls (unspecific binding of radioactivity in the absence of both enzyme and inhibitor) on the same plates. Data were analysed in PRISM (version 10.3; GraphPad Software) as inhibitor concentration vs. normalized response with variable slope. Asymmetric confidence intervals were computed at a significance level of 95%.

### Phosphoproteomics analysis

The CDK11^p110^-SAP30BP-cyclin L2 sample (approx. 24 μg) was adjusted with triethylammonium bicarbonate buffer (TEAB) at a final concentration of 100 mM. Proteins were reduced and alkylated with 5 mM tris-2-carboxyethyl phosphine (TCEP) and 10 mM iodoacetamide (IAA) simultaneously for 60 min in the dark and digested overnight with trypsin at a final concentration of 50 ng/μL (Pierce). The sample was dried, and peptides were cleaned up with desalting spin columns (Pierce) followed by phosphopeptide enrichment with the High Select Fe-NTA kit (Pierce) according to the manufacturer’s instructions. Both the eluate and flow-through solutions were kept for LC-MS analysis.

LC-MS analysis was performed on a Dionex UltiMate 3000 UHPLC system coupled with an Orbitrap Ascend Mass Spectrometer (Thermo Fisher Scientific; software used for data acquisition: Xcalibur, version 4.7.69.37 and Orbitrap Ascend Tune Application, version 4.2.4321). Peptides were reconstituted in 30 μL 0.1% trifluoro-acetic acid (TFA), and 15 μL (eluate) or 3 μL (flow-through) were loaded to the Acclaim PepMap 100, 100 μm × 2 cm C18, 5 μm trapping column at 10 μL/min flow rate of 0.1% TFA loading buffer. Peptides were then subjected to a gradient elution on a capillary column (Waters, nanoE MZ PST BEH130 C18, 1.7 μm, 75 μm × 250 mm) connected to the EASY-spray source at 45 °C with an EASY-spray emitter (Thermo Fisher Scientific, Cat. no. ES991). Mobile phase A was 0.1% formic acid, and mobile phase B was 80% acetonitrile, 0.1% formic acid. The separation method at a flow rate of 300 nL/min was an 80 min gradient from 3 to 26% B. Precursors between 375 and 1500 m/z and charge states 2–7 were selected at 120,000 resolution in the top speed mode in 3 s and isolated for HCD fragmentation (collision energy 32%) with quadrupole isolation width 0.7 Th, Orbitrap detection with 30,000 resolution and 59 ms maximum injection time. Targeted MS precursors were dynamically excluded from further isolation and activation for 45 s with 10 ppm mass tolerance.

Peptide and protein identification was conducted in Proteome Discoverer (Thermo Fisher Scientific; version 3.0.1.27) with the Sequest HT search engine. Precursor and fragment mass tolerances were 20 ppm and 0.02 Da, respectively with a maximum of 2 trypsin missed cleavages allowed. Dynamic modifications included carbamidomethyl at C, oxidation of M, deamidation of N/Q, and phosphorylation of S/T/Y. Spectra were searched against a FASTA file containing reviewed *Homo sapiens* UniProt entries. Peptides were filtered at *q* < 0.01 using the Percolator node and target-decoy database search. Phosphorylation localization probabilities were estimated with the IMP-ptmRS node.

### Isothermal titration calorimetry

ITC was performed on a Malvern MicroCal Auto-iTC200 at 25 °C. Data were acquired using the ITC200 software, version 1.24.2 (MicroCal, LLC). CDK11^p58^ or CDK11^p58^-cyclin L2 (1–319)-SAP30BP was prepared in ITC buffer to a final composition of 37 µM protein, 200 mM KCl, 2 mM MgCl_2_, 20 mM HEPES-KOH pH 7.9, 1 mM TCEP, 2.5% (v/v) glycerol, 2% (v/v) DMSO. OTS964 (in 100% DMSO) was diluted to 370 µM in ITC buffer to achieve the same buffer composition. Three hundred seventy micromolar OTS964 (syringe) was titrated into 37 µM CDK11 (monomeric or in-complex) (cell) by 38 injections of 1 µL with 180 s spacing between injections. Two independently measured technical replicates for each protein were analysed using Origin 7 (v7.0552).

### Cryo-EM sample preparation

Cryo-EM samples containing CDK11-cyclin L-SAP30BP were prepared on UltrAuFoil R1.2/1.3 holey gold grids (Quantifoil Microtools). Purified protein complex was diluted to approx. 0.4 mg/mL in dilution buffer (200 mM KCl, 2 mM MgCl_2_, 40 mM HEPES-KOH pH 7.9) and combined with 1 mM AMP-PNP (Sigma-Aldrich) or 25 µM OTS964 (MedChemExpress; dissolved at 25 mM in 100% DMSO), in the presence of a 200 µM of a 21-mer cryo-peptide (sequence MALKVTKNSKINAENKAKINM) on ice. The cryo-peptide was included because the complex showed extremely high preferred orientation when grids were prepared using conventional methods. Four microliters of sample were applied to plasma-cleaned grids (Tergeo-EM plasma cleaner, PIE Scientific), mounted in a Vitrobot Mark IV (Thermo Fisher Scientific) operated at 5 °C and 100% humidity, blotted for 1, 1.5 or 2 s and plunged into liquid ethane at liquid N_2_ temperature. Subsequently, the grids were clipped into autogrid cartridges (Thermo Fisher Scientific) compatible with the Glacios and Krios autoloader systems.

Cryo-EM samples of CAK-OTS964 and CDK2-cyclin A-OTS964 complexes were prepared as described above, except that 50 µM OTS964 were used to overcome the lower affinity for the off-target kinases. No peptide additive was used for the CAK-containing complex.

### Initial cryo-EM screening

CDK11-cyclin L-SAP30BP grids were screened on a Glacios cryo-transmission electron microscope (cryo-TEM) operated at 200 kV acceleration voltage and equipped with a Falcon 4i direct electron detector (Thermo Fisher Scientific). Data were acquired using the EPU software (Thermo Fisher Scientific) in EER format at a pixel size of 0.5675 Å/pixel and a total exposure of 60 electrons/Å^2^, using aberration-free image shift (AFIS) to accelerate the data collection. Five hundred or more movies, each fractionated into 40 frames for motion correction, were processed on the fly using cryoSPARC live (version 4.4.1)^62^, enabling assessment of particle quality and orientation distribution by streaming 2D classification and streaming 3D refinement. Suitable grids were progressed to high-resolution data collection.

### High-resolution cryo-EM data collection

For high-resolution structure determination, data were acquired on Titan Krios G2 and G3i cryo-TEMs (Thermo Fisher Scientific) operated at 300 kV acceleration voltage and equipped with a K3 camera and a BioQuantum energy filter (controlled using Digital Micrograph, version 3.53.41360, all Gatan Inc.). Data were collected using the EPU software (versions 3.4 and 3.7; Thermo Fisher Scientific) in TIFF format at ×165,000 nominal magnification using the hardware binning mode of the K3 camera, resulting in a pixel size of 0.504 Å/pixel (AMP-PNP complex) or 0.51 Å/pixel (OTS964-containing complexes). Movies were acquired using a defocus range of −0.8 to −1.8 µm and a total electron exposure of 70–72 electrons/Å^2^, fractionated into approx. 70 frames. AFIS and fringe-free imaging were used for acceleration of the data collection. Grid squares were selected manually and brought to eucentric height using EPU (Thermo Fisher Scientific), before automated detection of holes and subsequent hole selection by grid-specific filtering of ice thickness. For the CDK11-cyclin L-SAP30BP complex bound to AMP-PNP, 16,936 and 19,396 exposures were acquired from two grids (referred to as AM4-3 and BG210-3 below); for the OTS964-bound CDK11-cyclin L-SAP30BP complex 13,829 exposures from one grid (AM10-3); for the CAK-OTS964 complex 10,788 exposures from one grid (BG61-1); and for the CDK2-cyclin A-OTS964 complex 15,468 exposures from one grid (BG65-1).

### Cryo-EM data processing

Cryo-EM data of AMP-PNP-bound CDK11-cyclin L-SAP30BP were initially processed in cryoSPARC live (version 4.4.1)^62^ before transfer to RELION^63^ (version 5.0-beta incorporating BLUSH regularisation^64^) for additional processing and high-resolution 3D refinement (Supplementary Fig. 13). In cryoSPARC live, raw movies were motion corrected with 2× binning, resulting in pixel sizes of approx. 1 Å/pixel in the motion-corrected micrographs. Following inspection of CTF fit, relative ice thickness and max. per-frame motion, lower-quality micrographs were rejected. Particle picking used circular and elliptical blob settings (80–110 Å diameter) to extract particles in a box size of 160 × 160 pixels before Fourier cropping to 80 × 80 pixels, resulting in approx. 2 Å pixel size in the extracted particles. Following streaming 2D classification and streaming 3D refinement during live processing, additional 2D classification to maximise retrieval of high-quality particles, particularly from rare views, was performed. Particles from 8 to 10 good classes obtained from streaming 2D classification during the cryoSPARC live session were re-classified into 50 2D-classes using a batch size of 300 particles for 100 iterations. In parallel, all extracted particles also underwent re-classification into 100 2D-classes with a batch size of 300 particles for 100 iterations. Particles of selected good classes from both re-classifications were combined and duplicate particles removed before re-extracting and re-centring of the final particle set. The metadata of the particles selected by this procedure (approx. 1,600,000 from AM4-3 and approx. 2,600,000 from BG210-4) were exported and converted to RELION-compatible *.star files using PyEM programs^65^ before import into RELION 5.0.

In RELION 5.0, particles were re-extracted from the cryoSPARC motion-corrected micrographs using coordinate information contained in the imported particles. Extracted particles then underwent masked 3D auto-refinement using a previous reconstruction of the same complex from Glacios screening as an initial model and with BLUSH regularisation^64^ enabled, reaching ~2.5 Å resolution. Particles were then subjected to 3D classification with no alignment (regularisation parameter *τ* = 20) and 3D auto-refined again. For all but one dataset (CDK11-cyclin L-SAP30BP grid AM4-3), the remaining particle images were re-extracted at a larger box size (192 × 192 pixels) to prevent aliasing of the high resolutions in Fourier space. For the data from grid AM4-3, another 3D classification step was inserted before re-extraction to further improve the quality of the retained particles (Supplementary Fig. 13). Subsequently, CTF refinement (beam tilt and trefoil) was applied, followed by high-resolution 3D auto-refinement. For the CDK11-cyclin L-SAP30BP reconstruction in complex with AMP-PNP, data from two grids were merged at this point, leading to a further resolution increase.

All other structures (i.e. the OTS964-bound complexes) were each determined from a single dataset collected on a single grid. For these datasets, CTF refinement was applied twice because of unexpectedly large beam tilt values that initially limited the obtained resolution. Data processing workflow schemes for the inhibitor-bound complexes are provided in Supplementary Figs. 14–16.

Map resolution assessment by Fourier shell correlation (FSC), orientation distribution plots, and assessment of resolution anisotropy using the 3D FSC validation server^66^ are provided in Supplementary Figs. 2, 9 and 10.

### Model building and refinement

Atomic models were iteratively built in COOT^67^ and refined using the real-space refinement algorithm in PHENIX^68^. Starting models for atomic model building were generated using AlphaFold3^32^ for CDK11-cyclin L-SAP30BP or from deposited structures of human CAK^59^ and CDK2-cyclin A^69^. Refinement restraints for the OTS964 ligand were generated in PHENIX ELBOW^70^. Refined models were validated using MOLPROBITY^71^ as implemented in PHENIX^72^. Refinement statistics are provided in Supplementary Tables 2 and 3.

### AlphaFold structure predictions

Initial AlphaFold 3-based structure predictions of the CDK11-cyclin L-SAP30BP complex used the full-length sequences of human CDK11B^p110/p58^, cyclin L2 and SAP30BP (UniProt accession codes P21127, Q96S94 and Q9UHR5) and were obtained using the AlphaFold 3 web server^32^.

To generate structure predictions of all human CDKs to assess the presence of pseudosubstrates, sequences for the 20 human CDKs and their partner cyclins were retrieved from UniProt (accession codes: CDK1: P06493; CDK2: P24941; CDK3: Q00526; CDK4: P11802; CDK5: Q00535; CDK6: Q00534; CDK7: P50613; CDK8: P49336; CDK9: P50750; CDK10: Q15131; CDK12: Q9NYV4; CDK13: Q14004; CDK14: O94921; CDK15: Q96Q40; CDK16: Q00536; CDK17: Q00537; CDK18: Q07002; CDK19: Q9BWU1; CDK20: Q8IZL9; cyclin A: P20248; cyclin B: P14635; cyclin C: P24863; cyclin D: P24385; cyclin H: P51946; cyclin K: O75909; cyclin Q: Q8N1B3; cyclin T: O60563; cyclin Y: Q8ND76). For CDK11, sequences were matched to those used in our experimental structural determination (accession codes CDK11B: P21127, residues 357–795; cyclin L2: Q96S94, residues 1–319; SAP30BP: Q9UHR5). Predictions for each CDK-cyclin complex obtained using the AlphaFold3 web server^32^. For each predicted complex, all 5 predicted models were visually assessed for the presence of predicted pseudo-substrates occupying the substrate binding site using USCF Chimera X^73^ and the PAE plot generated by the AlphaFold3 web server.

### Analysis and depiction of structures

Buried surface areas at protein–protein interfaces were computed using the PDBePISA web server at the European Bioinformatics Institute (http://www.ebi.ac.uk/pdbe/prot_int/pistart.html)^39^. Figures depicting molecular complexes were generated using USCF Chimera X^73^ and PyMOL (The PyMOL Molecular Graphics System, version 2.5.2-2.5.6, Schrodinger LLC).

### Statistics and reproducibility

Kinase assays were performed in three biological replicates. ITC measurements were conducted as two technical replicates. Pull-down assays using purified proteins were performed as two technical replicates. Co-expression followed by small-scale co-purification was performed as two biological replicates. Uncropped SDS-PAGE gels and Western blot membranes are provided as Source data.

### Reporting summary

Further information on research design is available in the Nature Portfolio Reporting Summary linked to this article.

## Supplementary information

Supplementary Information

Peer Review File

Description of Additional Supplementary Files

Supplementary Data 1

Supplementary Data 2

Supplementary Data 3

Reporting Summary

## Source data

Source Data

## Data Availability

The cryo-EM maps and atomic coordinate models for CDK11-cyclin L-SAP30BP-AMPNP, CDK11-cyclin L-SAP30BP-OTS964 (1 EMDB and 2 PDB entries), CAK-OTS964, and CDK2-cyclin A-OTS964 structures have been deposited in the EMDB with accession codes EMD-53224, EMD-53221, EMD-53205 and EMD-53204 and in the PDB with accession codes 9QKZ, 9QKT, 9QL1, 9QJN and 9QJJ. Maps with reduced sharpening *B*-factor (*B* = −10) used in some figures for visualisation of more dynamic protein segments have been supplied as additional maps within the EMDB entries of the CDK11B-cyclin L2-SAP30BP structures. Mass spectrometry data have been deposited to the ProteomeXchange Consortium via the PRIDE partner repository with accession code PXD060582. Uncropped Western blot membranes, uncropped SDS-PAGE gels, and data points used for line graphs and bar charts are provided as Source data. Source data are provided with this paper.
