# Supplementary material for: Cryo-EM structures of the CDK11-cyclin L-SAP30BP complex reveal mechanisms of CDK11 regulation
Source: Nat Commun. 2026 Apr 25;17:5718. doi: 10.1038/s41467-026-72329-4 (PMC13324802; doi:10.1038/s41467-026-72329-4)
Supplement: Supplementary file 1 — Supplementary Information [file 41467_2026_72329_MOESM1_ESM.pdf]

Supplementary Information

Cryo-EM structures of CDK11-cyclin L-SAP30BP complexes reveal mechanisms of CDK11 regulation

Amy J. S. McGeoch, Victoria I. Cushing, Theodoros I. Roumeliotis, Nora B. Cronin, Stephen J. Hearnshaw, Jyoti S. Choudhary, Claudio Alfieri, Basil J. Greber*

* correspondence to: basil.greber@icr.ac.uk (B.J.G.)

This file includes:
Supplementary Figures 1-16
Supplementary Tables 1-3
Supplementary References 1-10

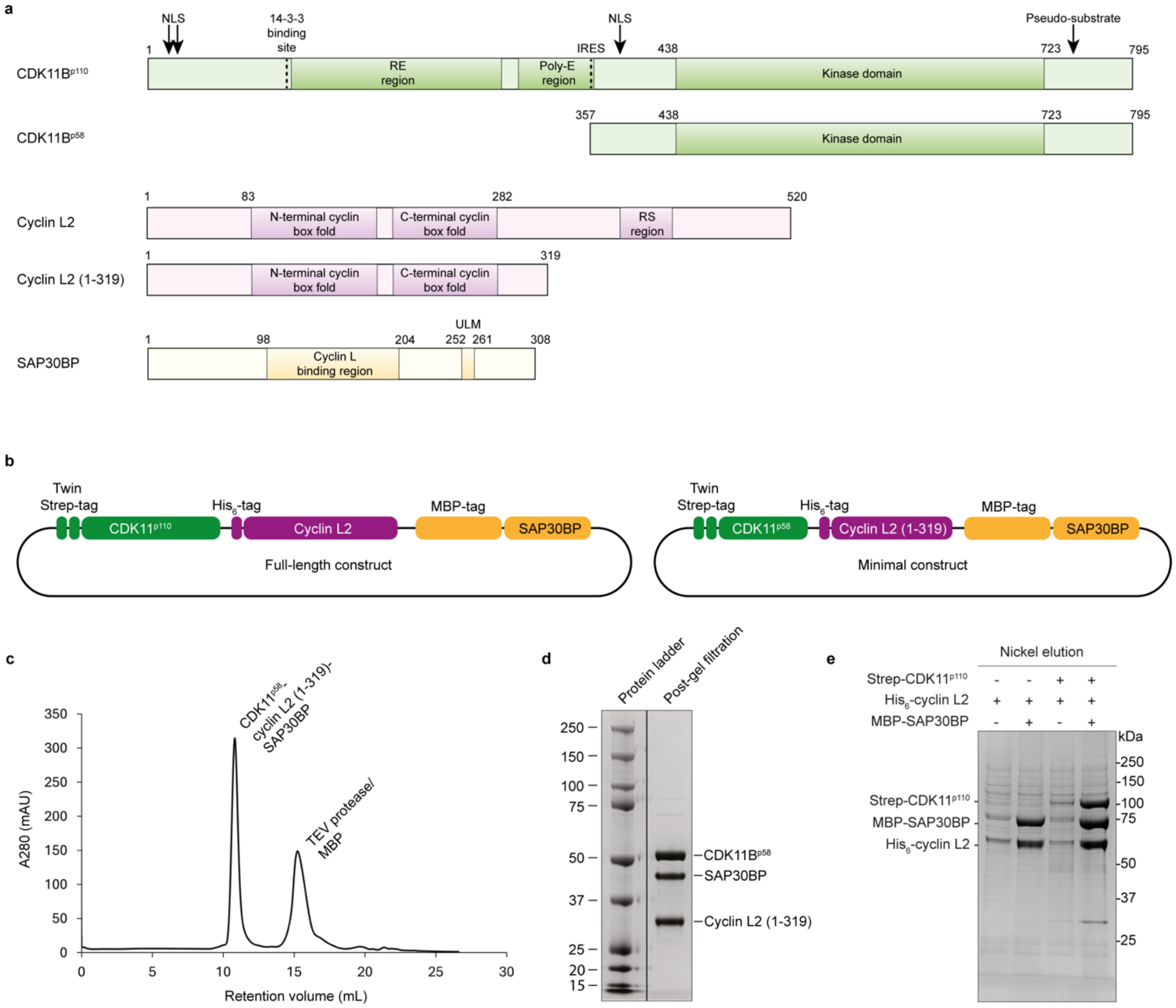

Supplementary Figure 1 | Expression and purification of CDK11-cyclin L-SAP30BP complexes. (**a**) Domain organisation of CDK11B, cyclin L2, and SAP30BP. Protein domains, important binding sites, and notable low-complexity regions are indicated. Abbreviations: NLS, nuclear localisation signal; RE, arginine-glutamate-rich region; RS, arginine-serine-rich region; IRES, internal ribosome entry site; ULM, U2AF-ligand motif. (**b**) Expression constructs for production of full length (CDK11B^{p110}, cyclin L2, SAP30BP) and minimal (CDK11B^{p58}, cyclin L2 (1-319), SAP30BP) CDK11-cyclin L-SAP30BP complexes. (**c**) Size exclusion chromatogram from purification of the minimal construct. (**d**) Purified protein preparation of the minimal construct (used for structure determination). (**e**) Result of small-scale nickel-sepharose pulldown of complexes after co-expression of the indicated subunits in insect cells (representative result; two biological replicates conducted). In the absence of SAP30BP, cyclin L2 recovery is strongly diminished. Source data are provided in a Source Data file.

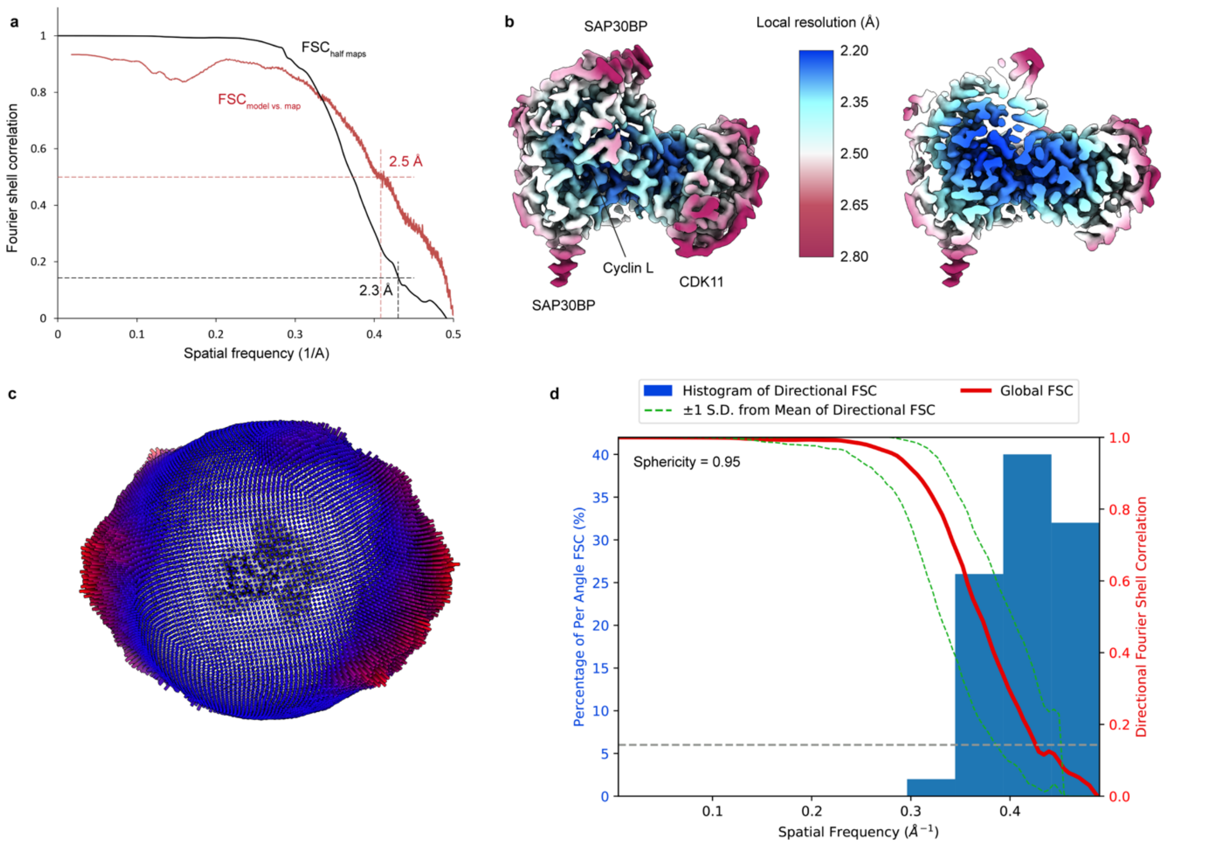

Supplementary Figure 2 | Validation of the CDK11-cyclin L-SAP30BP cryo-EM reconstruction. (**a**) Half-map and model vs. map resolution estimates for the CDK11-cyclin L-SAP30BP structure at FSC = 0.143 and FSC = 0.5, respectively [1]. (**b**) Local resolution estimation for the CDK11-cyclin L-SAP30BP structure. The view of this map is the same as the first views in Fig. 1b, c. (**c**) Orientation distribution plot of the CDK11-cyclin L-SAP30BP cryo-EM reconstruction. (**d**) Analysis of the CDK11-cyclin L-SAP30BP cryo-EM reconstructions by 3D FSC [2]. Source data are provided in a Source Data file.

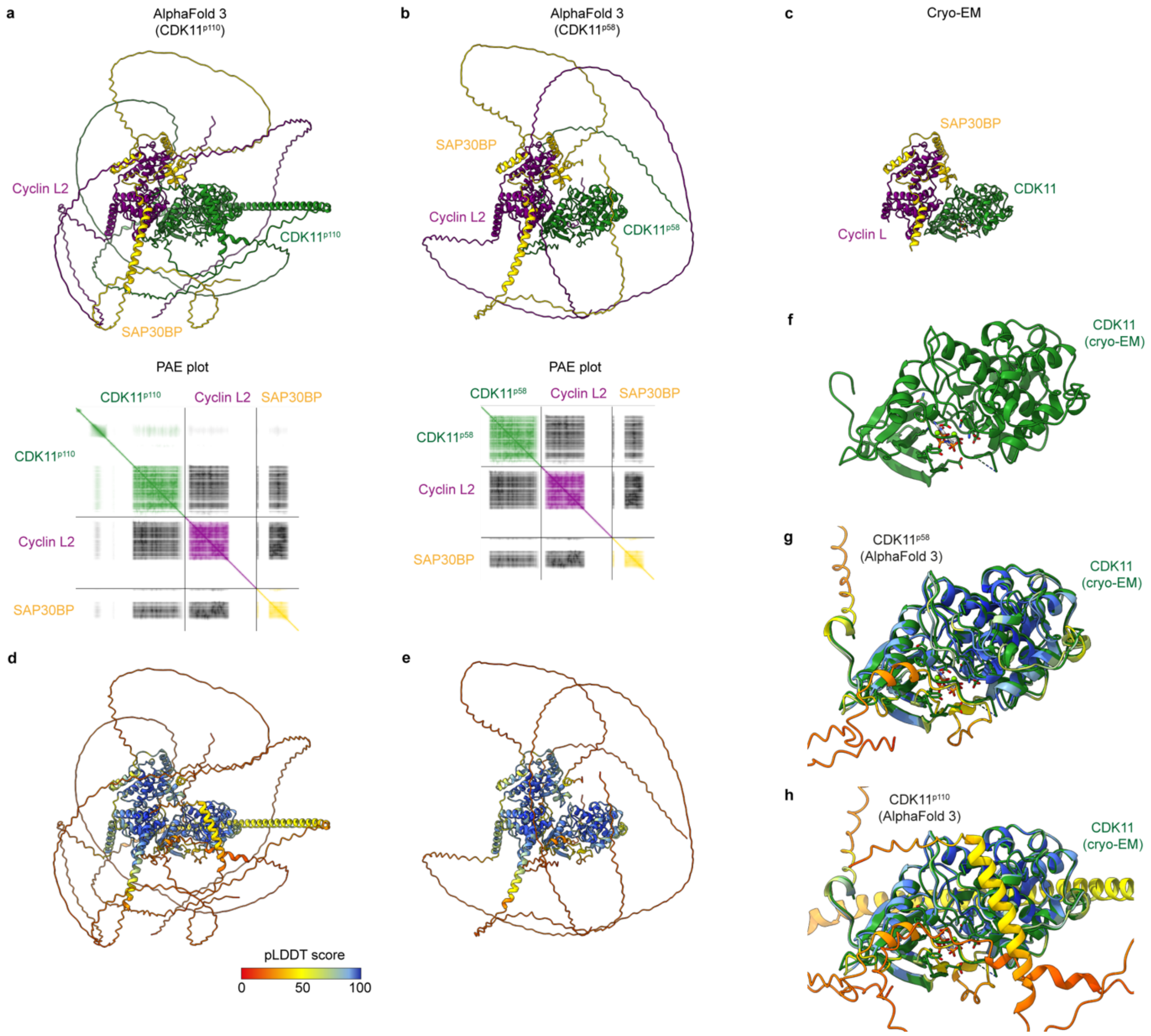

Supplementary Figure 3 | CDK11^{p110} and CDK11^{p58} AlphaFold 3 predictions and comparison to the cryo-EM structure. (**a**) Predicted structure of the CDK11B^{p110}-cyclin L2-SAP30BP complex (top) and plot of the predicted aligned error (bottom). (**b**) Predicted structure of the CDK11B^{p58}-cyclin L2-SAP30BP complex (top) and plot of the predicted aligned error (bottom). (**c**) Cryo-EM structure of the CDK11B-cyclin L2-SAP30BP complex. (**d**, **e**) Plots of the predicted local-distance difference test (pLDDT) score for the structure prediction of the CDK11^{p110}- and CDK11^{p58}-containing complexes. Segments not resolved in the cryo-EM structure mostly carry low pLDDT scores and are likely poorly ordered or flexibly attached in solution. (**f**-**h**) Superposition of the CDK11B structure derived from our cryo-EM data (green, f) with the predictions of the CDK11B^{p58} and CDK11B^{p110} structures (g, h, coloured by pLDDT score). The extensions not observed in our cryo-EM structures carry low pLDDT scores and are likely disordered in the free complex. Full structure prediction outputs from the AlphaFold 3 web server are provided in Supplementary Data 2.

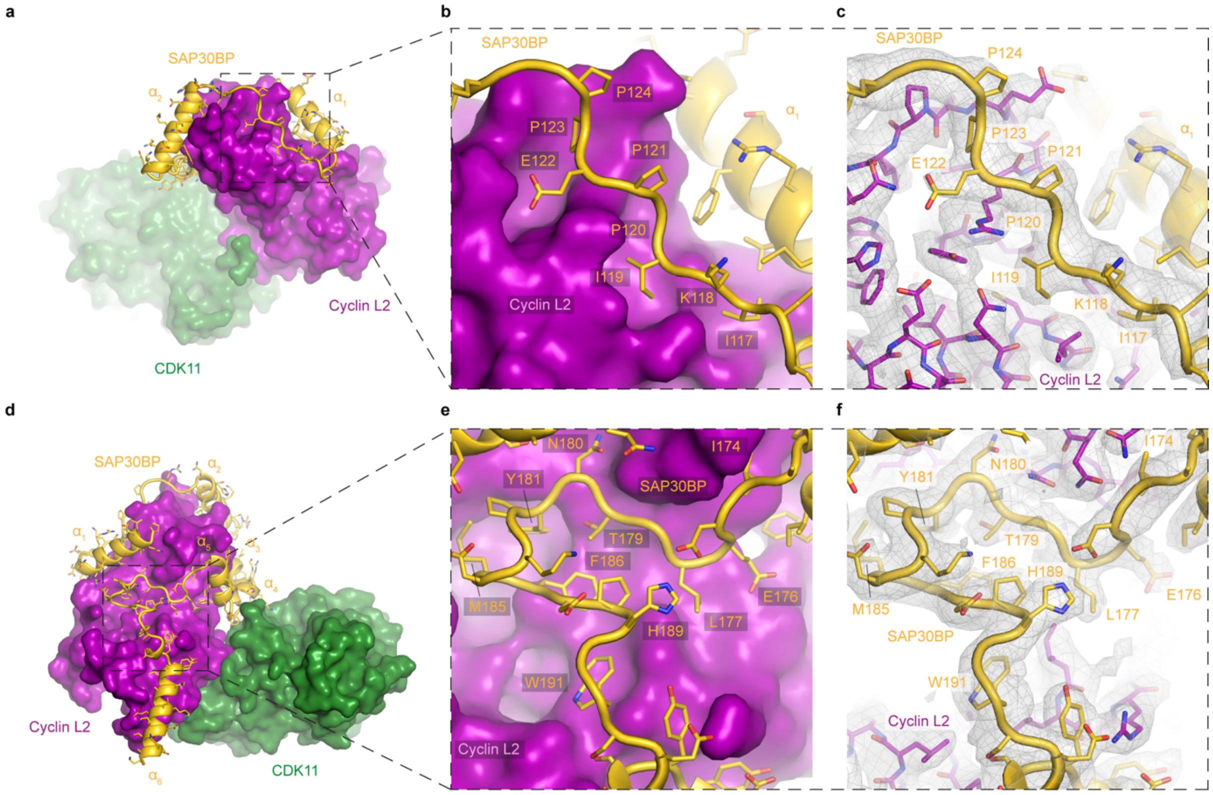

Supplementary Figure 4 | Detailed analysis of the interactions of extended segments in SAP30BP with cyclin L. (**a**-**f**) The interactions of extended segments of SAP30BP with cyclin L are shown in overview (panels a, d), with a surface representation of cyclin L (panels b, e), and with the cryo-EM density (panels c, f).

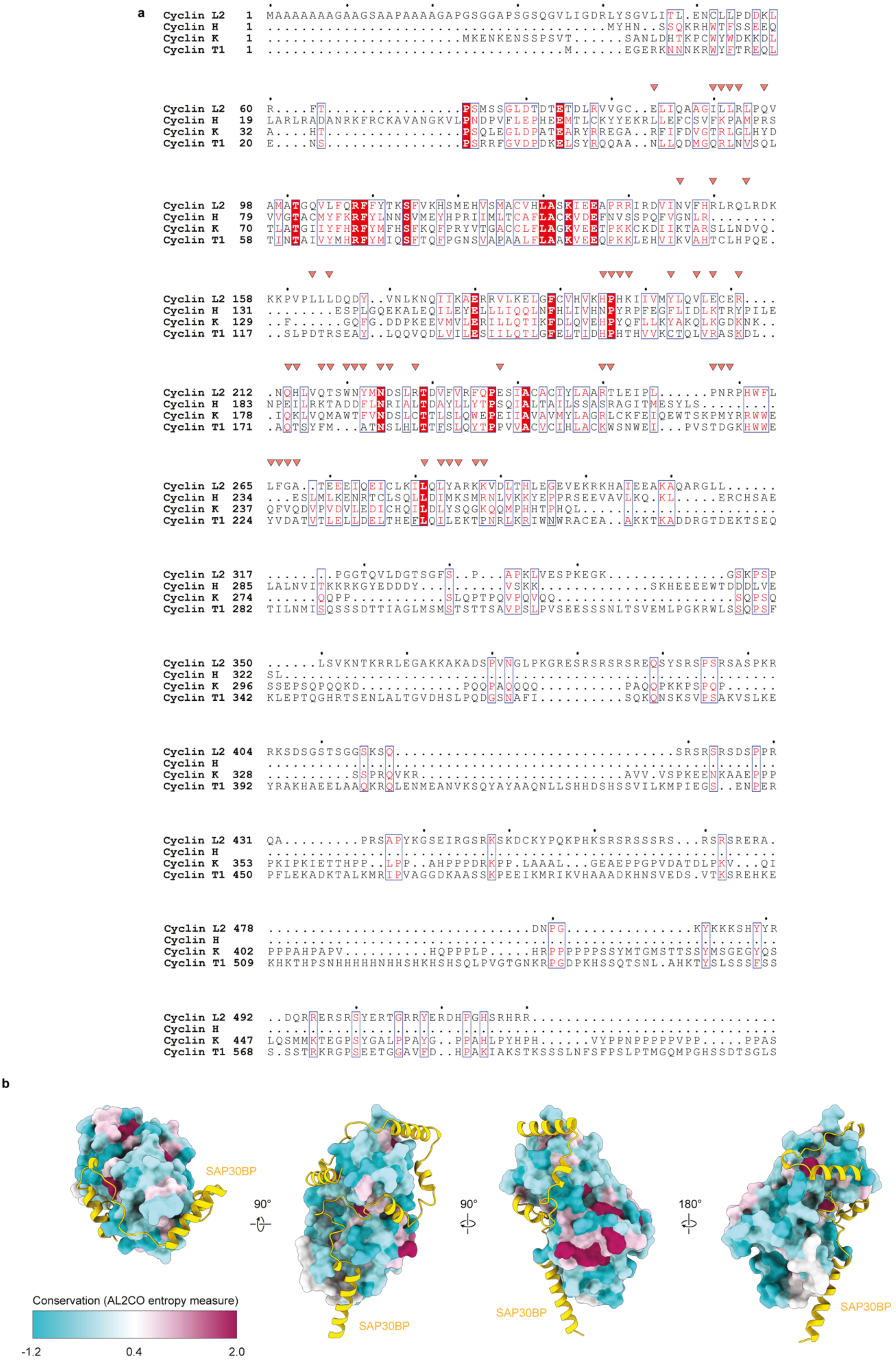

Supplementary Figure 5 | Sequence conservation of SAP30BP binding sites on cyclin L2. (**a**) Multiple sequence alignment of cyclins L2, H, K, and T1. Cyclin L2 residues

interacting with SAP30BP are indicated with orange triangles. (**b**) Conservation of the cyclin sequences shown in panel a mapped onto the structure of cyclin L2 (computed in Chimera X [3]; low conservation is shown in cyan, high conservation in maroon; the darkest red in the structural visualisation corresponds to sequence identity across the four cyclins). SAP30BP is shown in yellow.

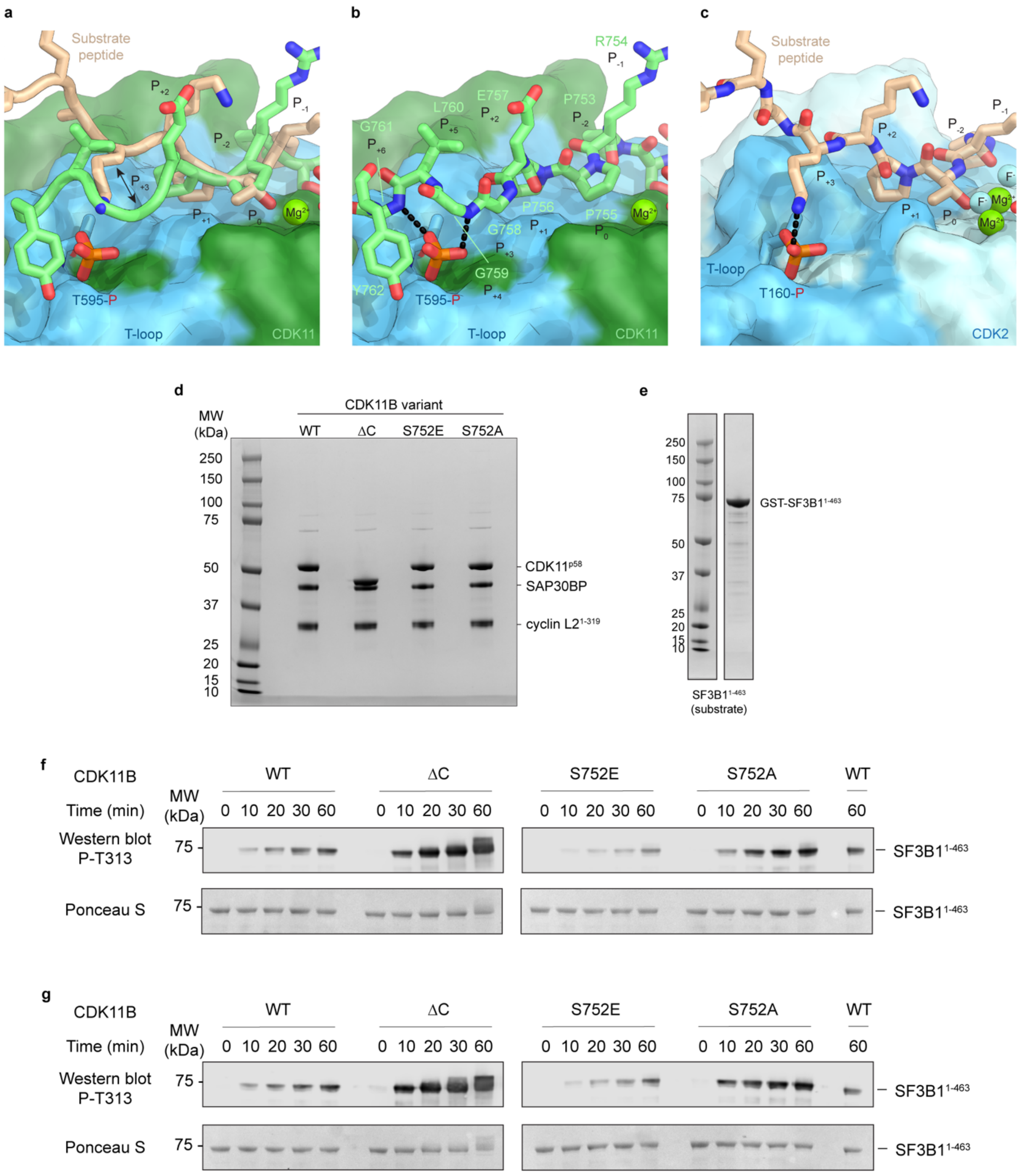

Supplementary Figure 6 | Further structural and biochemical analysis of the CDK11 pseudo-substrate. (**a**) Superposition of a CDK2-cyclin A-bound substrate peptide (brown; PDB ID 3QHR [4]) onto the C-terminal kinase lobe of CDK11 extracted from the CDK11-cyclin L-SAP30BP cryo-EM structure. The CDK11 pseudo-substrate is shown in light green. The paths of the pseudo-substrate and CDK2 substrate diverge at the P_{+3} position. (**b**) Amide protons from CDK11 G579 and G561 are positioned to interact with the phosphate group on T595 within the CDK11 T-loop. (**c**) In the CDK2-cyclin A-substrate complex, the side chain of a lysine residue at position P_{+3} forms an electrostatic interaction with the phosphorylated T160 within the CDK2 T-loop. For clarity of the visualisation, only the C-terminal kinase lobe of CDK2 is shown. (**d**) Coomassie-stained

SDS-PAGE gel with purified complexes containing the mutant variants of CDK11B^{p58} used for kinase assays. This gel serves as sample processing control to verify that calculated dilutions of CDK11 complexes used in kinase assays resulted in equivalent amounts of kinase complex. (**e**) Purified GST-SF3B1$^{1-463}$ assay substrate. (**f**, **g**) Western blots and Ponceau S stained membranes of two additional biological replicates of kinase assays used for statistics in Fig. 4b, c. Source data are provided in a Source Data file.

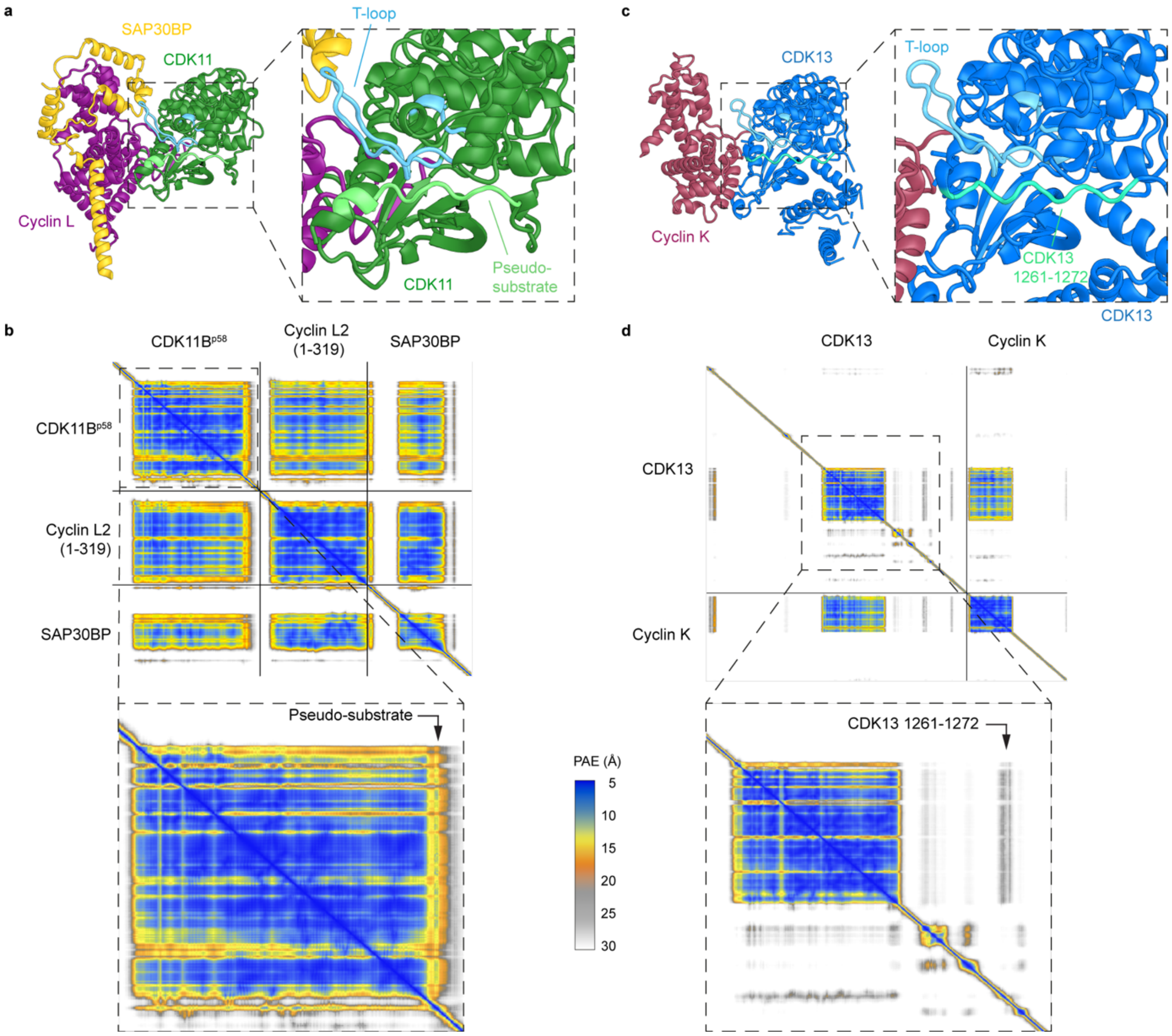

Supplementary Figure 7 | Analysis of AlphaFold 3-predicted structures of human CDK-cyclin complexes to identify possible pseudo-substrates on other CDKs. (**a, b**) The pseudo-substrate in CDK11 (a) is predicted in all five models of one AlphaFold 3 run, and the error according to the PAE plot is comparably low (b). (**c, d**) While low-confidence predictions of disordered CDK or cyclin tails in the area around the active site occur for several CDK-cyclin complexes, CDK13 is the only human CDK for which a CDK segment is predicted to occupy its own substrate binding site in a configuration similar to the CDK11 pseudo-substrate (CDK13 residues 1261-1272, c). However, this interaction is predicted in only one of the five models of one AlphaFold 3 run. A second predicted model places the same segment in proximity of the substrate-binding cleft, but too far away to form the same interactions, and a third model places a different segment of CDK13 in this position. The remaining two models do not place any segment of CDK13 in this area. Accordingly, the PAE plot (d) shows that the error for this prediction is high, indicating that this prediction is unreliable. CDK11 may thus be the only human CDK with a pseudo-substrate. For visualisation in panel c, extended segments predicted with pLDDT < 35 have been removed. The complete set of AlphaFold 3 predictions is provided in Supplementary Data 2.

a

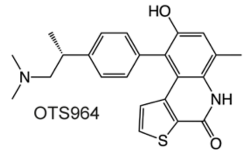

b

CDK11	N (sites)	K_D (nM)	ΔG (kcal/mol)	ΔH (kcal/mol)	ΔS (cal/mol/K)
**Monomer**	0.806	9.8 ± 0.8	-15.9 ± 1.1	-16.3 ± 1.2	-1.52 ± 0.34
**Complex**	0.864	4.3 ± 1.2	-17.4 ± 0.8	-17.9 ± 0.9	-1.83 ± 0.20

c

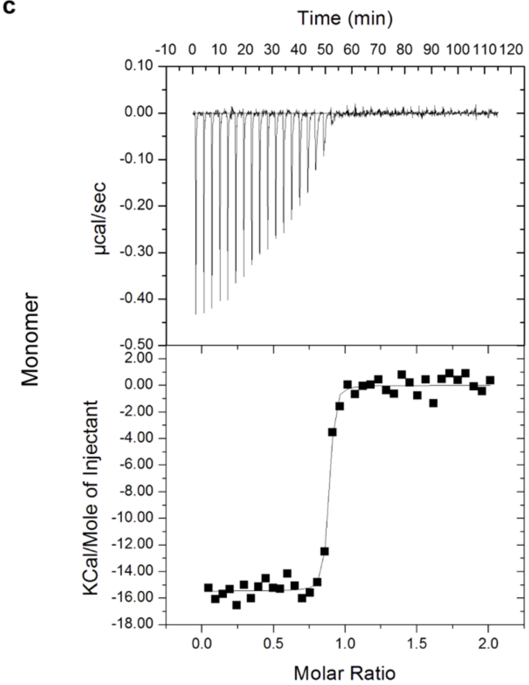

N = 0.863
K_D = 10.3 ± 4.3 nM
ΔH -15460 ± 161.2 cal/mol
ΔS = -15.3 cal/mol/º

d

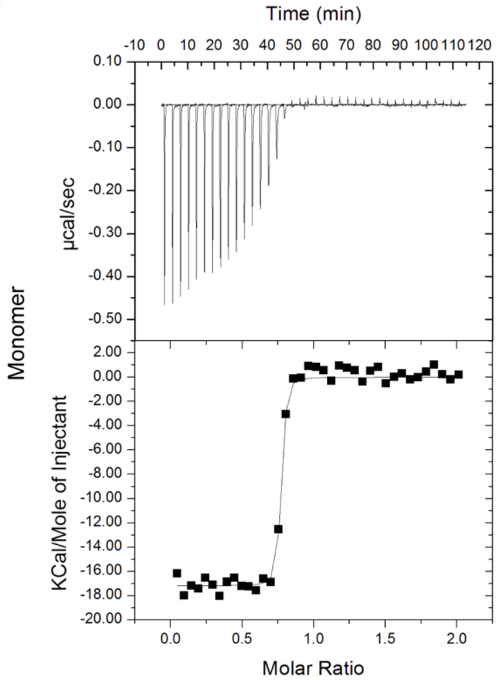

N = 0.749
K_D = 9.3 ± 3.4 nM
ΔH -17210 ± 158.3 cal/mol
ΔS = -21.0 cal/mol/º

e

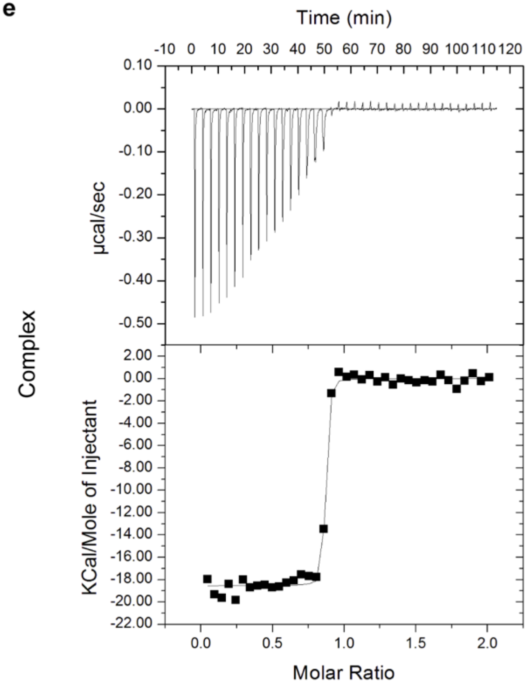

N = 0.848
K_D = 3.5 ± 1.7 nM
ΔH -18550 ± 129.1 cal/mol
ΔS = -23.5 cal/mol/º

f

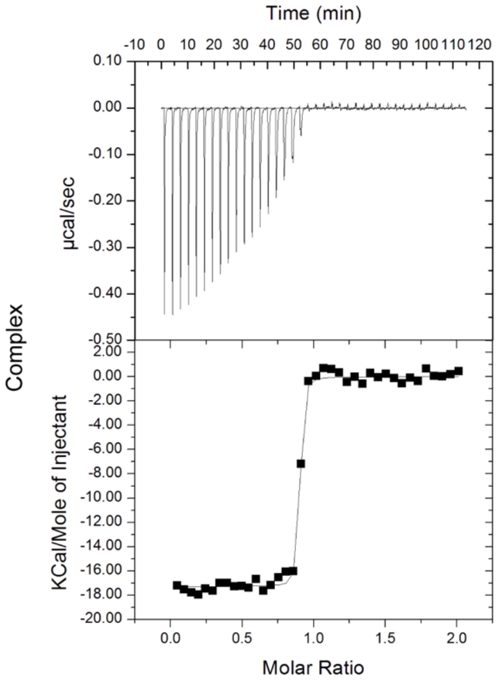

N = 0.880
K_D = 5.1 ± 2.4 nM
ΔH -17300 ± 108.2 cal/mol
ΔS = -20.1 cal/mol/º

Supplementary Figure 8 | Results of ITC analysis of OTS964 affinity to CDK11B^{p58} monomer and CDK11B^{p58}-cyclin L2-SAP30BP complex. (**a**) Depiction of the chemical structure of OTS964. (**b**) Summary of ITC results. Measurements were performed as N = 2 technical replicates each for complex and monomer. (**c**, **d**) ITC traces for titration CDK11 monomer with OTS964. (**e**, **f**) ITC traces for titration of CDK11-cyclin L-SAP30BP complex with OTS964. Source data are provided in a Source Data file.

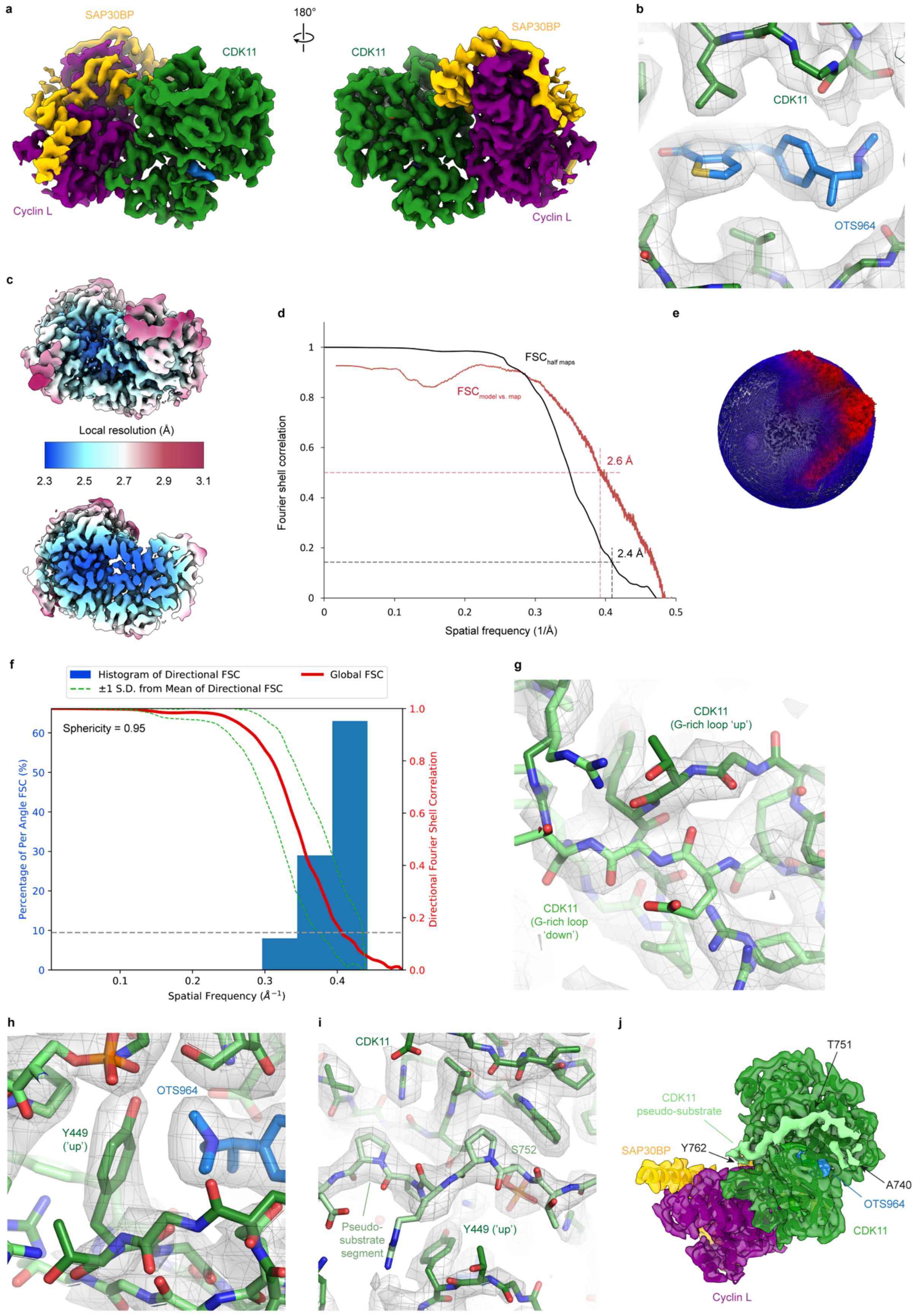

Supplementary Figure 9 | 3D reconstruction and validation of the CDK11-cyclin L-SAP30BP-OTS964 complex. (**a**) Depiction of the CDK11-cyclin L-SAP30BP-OTS964 cryo-

EM structure. CDK11 is shown in green, cyclin L in purple, SAP30BP in yellow, and OTS964 in blue. (**b**) Rendering of OTS964 (blue) in the cryo-EM map of the CDK11-cyclin L-SAP30BP-OTS964 complex (shown as semi-transparent grey mesh and surface). (**c**) Local resolution estimation for the CDK11-cyclin L-SAP30BP-OTS964 structure. (**d**) Half-map and model vs. map resolution estimates for the CDK11-cyclin L-SAP30BP-OTS964 structure at FSC = 0.143 and FSC = 0.5, respectively [1]. Source data are provided in a Source Data file. (**e**) Orientation distribution plot of the CDK11-cyclin L-SAP30BP-OTS964 cryo-EM reconstruction. (**f**) Analysis of the CDK11-cyclin L-SAP30BP-OTS964 cryo-EM reconstruction by 3D FSC [2]. (**g**, **h**) Two conformations of the CDK11 G-rich loop (shown in dark green and lime) with the cryo-EM density shown as semi-transparent grey mesh and surface. The conformations are shown in superposition because we were unable to separate the two G-rich loop conformations computationally at the cryo-EM image processing step. (**i**) The CDK11 pseudo-substrate segment (light green; phosphorylated S752 is labelled) shown in the cryo-EM map of the OTS964-bound CDK11-cyclin L-SAP30BP complex. (**j**) Cryo-EM map of the CDK11-cyclin L-SAP30BP-OTS964 complex (green, purple, yellow, and blue, respectively) post-processed using a b-factor of -10 $Å^2$ and low-pass filtered to 3.3 Å. Additional density of the pseudo-substrate (light green) is visualised in the area between CDK11 A740 and T751.

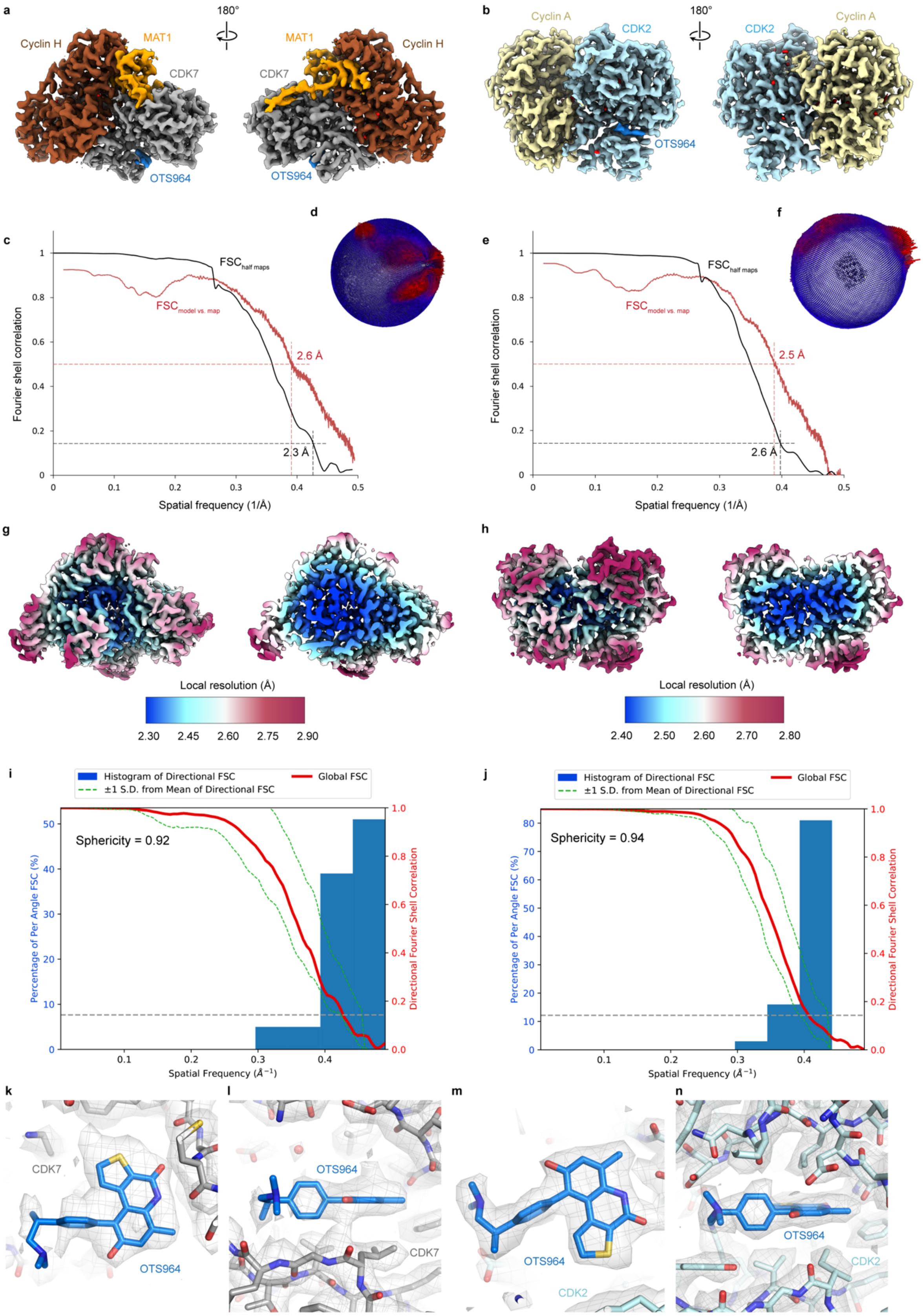

Supplementary Figure 10 | 3D reconstruction and validation of CAK-OTS964 and CDK2-cyclin A-OTS964 complexes. (**a**) Cryo-EM map of CAK-OTS964 at 2.3 Å resolution. CDK7 is shown in grey, MAT1 in orange, cyclin H in brown, and OTS964 in blue. (**b**) Cryo-EM map of CDK2-cyclin A-OTS964 at 2.5 Å resolution. CDK2 is shown in cyan, cyclin A in

light yellow, and OTS964 in blue. (**c**) Half-map and model vs. map resolution estimates for the CAK-OTS964 structure at FSC = 0.143 and FSC = 0.5, respectively [1]. Source data are provided in a Source Data file. (**d**) Orientation distribution of the CAK-OTS064 cryo-EM reconstruction. (**e**, **f**) As c, d, but for CDK2-cyclin A-OTS964. Source data are provided in a Source Data file. (**g**, **h**) Local resolution estimation for the CAK-OTS964 (g) and CDK2-cyclin A-OTS964 (h) cryo-EM reconstructions. (**i**, **j**) Analysis of the CAK-OTS964 (i) and CDK2-cyclin A-OTS964 (j) cryo-EM reconstructions by 3D FSC [2]. (**k**, **l**) Two views of OTS964 (blue) in the cryo-EM map of the CAK-OTS964 complex (CDK7 shown in light grey, cryo-EM density shown as a semi-transparent grey mesh and surface). (**m**, **n**) Two views of OTS964 (blue) in the cryo-EM map of the CDK2-cyclin A-OTS964 complex (CDK7 shown in light cyan, cryo-EM density shown as a semi-transparent grey mesh and surface).

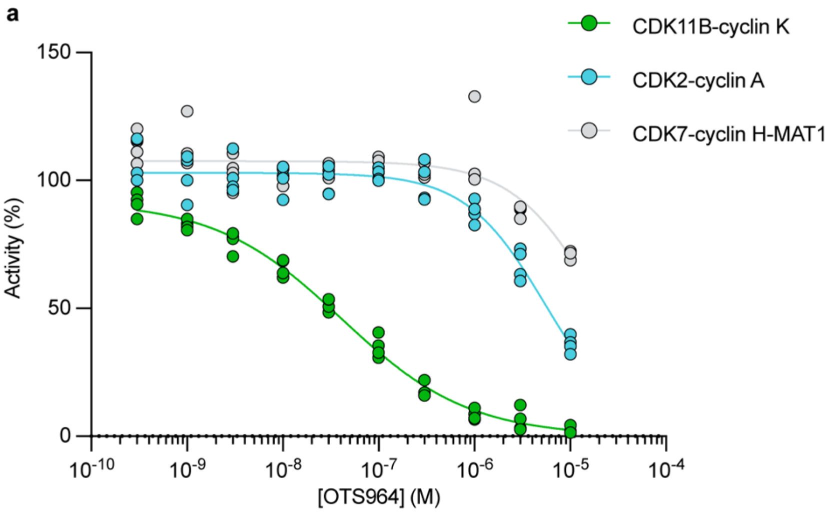

b

Kinase	IC_{50} (nM)
CDK11-cyclin K	41 (30, 54)
CDK2-cyclin A	5,500 (4500, 6700)
CDK7-cyclin H-MAT1	>10,000 (N. D.)

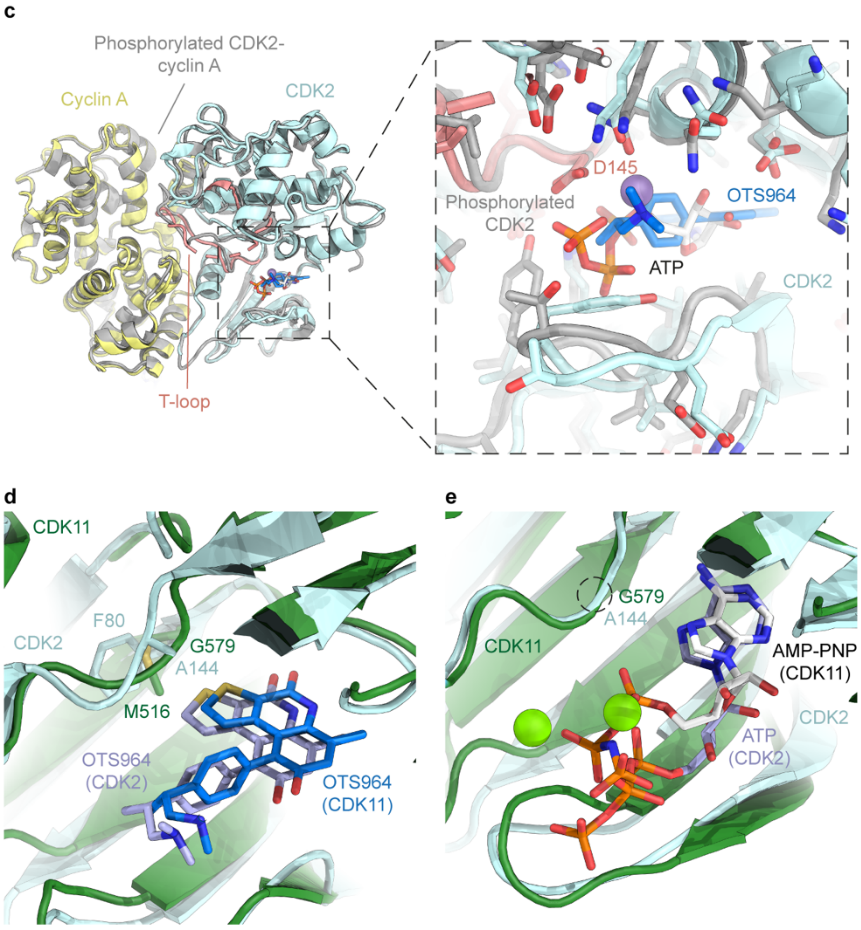

Supplementary Figure 11 | Verification of relative affinity of OTS964 to CDK11-, CDK2-, and CDK7-containing complexes and analysis of inhibitor selectivity. (**a**) Enzyme inhibition assay to determine the inhibitory properties of OTS964 against CDK7-cyclin H-MAT1 and to compare performance of the OTS964 sample used for structural studies against literature data for CDK11B and CDK2 [5,6]. The values for CDK11B-cyclin K obtained are in agreement with literature data on endogenous CDK11B-cyclin L2 complexes [6] and in vitro data [5], thus confirming that OTS964 affinity for CDK2 and CDK7 is indeed much lower than for CDK11-containing complexes. N = 4 data points (technical replicates) were recorded per condition (inhibitor concentration and kinase). Source data are provided in a Source Data file. (**b**) IC_{50} values derived from the data shown in a. Borders of confidence intervals (95%) are shown in brackets. The results indicate that the OTS964 sample used for cryo-EM experiments performs in line with prior data for CDK11B and CDK2 [5]. OTS964 is even more inefficient in inhibiting CDK7 than it is for CDK2. (**c**) Comparison of our cryo-EM structure of CDK2-cyclin A-OTS964 (in colour; CDK2 cyan with T-loop in salmon, cyclin A yellow, OTS964 blue) and the X-ray crystal structure of phosphorylated CDK2-cyclin A [7] (PDB 1JST; grey). The DFG motif at the base of the T-loop is found in the same conformation between the phosphorylated and non-phosphorylated CDK2 structures; structural differences in the N-terminal kinase lobe are likely due to the difference in the bound ligands. (**d**) Comparison between CDK11-cyclin L-SAP30BP-OTS964 and CDK2-cyclin A-OTS964 highlighting the different size of the gatekeeper residues (M516 and F80, respectively) and the structural difference at CDK11 and CDK2 residues G579 and A144, respectively. (**e**) Unlike in the inhibitor-bound state, the backbone conformation around CDK11 G579/CDK2 A144 is almost identical in the nucleotide-bound state of the two kinases (CDK2-cyclin A: PDB ID 1FIN).

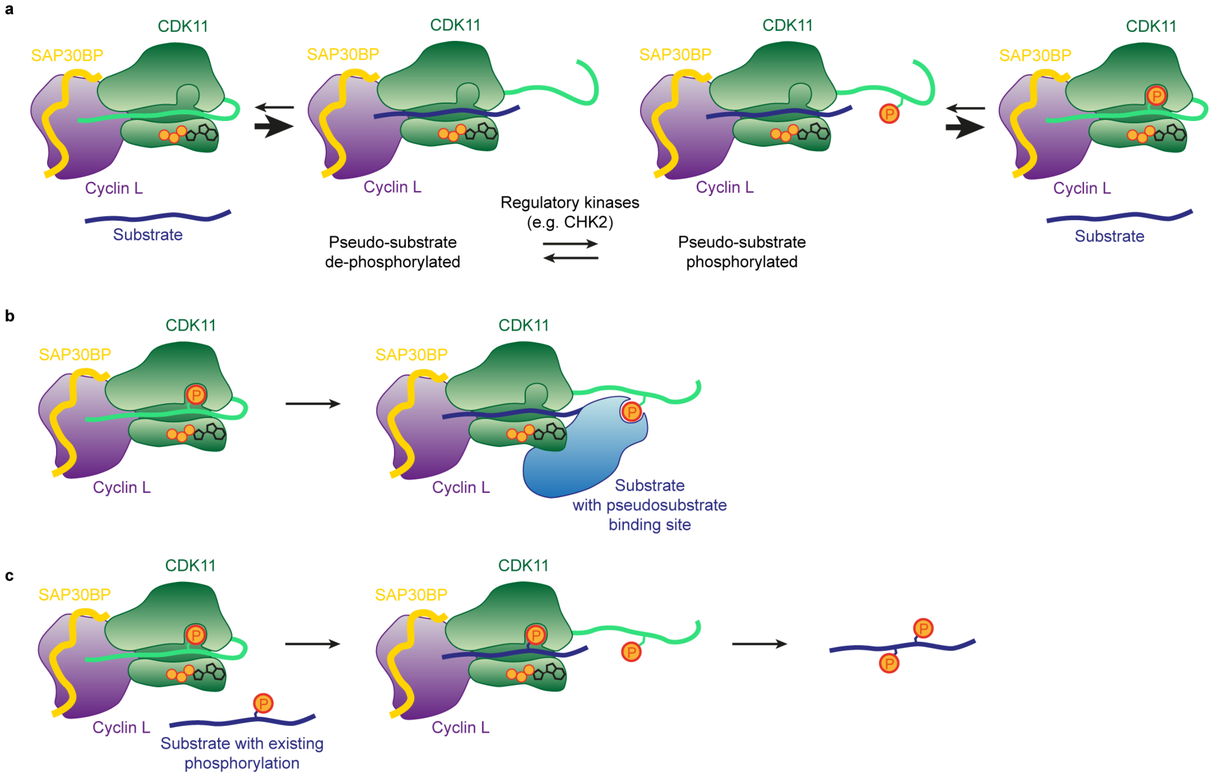

Supplementary Figure 12 | Possible mechanistic consequences of CDK11 pseudo-substrate phosphorylation. (**a**) Schematic illustration of the effect of pseudo-substrate phosphorylation on the efficiency of binding of a simple model substrate to CDK11. The substrate binding site is schematically represented as a cleft, and the active site is indicated by a stylised ATP molecule. (**b**) Schematic representation of a hypothesis that may reconcile our finding of reduced CDK11 activity in the presence of a S752 phosphorylation mimic with the previously documented role of S752 phosphorylation in stimulating CDK11 activity towards certain targets. (**c**) Schematic representation of CDK11 binding of a substrate with pre-existing phosphorylation adjacent to a CDK11 target site.

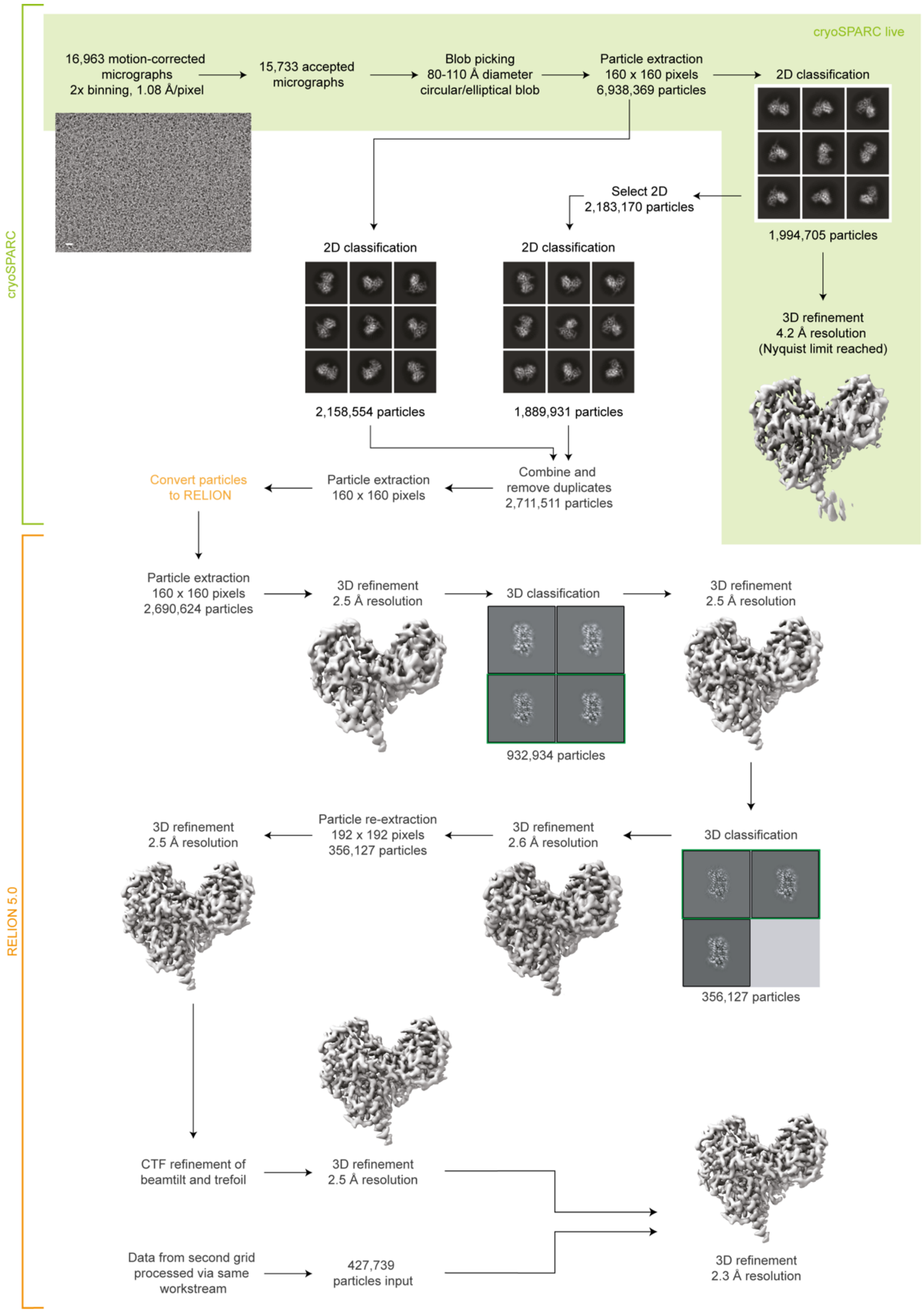

Supplementary Figure 13 | Data processing workflow for structure determination of the CDK11-cyclin L-SAP30BP complex. Data from two grids were processed independently using the outlined workflow. Merging of 356,127 particle images from the first grid and 427,739 particle images from the second grid yielded the final reconstruction at 2.3 Å resolution. Scale bar for sample micrograph: 100 Å.

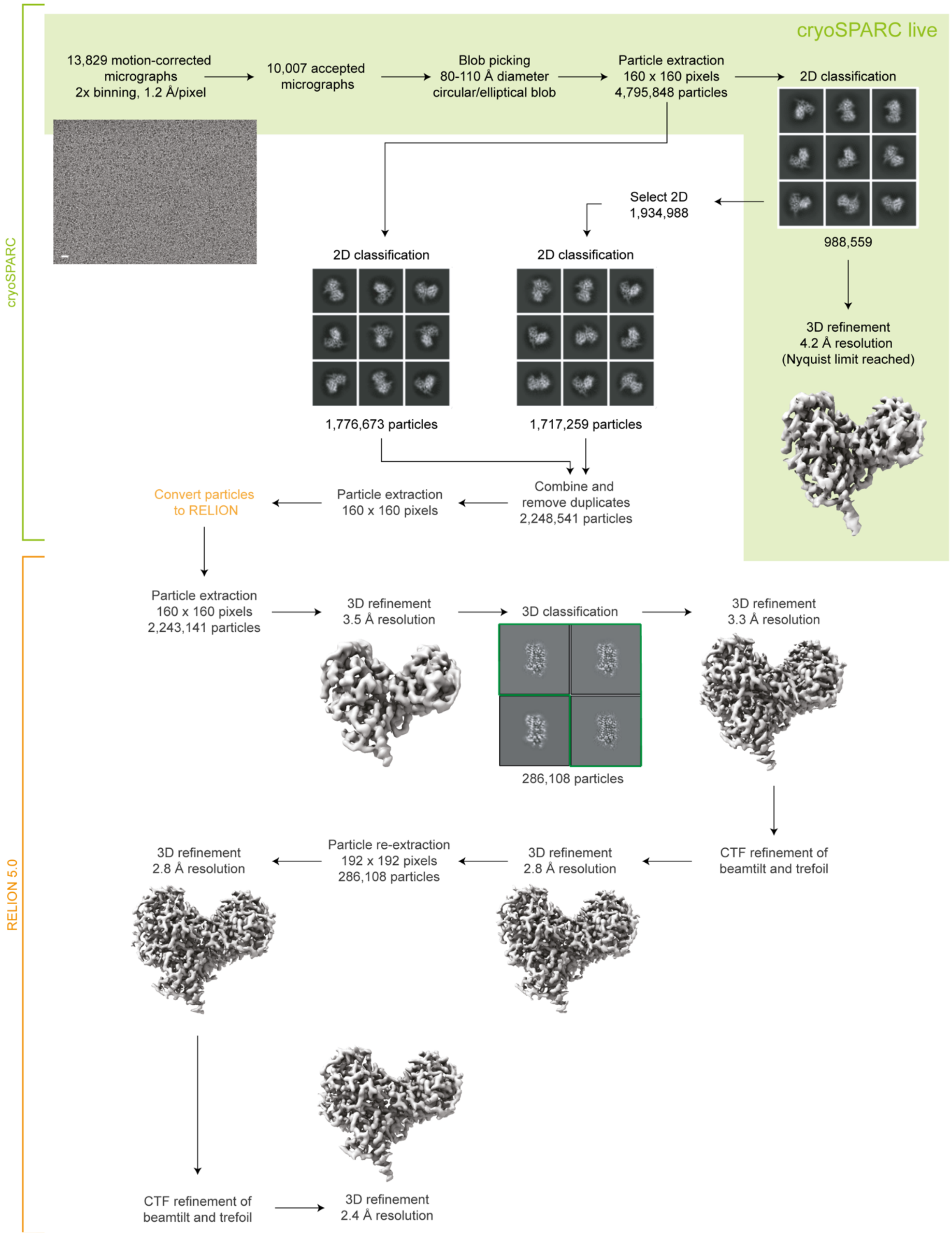

Supplementary Figure 14 | Data processing workflow for structure determination of the CDK11-cyclin L-SAP30BP-OTS964 complex. Scale bar for sample micrograph: 100 Å. See Methods for details.

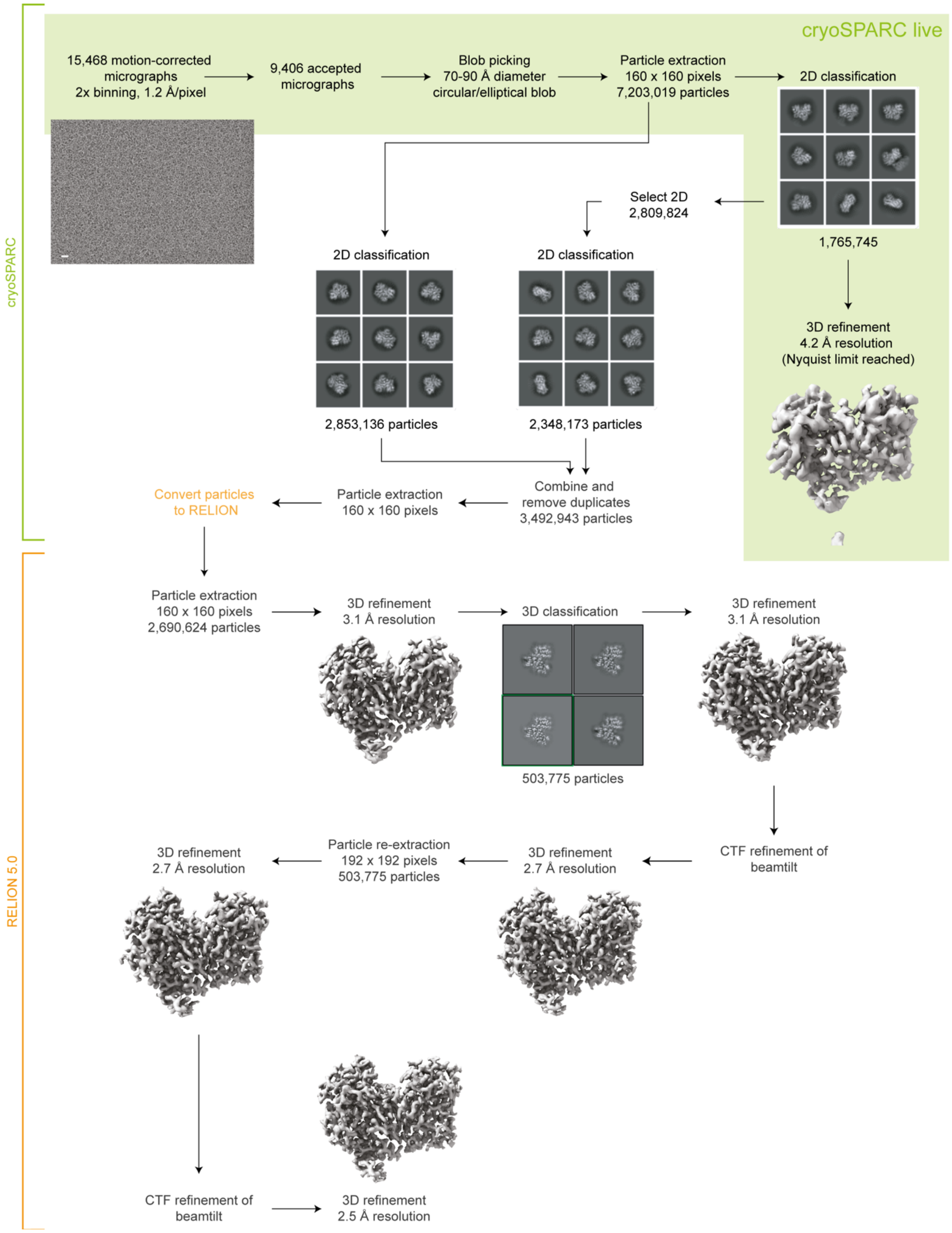

Supplementary Figure 15 | Data processing workflow for structure determination of the CDK2-cyclin A-OTS964 complex. Scale bar for sample micrograph: 100 Å. See Methods for details.

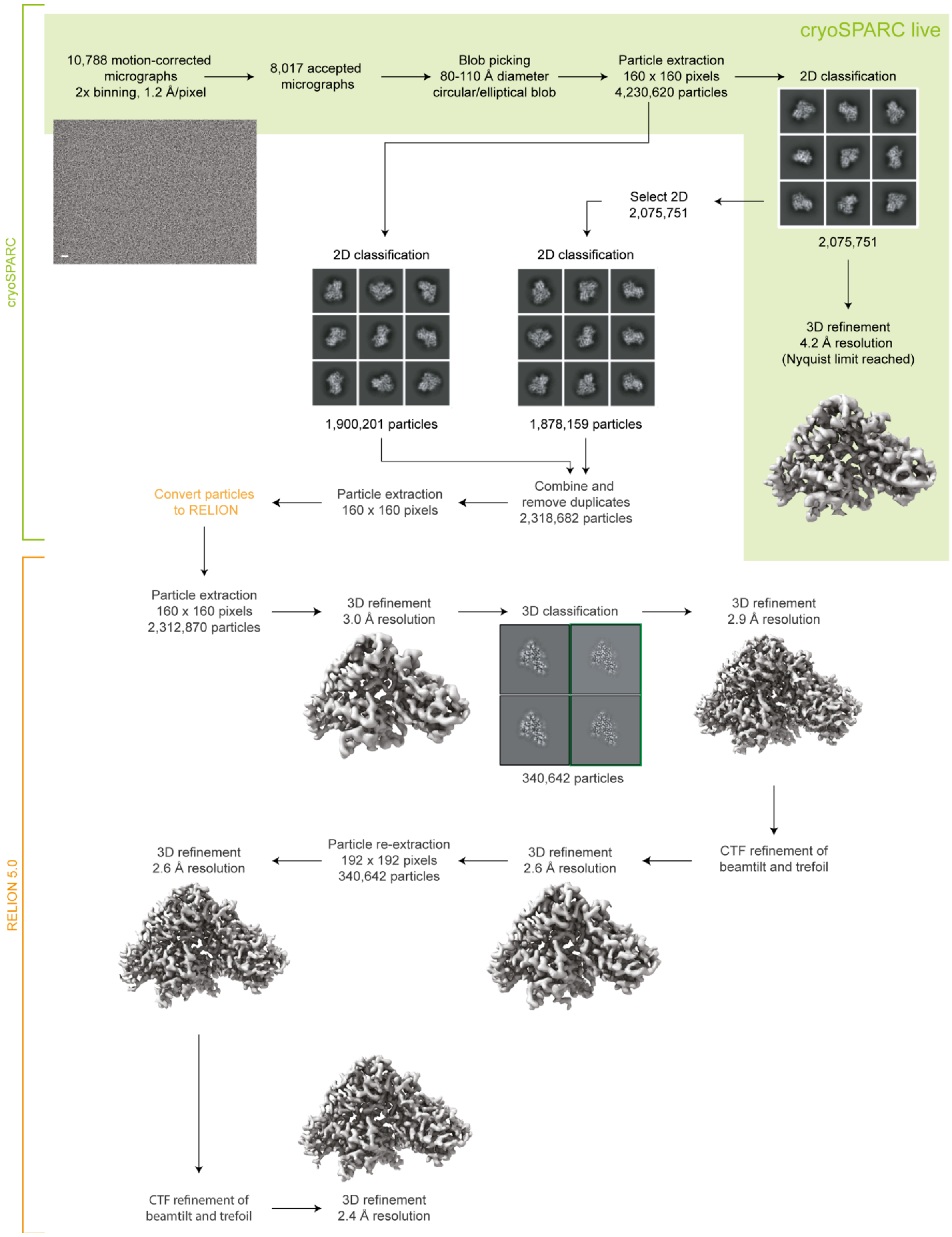

Supplementary Figure 16 | Data processing workflow for structure determination of the CAK-OTS964 complex. Scale bar for sample micrograph: 100 Å. See Methods for details.

Supplementary Table 1 | Summary of mass spectrometry analysis of phosphorylated residues in recombinant CDK11-cyclin L-SAP30BP. Protein: Name of the protein. Only data for CDK11B, cyclin L2, and SAP30BP are shown; detected peptides for low-abundance contaminants are included in Supplementary Dataset 1. Residue: Phosphorylatable residues within detected phosphopeptides. Probability: Probability of a phosphate being located on a residue, given its position in the sequence in case of multiple possible phospho-acceptors in a peptide. Density: Evidence of phosphate in the cryo-EM density. Phosphates observed with high probability in mass spectrometry but not in the cryo-EM map may be present sub-stoichiometrically in our sample.

Protein	Residue	Probability (%)	Density
CDK11B	S384	98.7	Residue not modelled
	T395	97.5	Residue not modelled
	S398	33	Residue not modelled
	Y406	33	Residue not modelled
	S410	100	Residue not modelled
	S414	100	Residue not modelled
	S589	100	Phosphate not observed
	T595	100	Phosphate visualised
	Y615	25	Phosphate not observed
	S616	99.2	Phosphate not observed
	T617	25	Phosphate not observed
	S623	25	Phosphate not observed
	T726	100	Phosphate not observed
	T751	99.6	Phosphate not observed
	S752	100	Phosphate visualised
Cyclin L2	S43	100	Residue not modelled
	T62	100	Residue not modelled
	S239	100	Phosphate not observed
SAP30BP	S9	98.9	Residue not modelled
	Y14	100	Residue not modelled
	S18	100	Residue not modelled
	S22	100	Residue not modelled
	S33	100	Residue not modelled
	S104	100	Phosphate not observed
	S106	99.7	Phosphate not observed
	S113	100	Phosphate not observed
	S255	99.1	Residue not modelled
	S259	33.2	Residue not modelled
	T264	33.2	Residue not modelled
	T265	33.2	Residue not modelled

Supplementary Table 2 | Data collection and refinement statistics, part 1.

**Dataset (kinase / ligand)**	**CDK11-cyclin L-SAP30BP / AMP-PNP**	**CDK11-cyclin L-SAP30BP / OTS964**	
Microscope	Titan Krios G2	Titan Krios G3i	
Stage type	Autoloader	Autoloader	
Voltage (kV)	300	300	
Detector	Gatan K3	Gatan K3	
Energy filter	Bio Quantum	Bio Quantum	
Acquisition mode	2x hardware binning	2x hardware binning	
Pixel size (non-superresolution) (Å)	0.504	0.51	
Defocus range (μm)	0.8-1.8	0.8-1.8	
Electron exposure (e^-/A^2)	70	70	
**Reconstruction**	**EMD-53224**	**EMD-53221**	
Software	RELION 5.0 beta	RELION 5.0 beta	
Particles after 2D classification	3,610,927 + 2,690,624	2,243,141	
Particles final	783,863	286,108	
Extraction box size (pixels)	192 x 192 x 192	192 x 192 x 192	
Final pixel size (Å)	1.008	1.02	
Accuracy rotations (°)	1.36	1.26	
Accuracy translations (Å)	0.42	0.40	
Map resolution (Å)	2.3	2.4	
Map resolution range (Å)	2.2-3.4	2.3-3.5	
Sphericity	0.95	0.95	
Map sharpening B-factor ($Å^2$)	-61.5	-53.7	
**Coordinate refinement**			
Software and algorithm	PHENIX (real space refine)	PHENIX (real space refine)	
Resolution cutoff (Å)	2.3	2.4	
$FSC_{model\text{-}vs\text{-}map}$=0.5 (Å)	2.5	2.6	
**Model**	**PDB-9QKZ**	**PDB-9QKT**	**PDB-9QL1**
Number of residues	728	762	762
Protein	680	681	681
Ligand (AMP-PNP / OTS964 / Mg^{2+} / H_2O)	1 / 0 / 2 / 45	0 / 1 / 0 / 80	0 / 1 / 0 / 80
B-factors overall	84.1	74.8	74.4
Protein	84.2	75.1	74.7
Ligand (AMP-PNP, OTS964, Mg^{2+} / H_2O)	85.3 / 72.5	53.1 / 66.0	55.7 / 65.8
R.M.S. deviations			
Bond lengths (Å)	0.003	0.04	0.04
Bond angles (°)	0.47	1.39	1.40
**Validation**			
Molprobity score	1.56	1.71	1.64
Molprobity clashscore	7.14	5.98	5.54
Rotamer outliers (%)	1.64	3.28	2.79
C_β deviations (%)	0.0	0.0	0.0
Ramachandran plot			
Favored (%)	98.2	98.7	98.3
Allowed (%)	1.6	1.1	1.5
Outliers (%)	0.2	0.2	0.2
Rama-Z scores			
Whole	2.55	2.64	2.74
Helix	2.42	2.66	2.71
Sheet	-0.60	0.03	0.64
Loop	1.23	0.96	0.99

Supplementary Table 3 | Data collection and refinement statistics, part 2.

**Dataset (kinase / ligand)**	**CDK2-cyclin A / OTS964**	**CAK / OTS964**
Microscope	Titan Krios G3i	Titan Krios G3i
Stage type	Autoloader	Autoloader
Voltage (kV)	300	300
Detector	Gatan K3	Gatan K3
Energy filter	Bio Quantum	Bio Quantum
Acquisition mode	2x hardware binning	2x hardware binning
Pixel size (non-superresolution) (Å)	0.51	0.51
Defocus range (µm)	0.6-1.6	0.6-1.8
Electron exposure (e^-/A^2)	70	70
**Reconstruction**	**EMD-53204**	**EMD-53205**
Software	RELION 5.0 beta	RELION 5.0 beta
Particles after 2D classification	3,482,979	2,312,870
Particles final	503,775	340,642
Extraction box size (pixels)	192 x 192 x 192	192 x 192 x 192
Final pixel size (Å)	1.02	1.02
Accuracy rotations (°)	1.48	1.21
Accuracy translations (Å)	0.42	0.40
Map resolution (Å)	2.5	2.4
Map resolution range (Å)	2.4-3.3	2.2-3.4
Sphericity	0.94	0.92
Map sharpening B-factor ($Å^2$)	-69	-27
**Coordinate refinement**		
Software and algorithm	PHENIX (real space refine)	PHENIX (real space refine)
Resolution cutoff (Å)	2.5	2.4
$FSC_{model\text{-}vs\text{-}map}$=0.5 (Å)	2.6	2.6
**Model**	**PDB-9QJJ**	**PDB-9QJN**
Number of residues	602	773
Protein	546	630
Ligand (AMP-PNP / OTS964 / Mg^{2+} / H_2O)	0 / 1 / 0 / 55	0 / 1 / 0 / 142
B-factors overall	37.0	62.0
Protein	37.2	62.0
Ligand (AMP-PNP, OTS964, Mg^{2+} / H_2O)	23.1 / 30.5	90.0 / 56.6
R.M.S. deviations		
Bond lengths (Å)	0.04	0.04
Bond angles (°)	1.55	1.48
**Validation**		
Molprobity score	1.35	1.32
Molprobity clashscore	4.49	5.39
Rotamer outliers (%)	1.4	1.1
C_β deviations (%)	0.0	0.0
Ramachandran plot		
Favored (%)	99.1	98.4
Allowed (%)	0.9	1.4
Outliers (%)	0.0	0.2
Rama-Z scores		
Whole	1.68	1.73
Helix	1.75	2.39
Sheet	0.42	-0.65
Loop	0.61	-0.29

Supplementary Note 1. Analysis of the structure of CAK-OTS964

The quality of the density of the inhibitor in the CDK7-containing CAK-OTS964 complex cryo-EM map at 2.3 Å overall resolution is lower compared to that in the CDK11-containing target complex. This is particularly pronounced for the dimethylamino-propan-2-yl-phenyl substituent extending from the aromatic core of the compound (Supplementary Fig. 10k, l). This suggests that the inhibitor might exhibit conformational or positional flexibility within the CDK7 active site, rather than being stably bound with high affinity in a single pose as observed in the CDK11 complex. This is in line with the poor ability of OTS964 to inhibit CDK7 (Supplementary Fig. 11a, b). The active sites of CDK7 and CDK11 differ in some of the residues that form contacts to OTS964 in the CDK11 complex, providing a possible explanation for this observation. Most notably, CDK11 residue E445, which interacts with OTS964 in CDK11 (Fig. 6b), is equivalent to G19 in CDK7. The presence of a glycine in this position leads to loss of any inhibitor interactions involving this residue in CDK7. Additionally, CDK7 F91 and D97 are further away from the inhibitor than the equivalent residues M516 and D522 in the CDK11 complex, which may further destabilise binding of OTS964 due to lack of hydrophobic packing and hydrogen bonding interactions, respectively (Fig. 6e). Overall, the poor affinity of OTS964 for CDK7 is explained by the absence of several key interactions due to amino acid differences between CDK7 and CDK11 and lack of shape complementarity of the CDK7 active site.

Supplementary Note 2. Detailed analysis of the structure of CDK2-cyclin A-OTS964

The differences in the inhibitor interactions between the CDK2-cyclin A-OTS964 complex, determined at 2.5 Å resolution, and the CDK11-cyclin L-SAP30BP-OTS964 complex are considerably smaller than those observed for the CDK7-bound inhibitor complex. We note that we used an unphosphorylated CDK2-cyclin A complex for determination of the CDK2-cyclin A-OTS964 structure. A superposition of our OTS964-bound structure and the structure of CDK2-cyclin A with a phosphorylated T-loop (Supplementary Fig. 11c) [7] shows that D145, which forms part of the DFG motif at the base of the CDK2 T-loop, occupies an identical conformation between the two complexes. This indicates that the inhibitor interactions observed in our structure likely also apply to the phosphorylated CDK2-cyclin A complex.

The cryo-EM density for CDK2-bound OTS964 is of similar quality as for the CDK11-bound complex (Supplementary Fig. 10m, n). In the context of CDK2, OTS964 can form hydrogen bonds to the hinge region as well as to CDK2 D86 (equivalent to CDK11 D522, Fig. 6f). CDK2 Y15 is tucked under the dimethylamino-propyl group of OTS964 (Fig. 6f). This conformation has been observed in inhibitor-bound CDK2 complexes before [8], but it is incompatible with nucleotide binding [9]. In this position, Y15 might contribute to inhibitor binding by allowing burial of additional surface area and formation of van-der-Waals contacts. One difference between the CDK2 and CDK11 active sites is that CDK2 carries a glycine in the position of CDK11 E445. The CDK2 G11E mutation has been observed to increase OTS964 affinity for CDK2 [10]. Our structure suggests that the glutamate residue introduced in this CDK2 mutant might be able to form either a hydrogen bond to the exocyclic hydroxy group of OTS964, an electrostatic interaction with the protonated dimethylamino-propyl group of the inhibitor, or hydrophobic contacts with the phenyl and dimethylamino groups of the inhibitor. All these possibilities would explain increased OTS964 binding to the CDK2 G11E mutant. Given that the hydrogen bonding ability of the

exocyclic hydroxy group of OTS964 is likely satisfied by interactions with CDK2 D86, hydrophobic packing to nearby portions of the inhibitor is the most likely of these mechanisms [10].

Further contributions to selectivity may arise from shape complementarity, considering that the inhibitor moves slightly towards the gatekeeper (the slightly more recessed F80 in CDK2 instead of M561 in CDK11) in the CDK2-bound structure (Supplementary Fig. 11d). Finally, the identity of the residue in the position of CDK11 G579, a position in which CDK2 harbours an alanine (A144), is known to affect inhibitor binding. Prior work has shown that the introduction of larger, sterically incompatible residues, such as serine in CDK11 G579S, leads to strong resistance to OTS964 (refs [5,6,10]). Our structures show that the backbone conformation at this position differs between OTS964-bound CDK11 and CDK2 (Supplementary Fig. 11d). Due to this conformational difference, CDK2 A144 does not protrude further towards the inhibitor than CDK11 G579 does (Supplementary Fig. 11d). Unexpectedly, CDK11 G579 and CDK2 A144 in the nucleotide-bound state [9] assume overlapping backbone conformations because of a conformational switch in CDK11 G579 (Supplementary Fig. 11e). It is thus possible that the specific ability of CDK11 G579 to undergo this conformational change in the inhibitor-bound state leads to improved packing or shape complementarity, thereby supporting OTS964 binding. This hypothesis would account for the partial OTS964 resistance of cells harbouring the CDK11 G579A mutation [5,10], which should otherwise be accommodated without steric clashes due to the small size of the alanine side chain [10]. However, prior data showing that the G579A mutation enhances, rather than reduces, OTS964 binding to isolated CDK11 *in vitro* [10] indicate that the cellular environment or the assembly of CDK11 into cyclin-bound complexes may have additional, more subtle, effects on inhibitor binding.

Supplementary References

1 Rosenthal, P. B. & Henderson, R. Optimal determination of particle orientation, absolute hand, and contrast loss in single-particle electron cryomicroscopy. *J. Mol. Biol.* **333**, 721-745 (2003). https://doi.org/10.1016/j.jmb.2003.07.013

2 Tan, Y. Z. *et al.* Addressing preferred specimen orientation in single-particle cryo-EM through tilting. *Nat. Meth.* **14**, 793-796 (2017). https://doi.org/10.1038/nmeth.4347

3 Goddard, T. D. *et al.* UCSF ChimeraX: Meeting modern challenges in visualization and analysis. *Protein Sci.* **27**, 14-25 (2018). https://doi.org/10.1002/pro.3235

4 Bao, Zhao Q., Jacobsen, Douglas M. & Young, Matthew A. Briefly Bound to Activate: Transient Binding of a Second Catalytic Magnesium Activates the Structure and Dynamics of CDK2 Kinase for Catalysis. *Structure* **19**, 675-690 (2011). https://doi.org/10.1016/j.str.2011.02.016

5 Lin, A. *et al.* Off-target toxicity is a common mechanism of action of cancer drugs undergoing clinical trials. *Sci. Transl. Med.* **11**, aaw8412 (2019). https://doi.org/10.1126/scitranslmed.aaw8412

6 Hluchý, M. *et al.* CDK11 regulates pre-mRNA splicing by phosphorylation of SF3B1. *Nature* **609**, 829-834 (2022). https://doi.org/10.1038/s41586-022-05204-z

7 Russo, A. A., Jeffrey, P. D. & Pavletich, N. P. Structural basis of cyclin-dependent kinase activation by phosphorylation. *Nat. Struct. Mol. Biol.* **3**, 696-700 (1996). https://doi.org/10.1038/nsb0896-696

8 Wood, D. J. *et al.* Differences in the Conformational Energy Landscape of CDK1 and CDK2 Suggest a Mechanism for Achieving Selective CDK Inhibition. *Cell Chem. Biol.* **26**, 121-130.e125 (2019). https://doi.org/10.1016/j.chembiol.2018.10.015

9 Jeffrey, P. D. *et al.* Mechanism of CDK activation revealed by the structure of a cyclin A-CDK2 complex. *Nature* **376**, 313-320 (1995). https://doi.org/10.1038/376313a0

10 Kelso, S., O'Brien, S., Kurinov, I., Angers, S. & Sicheri, F. Crystal structure of the CDK11 kinase domain bound to the small-molecule inhibitor OTS964. *Structure* **30**, 1615-1625.e1614 (2022). https://doi.org/10.1016/j.str.2022.10.003
