# Supplementary material for: Cryo-EM structures of the CDK11-cyclin L-SAP30BP complex reveal mechanisms of CDK11 regulation
Source: Nat Commun. 2026 Apr 25;17:5718. doi: 10.1038/s41467-026-72329-4 (PMC13324802; doi:10.1038/s41467-026-72329-4)
Supplement: Supplementary file 3 — Description of Additional Supplementary Files [file 41467_2026_72329_MOESM3_ESM.pdf]

Description of additional Supplementary Files

File name: Supplementary Data 1

Description: Detailed results of phospho-proteomics analysis of CDK11-cyclin L-SAP30BP complexes.

File name: Supplementary Data 2

Description: Full set of Alphafold 3 predictions used in this study.

File name: Supplementary Data 3

Description: List of synthetic DNA sequences (oligonucleotides, synthetic genes) used in this study.
