# Supplementary material for: Cryo-EM structures of the CDK11-cyclin L-SAP30BP complex reveal mechanisms of CDK11 regulation
Source: Nat Commun. 2026 Apr 25;17:5718. doi: 10.1038/s41467-026-72329-4 (PMC13324802; doi:10.1038/s41467-026-72329-4)
Supplement: Supplementary file 7 — Reporting Summary [file 41467_2026_72329_MOESM7_ESM.pdf]

nature portfolio

Corresponding author(s): Basil J. Greber

Last updated by author(s): Apr 11, 2026

Reporting Summary

Nature Portfolio wishes to improve the reproducibility of the work that we publish. This form provides structure for consistency and transparency in reporting. For further information on Nature Portfolio policies, see our Editorial Policies and the Editorial Policy Checklist.

Statistics

For all statistical analyses, confirm that the following items are present in the figure legend, table legend, main text, or Methods section.

n/a	Confirmed	
☐	☒	The exact sample size (*n*) for each experimental group/condition, given as a discrete number and unit of measurement
☐	☒	A statement on whether measurements were taken from distinct samples or whether the same sample was measured repeatedly
☐	☒	The statistical test(s) used AND whether they are one- or two-sided<br>*Only common tests should be described solely by name; describe more complex techniques in the Methods section.*
☒	☐	A description of all covariates tested
☐	☒	A description of any assumptions or corrections, such as tests of normality and adjustment for multiple comparisons
☐	☒	A full description of the statistical parameters including central tendency (e.g. means) or other basic estimates (e.g. regression coefficient) AND variation (e.g. standard deviation) or associated estimates of uncertainty (e.g. confidence intervals)
☐	☒	For null hypothesis testing, the test statistic (e.g. *F*, *t*, *r*) with confidence intervals, effect sizes, degrees of freedom and *P* value noted<br>*Give P values as exact values whenever suitable.*
☒	☐	For Bayesian analysis, information on the choice of priors and Markov chain Monte Carlo settings
☒	☐	For hierarchical and complex designs, identification of the appropriate level for tests and full reporting of outcomes
☒	☐	Estimates of effect sizes (e.g. Cohen's *d*, Pearson's *r*), indicating how they were calculated

Our web collection on statistics for biologists contains articles on many of the points above.

Software and code

Policy information about availability of computer code

Data collection	ITC: MicroCal ITC200 (version 1.24.2)<br>Gel imaging: BioRad Image Lab Touch (version 3.0.1.14)<br>Western blot imaging: LICORBio Image Studio (version 5.2.5)<br>Cryo-EM: Thermo Fisher Scientific EPU and EPU multi-grid (versions 3.4 and 3.7), Sherpa (version 1); Gatan Digital Micrograph (version 3.53.41360).<br>Mass spectrometry: Thermo Scientific Xcalibur (version 4.7.69.37), Orbitrap Ascend Tune Application (version 4.2.4321)
Data analysis	Cryo-EM data were analysed using cryoSPARC (versions 3.3.1-4.4.1) and RELION (version 4.0-beta and 5.0-beta).<br>Atomic models were built in COOT version 0.9.6.<br>Atomic coordinates were refined using PHENIX (versions 1.20, 1.21).<br>Map and model visualisation, interpretation, and preparation of figures were performed in UCSF ChimeraX (versions 1.6-1.9) and PyMOL (versions 2.5-3.0).<br>ITC data were analysed using Origin 7 (v7.0552).<br>Mass spectrometry data were analysed using Thermo Fisher Proteome Discoverer (version 3.0.1.27)<br>Western blot band intensities were quantified using ImageJ 1.54g<br>Data from enzymatic activity assays were plotted and analyzed using GraphPad Prism versions 10.3 and 10.6.1.

For manuscripts utilizing custom algorithms or software that are central to the research but not yet described in published literature, software must be made available to editors and reviewers. We strongly encourage code deposition in a community repository (e.g. GitHub). See the Nature Portfolio guidelines for submitting code & software for further information.

Data

Policy information about availability of data

All manuscripts must include a data availability statement. This statement should provide the following information, where applicable:

- Accession codes, unique identifiers, or web links for publicly available datasets
- A description of any restrictions on data availability
- For clinical datasets or third party data, please ensure that the statement adheres to our policy

The cryo-EM maps and atomic coordinate models for CDK11-cyclin L-SAP30BP-AMPNP, CDK11-cyclin L-SAP30BP-OTS964 (1 EMDB and 2 PDB entries), CAK-OTS964, and CDK2-cyclin A-OTS964 structures have been deposited in the EMDB with accession codes EMD-53224 [https://www.ebi.ac.uk/emdb/EMD-53224], EMD-53221 [https://www.ebi.ac.uk/emdb/EMD-53221], EMD-53205 [https://www.ebi.ac.uk/emdb/EMD-53205], and EMD-53204 [https://www.ebi.ac.uk/emdb/EMD-53204] and in the PDB with accession codes 9QKZ [https://doi.org/10.2210/pdb9QKZ/pdb], 9QKT [https://doi.org/10.2210/pdb9QKT/pdb], 9QL1 [https://doi.org/10.2210/pdb9QL1/pdb], 9QJN [https://doi.org/10.2210/pdb9QJN/pdb], and 9QJJ [https://doi.org/10.2210/pdb9QJJ/pdb]. Maps with reduced sharpening B-factor (B = -10) used in some figures for visualisation of more dynamic protein segments have been supplied as additional maps within the EMDB entries of the CDK11B-cyclin L2-SAP30BP structures. Mass spectrometry data have been deposited to the ProteomeXchange Consortium via the PRIDE partner repository with accession code PXD060582 [http://proteomecentral.proteomexchange.org/cgi/GetDataset?ID=PXD060582]. Uncropped Western blot membranes, uncropped SDS-PAGE gels, and data points used for line graphs and bar charts are provided as Source Data.

Research involving human participants, their data, or biological material

Policy information about studies with human participants or human data. See also policy information about sex, gender (identity/presentation), and sexual orientation and race, ethnicity and racism.

Reporting on sex and gender	Our research did not involve human participants or animals.
Reporting on race, ethnicity, or other socially relevant groupings	Our research did not involve human participants or animals.
Population characteristics	Our research did not involve human participants or animals.
Recruitment	Our research did not involve human participants or animals.
Ethics oversight	Our research did not involve human participants or animals.

Note that full information on the approval of the study protocol must also be provided in the manuscript.

Field-specific reporting

Please select the one below that is the best fit for your research. If you are not sure, read the appropriate sections before making your selection.

☒ Life sciences ☐ Behavioural & social sciences ☐ Ecological, evolutionary & environmental sciences

For a reference copy of the document with all sections, see nature.com/documents/nr-reporting-summary-flat.pdf

Life sciences study design

All studies must disclose on these points even when the disclosure is negative.

Sample size	Dataset sizes of cryo-EM experiments were determined according to workflow requirements, instrument availability, and reconstruction resolution targets (approximately 2-3 Å resolution).<br><br>Sample size for in vitro kinase assays was chosen as N = 3, a sample size that is broadly accepted by the scientific community for this type of assay and allows statistical analysis.
Data exclusions	Data exclusion during cryo-EM data collection and data processing was performed according to established standards in the field. Poor quality micrographs were excluded based on contamination with crystalline ice, high specimen motion leading to poor CTF fit estimates, and ice thickness. Cryo-EM particle images were classified using two- and three-dimensional classification algorithms to remove poor-quality particles.
Replication	During cryo-EM sample screening, we collected data from more than one (typically two) grids per specimen with consistent results between grids, except for quality differences that can be explained by the stochasticity of the grid preparation process.<br><br>Biochemical experiments (e.g. pull-downs) were repeated at least once to ensure reproducibility of the results (2 biological or technical replicates, as described in the figure legends). Kinase assays used N = 3 biological replicates as described above. ITC experiments used 2 technical replicates. Experiments were reproducible (i.e. replicates are consistent with each other).
Randomization	Allocation of particle images into cryo-EM particle half-sets for Gold standard refinement was randomized by the data processing software (RELION).<br><br>Other than cryo-EM image processing, the nature of this study does not require randomization because it is an in vitro biochemistry/structural

	biology study that does not involve any animals, human samples, or treatment allocations, and no data in the manuscript (other than cryo-EM particles) required random allocation to different experimental groups.
Blinding	None of the data in the manuscript required blinding of the experimenter to avoid bias. Blinding is not an established practice in in vitro biochemistry/structural biology studies of this kind.

Reporting for specific materials, systems and methods

We require information from authors about some types of materials, experimental systems and methods used in many studies. Here, indicate whether each material, system or method listed is relevant to your study. If you are not sure if a list item applies to your research, read the appropriate section before selecting a response.

Materials & experimental systems

n/a	Involved in the study	
☐	☒	Antibodies
☐	☒	Eukaryotic cell lines
☒	☐	Palaeontology and archaeology
☒	☐	Animals and other organisms
☒	☐	Clinical data
☒	☐	Dual use research of concern
☒	☐	Plants

Methods

n/a	Involved in the study	
☒	☐	ChIP-seq
☒	☐	Flow cytometry
☒	☐	MRI-based neuroimaging

Antibodies

Antibodies used	Primary Antibody:<br>Anti-phospho-SF3B1 (Thr313): Cell Signalling Technologies, cat. #25009, clone D8D8V, lot number 1; dilution: 1:2,000<br><br>Secondary antibody:<br>Goat anti-rabbit IRDye 800CW, LICOR Bio, cat. #926-32211, RRID AB_2651127, lot number D50528-08; dilution: 1:20,000
Validation	All antibodies are obtained from a commercial source, used exclusively in the context of purified proteins and protein complexes (such that bands at the correct molecular weight are indicative of specific detection), and have been validated in the literature.<br><br>The anti-phospho-SF3B1 primary antibody has been used extensively in the literature (12 prior publications; https://www.cellsignal.com/products/primary-antibodies/phospho-sf3b1-thr313-d8d8v-rabbit-monoclonal-antibody/25009), and the secondary antibody has been used in more than 5000 publications (https://shop.licorbio.com/reagents/irdye-800cw-goat-anti-rabbit-igg-secondary-antibody/).

Eukaryotic cell lines

Policy information about cell lines and Sex and Gender in Research

Cell line source(s)	Insect cells: Our study used commercially available Spodoptera frugiperda Sf9 and Trichoplusia ni High5 insect cell lines purchased from Thermo Fisher (catalogue numbers 11496015 and B85502, respectively).
Authentication	Not authenticated (standard cell lines obtained from commercial source).
Mycoplasma contamination	Insect cells are being tested regularly by facility staff and have been found to be free of contamination.
Commonly misidentified lines<br>(See ICLAC register)	None.

Plants

Seed stocks	Not applicable to this study.
Novel plant genotypes	Not applicable to this study.
Authentication	Not applicable to this study.
