# Supplementary material for: Cryo-EM structures of the CDK11-cyclin L-SAP30BP complex reveal mechanisms of CDK11 regulation
Source: Nat Commun. 2026 Apr 25;17:5718. doi: 10.1038/s41467-026-72329-4 (PMC13324802; doi:10.1038/s41467-026-72329-4)
Supplement: Supplementary file 2 — Peer Review File [file 41467_2026_72329_MOESM2_ESM.pdf]

nature portfolio

Peer Review File

Cryo-EM structures of the CDK11-cyclin L-SAP30BP complex reveal mechanisms of CDK11 regulation

Corresponding Author: Dr Basil Greber

This file contains all reviewer reports in order by version, followed by all author rebuttals in order by version.

Version 0:

Reviewer comments:

Reviewer #1

(Remarks to the Author)
In this paper McGeoch and colleague investigate the molecular mechanism of CDK11 activation using biochemical and structural methods. To this end the authors in vitro reconstitute the CDK11-cyclin L-SAP30BP complex and determine the structure of this complex to high-resolution using cryoEM. In addition the authors determine the structure of this complex bound to the inhibitor OTS964 and compare this structure to a previous crystal structure of this inhibitor bound to the isolated kinase domain of CDK11. Overall this paper is of excellent quality and I enjoyed reading it. The paper is very clearly written, and also the figures are intuitive and informative. The authors did a great job in conveying their findings. In my opinion this paper is of such high quality that justifies a publication in Nature Communications.
I have only a few minor comments to make:
1) “To facilitate structure determination, we truncated the N-terminus of CDK11, generating CDK11p58 (CDK11Bp110 residues 357-795) and removed the C-terminal tail of cyclin L2, including its predicted disordered RS- domain, resulting in expression of cyclin L2 residues 1-319 (Supplementary Fig. 1a, b).”
This description seems vague. Are the authors implying that the unstructured N-terminus of CDK1 and the C-terminal tail of cyclin L2 would preclude particle alignment by contributing to noise or is it that the truncation resulted in more stable and better expressed complexes? I would suggest to modify this sentence.
2) “The T-loop of CDK11 is found in an extended, active conformation, and the features of our cryo-EM map indicate that it is phosphorylated at T595 (CDK11Bp110 residue numbering; Fig. 1c, Supplementary Fig. 2g).”
The discovery that CDK-cyclin complexes are activated when co-expressed in insect cells has been described before (Yu et al., 2021) and (Pim J Huis in't Veld, 2022). Since a similar strategy has been applied for this work it might be appropriate to cite such papers.
3) “These interactions appear to be highly specific to cyclin L, as SAP30BP did not form stable complexes when co-expressed with the phylogenetically related cyclins H, K, and T (Fig. 2e) 32.”
A sequence alignment between the different cyclins L, H, K and T with residues highlighted that are involved in mediating interaction to SAP30BP might support this claim. In my opinion it is possible to observe a weak interaction with cyclin T in the provided gel and I would therefore also slightly rephrase this sentence (e.g. only weak/transient interactions are formed). But this is only meant as a suggestion.
4) “We note that substantial kinase activity is observed in all CDK11 variants tested, indicating that none of the tested configurations of S752 results in complete inhibition, probably because true substrates can outcompete all configurations of the pseudo-substrate, albeit with variable efficiency”
Affinity measurements with several peptides (phosphorylated and non-phosphorylated) that resemble the C-terminal psuedo-substrate tail and CDK11 substrates, e.g. SF3B1 might shed further light on the inhibitory mechanism of the C-terminal tail.
5) “…, results reported in that study indicate that cyclin binding improves OTS964 affinity for CDKs and suggest that the isolated kinase domain might not be able to fully recapitulate the compound-binding properties of the full-length cellular CDK11 protein 29“.
Since Kelso et al. did not perform affinity measurements using a CDK11-cyclin L complex (due to expression difficulties – which are nicely summarised in this study), it would be interesting to compare binding affinities of OTS964 bound to insolated CDK11 with binding of OTS964 to the CDK11-cyclin L-SAP30BP complex.
In brief, this is a very well designed and executed study with interesting findings that warrant publication in Nature Communications. The paper is clearly written and the figures are of the same high quality. Congratulations to the authors. Nevertheless, I think the manuscript would benefit from adding some affinity measurements and small text modifications.

Reviewer #2

(Remarks to the Author)
The study by McGeoch and co-workers presents the high-resolution (2.3 Å) cryo-EM structure of the human CDK11-cyclin L2-SAP30BP complex, which is crucial for the regulation of the spliceosome, e.g. through the phosphorylation of SF3B1. The structure shows that SAP30BP stabilizes cyclin L2 and promotes CDK11 complex assembly by forming extensive interactions with cyclin L2 and modest interactions with the CDK11 T-loop. A pseudo-substrate sequence motif about 30 residues C-terminal to the kinase domain was identified, acting as an auto-regulatory element modulated by phosphorylation. Additionally, the authors determined the structure of the CDK11 complex bound to the clinical inhibitor OTS964, providing insights into its specificity and mechanism of action. Structural comparisons with off-target CDKs CDK2 and CDK7 explain OTS964's selectivity for CDK11. Overall, the study provides a comprehensive framework for understanding CDK11 structure and regulation, and offers strategies for targeted therapeutic development targeting this transcriptional kinase.

This is a very timely and important study on one of the missing pieces in transcriptional kinase regulation. I appreciate the high standard of structural characterization and the comprehensive analysis of compound binding. I have only a couple of comments and suggestion to this study.

Comments:
Introduction, page 3. Please write out the names for kinases PBK (TOPK).

Results and Discussion, page 6, bottom: Does the magnesium coordination involve any of the pseudo-substrate residues? Although the pseudo-substrate motif is not introduced until the following paragraph, it would be nice if the authors could make a preliminary statement as to whether the density of the second magnesium is only due to the presence of the pseudo-substrate density.

Page 7: The G-rich loop of CDK11 reads GTYG (but not GTYGG) and encompasses residues 447-450. More common would be the denomination as EEGTYG (445-450), where CDK11 is one of the few exceptions where the first glycine from the consensus sequence GxGTYG is changed to glutamate.

Page 9: To this reviewers' knowledge, the terminology for cyclins is that they are composed of two cyclin boxes (usually with high structural homology), which are designated as first and second cyclin box, but not N- and C-terminal cyclin domain.

Figure 5: The kinases in panels e and f should be labelled as CDK7 and CDK2, similar to CDK11 in panels b and c.

Figure 6: I consider the CDK11 dimerization model in panel b highly unlikely, but this is also mentioned in the text. Any chances, that the linker region between the C-term of the kinase domain and the pseudo-substrate motif pS752 (in cis-assembly) could be resolved in the density map upon different filtering/masking?

Supplementary Fig. 1: The pseudo-substrate motif at position 752 should be indicated in the bar diagram of panel a.

Supplementary Fig. 7: I suppose, it should read CDK11B-cyclin L in panel a.

Reviewer #3

(Remarks to the Author)
In this manuscript McGeoch et al. present the structure of the CDK11-cyclinL-SAP30BP complex. CDK11-cyclin L has proved difficult to express recombinantly and this structure provides an elegant structural explanation for the role on SAP30BP in stabilising the cyclin L fold and promoting a catalytically active CDK11 conformation. The interaction of SAP30BP with cyclin L offers an exciting first example of how an extended unstructured protein interacts with a cyclin. Although structures of other transcriptional CDK-cyclin complexes have been determined, this example is the first of such an extended interaction interface and will be greeted with great interest by those interested in the control of gene transcription, protein regulation and small molecule inhibitor design. The findings are important and will implications for the mechanisms by which other transcriptional regulators might affect transcriptional CDK function. The determination of the structure of CDK11-cyclin L-SAP30BP bound to the potent CDK11 inhibitor OTS964 provides the first example of CDK11-cyclin L bound to an ATP-competitive inhibitor and together with the structural comparison of the inhibitor bound to monomeric CDK11 is an informative structure for further inhibitor design. The inclusion of structures for OTS964 bound to CDK7 and CDK2 digress somewhat from the paper's title but are well determined and increase the number of cryo-EM determined CDK-cyclin- ATP competitive inhibitor complexes which are still small in number.

Listed below are a number of major and minor comments that the authors might want to address in a revised manuscript:

Major comments
1. The interaction between cyclin L and SAP30BP
Page 5. The interaction between cyclin L and SAP30BP is extensive, and the structure reveals an unusual circling of the

cyclin by SAP30BP. However, there is very little description of the set of interactions between SAP30BP and cyclin L that are proposed to assist cyclin L stabilisation. CDK substrates and regulators employ a wide variety of Short Linear Motifs (SLiMs) to bind to the CDK-cyclin module. The manuscript would be improved if the section were to be expanded to include a more detailed analysis of the interactions, with accompanying figures to illustrate. Are SLiM sequences/ docking sequences driving the SAP30BP interaction indicated? It would be helpful to have a supplementary figure compiling the cyclin L, K and T sequence conservation and the cyclin L residues that interact with SAP30BP to enable comparative analysis.

2. Pull down experiments to demonstrate an interaction between cyclin L and SABP30BP
Figure 2g: In vitro pulldown of cyclin L2 in complex with SAP30BP (WT and 4A mutant. The control lanes showing sample input need to be included. At the moment, there is no evidence to show that the SAP30P4A mutant has been successfully expressed.

3. Structural evidence for a pseudo substrate mechanism of CDK11 regulation.
The most convincing evidence that CDK11 can accommodate a pseudo-substrate sequence in the active site is provided by the supporting electron density in the cryo-EM maps. The implications of this finding for CDK11 regulation will be of great interest to the field. It would be the first example of a CDK being regulated by this mechanism.

However, the manuscript extends the interpretation of this observation to propose a model in which CDK11 activity is (i) regulated by pseudo-substrate autoinhibition that is (ii) additionally subject to regulation by reversible S752 phosphorylation. The manuscript should include more substantial further experimentation to support this mechanistic model. This evidence should include both complementary orthogonal and more quantitative approaches to support it. The evidence currently provided is broadly supportive of the model but not definitive. Comments and points that should be addressed are:

(i) It would be helpful to understand the structural context of this pseudo-substrate sequence (text and figure). Can sufficient sequence be built N- and C-terminal to the motif to further support the sequence register within the active site? Could the text be expanded to include a description of how it emerges at the N-terminus from the CDK fold and how the sequence post the pseudo-substrate motif engages with the CDK domain.

(ii) The current figures (3c and 3d) have phosphorylated S752 sat behind the peptide backbone pointing away from the viewer. Please include views that more clearly illustrate the features of the pocket into which S752 points and elaborate the interactions made.

(iii) Within the proposed pseudo-substrate sequence, please indicate which residues occupy the P-3 to P+3 positions (where P is the site of phosphotransfer). (It would be helpful to understand the sequence register of the proposed CDK11 pseudosubstrate sequence aligned to that of CDK2). Please label on Figure panels 3c and d. The CDK2-cyclin A-peptide structure (3QHR, Bao et al), has peptide substrate sequence PKTPKKAKKL with the T residue in the P position. Please clarify the statement "The phosphate is accommodated in a pocket near the CDK11 active site (Fig. 3c, d)." Does the CDK11 phosphorylated S752 residue occupy the P position, and where is this pocket in relation to the CDK11 catalytic Asp? How does this affect the way in which the visualized peptide is a substrate peptide mimetic? S752 is partially phosphorylated in the sample and the structure was determined bound to AMP-PNP. Please elaborate how the S752 phosphate is accommodated in the presence of a non-hydrolysable ATP analog. The current description of the peptide binding mode suggests that it is not quite equivalent in sequence and structural features to pseudosubstrate binding as exemplified previously by peptides bound to CDK2 or PKA.

4. Biochemical evidence for a pseudosubstrate mechanism of CDK11 regulation
On page 7 it states that CHK2-dependent CDK11 S752 phosphorylation enhances splicing in a cell-based assay. The paper by Choi et al cited in support of this statement (Ref 36) reported that mutation of CDK11 S752 to Ala did not affect kinase activity, but abrogated CDK11 homodimerisation and its splicing-activating activity. They suggested that S752 phosphorylation was required for homodimerisation.

The prediction from the model proposed in this paper is that S752 phosphorylation would inhibit CDK11 catalytic activity by promoting a more stabilised pseudo-substrate bound conformation. This model would thus appear to contradict the results reported in Choi et al where S752 phosphorylation was proposed to be required for CDK11 homodimerisation and splicing activity.

Though these separate experiments might be reporting on different mechanisms that regulate CDK11 activity and could be reconciled, it will require substantial further work to deconvolute. Thus, there may be multiple mechanisms by which CDK11 S752 phosphorylation status may regulate CDK11 activity. The molecular mechanism might not be straightforward, and any revised model has to consider these prior findings.

(i) Kinase assay results reported in Figure 3f and g. The following points should be addressed:
(i.i) The fold changes are small and though statistically significant, the assay needs to be challenged by performing it with at least two (preferably 3) biological replicates over a more sampled time range. However, currently the assay results do not present a consistent story between timepoints, and don't include a sufficient kinetic range. Western blotting as the sole technique used to support this proposed mechanistic model is not sufficiently convincing. The results of using mutants to emulate phospho-amino acid properties should be treated with caution.
(i.ii) Characterisation of CDK11 activity should be carried out using an alternative quantitative assay format that can provide kinetic characterisation. Inhibition studies with OTS964 were performed by Reaction Biology (though noting CDK11 was

bound to cyclin K). This information suggests that CDK11 is amenable to assaying in the ADPGloTM format to permit quantitative kinetic characterisation. The manuscript argues that cyclin L association with SAP30BP stabilises the CDK11-cyclin L complex providing an opportunity to more fully characterise its WT activity and the activity of the proposed mutants. For example, use of this assay would enable characterisation of the expected effects of +/- pseudosubstrate sequence on Km measurements which should be included in support of the model.

Given the above considerations, further experimental evidence needs to be provided to support the inclusion of panel 3h in a Results figure.

(v) Enzyme inhibition assays, CDK activities and comparison to structures.
Please consider commenting on the following:
(v.i) CDK2 is not phosphorylated on the activation loop for structure determination but presumably is in the kinase assay outsourced to Reaction Biology.
(v.ii) CDK11 is assayed bound to cyclin K (a non-cognate cyclin), but the structure is determined bound to cyclin L. Please consider commenting on whether structural inferences of inhibitor binding to CDK11 bound to cyclin L reflect the activity measured bound to cyclin K.

Minor comments
1. Paragraph 2 page 4. Please state which residue ranges are modelled in the final structure for each protein.

2. Please add the expressed construct residue ranges to Supplementary Figure 1A.

3. Figure 1. This Figure would have more impact if it only displayed structures, and it should provide experimental evidence of map quality in the main manuscript. Consider to include (i) exemplar local electron density with modelled structure (consider moving Figure S2e-f) and (ii) overall cryo EM map coloured to identify proteins (appropriately labelled). Consider keeping the view in the same orientation as Figure S2B for ease of comparison. Would suggest that the SDS-PAGE purification be moved to Figure S1. To orient the view, consider displaying CDK11-cyclin L in the "canonical" kinase fold view. Then indicate the rotations required to generate the other views that best illustrate the SAP30BP binding mode.

4. Figure labelling: Figure 1C: Label cyclin N-CBF and C-CBF. Label selected α-helices and ID all α-helices on SAP30BP. Label all atoms/molecules drawn as spheres (green spheres, additional phosphate cluster in RHS panel).

5. For local resolution analysis in Figure S2b- Label key features. Would suggest that this figure panel is in the same view as Figure 1C.
Statement p5: " SAP30BP residues 98-184 form six α-helical segments connected by extended linkers that encircle the C-terminal cyclin fold of cyclin L." Figure 1C rather shows it draping around and over both cyclin L CBFs. Please comment further.

6. To clarify the experimental evidence in support of the pseudosubstrate inhibition model, could the following statement be re-written to distinguish the experimental data (included in Fig. 3c) from the AlphaFold3 modelling.

"Features of the observed density agree with the identity of CDK11 residues 751-762 (TSPRPPEGGLGF) predicted to occupy this site by AlphaFold3 30 (Fig. 3c).

Features of the observed density agree with the identity of CDK11 residues 751-762 (TSPRPPEGGLGF) (Fig. 3c). This sequence is predicted to occupy this site by AlphaFold330 (Supplementary Figure 3).

7. Supplementary Figure 3 does not illustrate the AlphaFold3 model in the vicinity of the active site in support of a CDK11 sequence bound within the active site cleft. Please include a figure to support the statement above.

8. Figure 3C, expanded panel. Please include labelling to ID CDK11 sequence features and the proposed pseudo-substrate sequence register.

6. Figure 4g. Can the colour contrast between CDK8 and MED12 be heightened. Though the N- and C-CBFs are coloured in distinct shades, please label. The views in panels (a), (d) and (g) look similar, also are the views in panels (c), (f), (i) and (l) comparable? Could the figure legend confirm the structures have been superimposed and the views in each cluster of figure panels are the same.

7. The assay format used by Reaction Biology (radiometric or ADP-GloTM) should be included in the methods.

8. Please include a description in the Cryo-EM sample preparation as to why a 21-mer cryo-peptide was included.

Version 1:

Reviewer comments:

Reviewer #1

(Remarks to the Author)
The reviewers have sufficiently addressed my comments and I support publication in Nat. Com.

Reviewer #2

(Remarks to the Author)
The authors have fully addressed my concerns and questions and appropriately revised the manuscript and figures. I have no further comments.

Reviewer #3

(Remarks to the Author)
I would like to thank the authors for addressing my comments with such care. I have no further comments to make.

Open Access This Peer Review File is licensed under a Creative Commons Attribution 4.0 International License, which permits use, sharing, adaptation, distribution and reproduction in any medium or format, as long as you give appropriate credit to the original author(s) and the source, provide a link to the Creative Commons license, and indicate if changes were made.
In cases where reviewers are anonymous, credit should be given to 'Anonymous Referee' and the source.
The images or other third party material in this Peer Review File are included in the article’s Creative Commons license, unless indicated otherwise in a credit line to the material. If material is not included in the article’s Creative Commons license and your intended use is not permitted by statutory regulation or exceeds the permitted use, you will need to obtain permission directly from the copyright holder.
To view a copy of this license, visit https://creativecommons.org/licenses/by/4.0/

Answers to reviewers: McGeoch et al., *Nature Communications*, 2026

Reviewer #1 (Remarks to the Author):

In this paper McGeoch and colleague investigate the molecular mechanism of CDK11 activation using biochemical and structural methods. To this end the authors in vitro reconstitute the CDK11-cyclin L-SAP30BP complex and determine the structure of this complex to high-resolution using cryoEM. In addition the authors determine the structure of this complex bound to the inhibitor OTS964 and compare this structure to a previous crystal structure of this inhibitor bound to the isolated kinase domain of CDK11. Overall this paper is of excellent quality and I enjoyed reading it. The paper is very clearly written, and also the figures are intuitive and informative. The authors did a great job in conveying their findings. In my opinion this paper is of such high quality that justifies a publication in Nature Communications.

We thank the reviewer for their positive assessment of our work and their insightful suggestions for improvement of our manuscript.

I have only a few minor comments to make:

1) "To facilitate structure determination, we truncated the N-terminus of CDK11, generating CDK11p58 (CDK11Bp110 residues 357-795) and removed the C-terminal tail of cyclin L2, including its predicted disordered RS- domain, resulting in expression of cyclin L2 residues 1-319 (Supplementary Fig. 1a, b)."

This description seems vague. Are the authors implying that the unstructured N-terminus of CDK1 and the C-terminal tail of cyclin L2 would preclude particle alignment by contributing to noise or is it that the truncation resulted in more stable and better expressed complexes? I would suggest to modify this sentence.

In our experience, long, unstructured regions can adversely affect the particle distribution and their orientation on the cryo-EM grids. Additionally, we observed that $CDK11^{p110}$-containing preparations tended to be less pure than $CDK11^{p58}$-containing samples. Therefore, taken together, the truncations therefore facilitated high-resolution structure determination.

We changed this sentence to: "To facilitate purification of homogeneous protein complexes and cryo-EM grid preparation, we truncated the N-terminus of CDK11, generating CDK11p58 (CDK11Bp110 residues 357-795) and removed the C-terminal tail of cyclin L2, including its predicted disordered RS-domain, resulting in expression of cyclin L2 residues 1-319 (Supplementary Fig. 1a, b)."

2) "The T-loop of CDK11 is found in an extended, active conformation, and the features of our cryo-EM map indicate that it is phosphorylated at T595 (CDK11Bp110 residue numbering; Fig. 1c, Supplementary Fig. 2g)."

The discovery that CDK-cyclin complexes are activated when co-expressed in insect

cells has been described before (Yu et al., 2021) and (Pim J Huis in't Veld, 2022). Since a similar strategy has been applied for this work it might be appropriate to cite such papers.

We agree with the reviewer that this is a previously documented strategy, and that this observation might have been expected, considering the prior evidence. We note that the work by Huis in’t Veld primarily used in-vitro phosphorylation or co-expression of human or yeast CDK-activating kinase activities (because phosphorylation in insect cells was low otherwise), which is not the case in our study.

Therefore, we have now cited work by Yu et al., *Nature*, 2021 (as suggested) and Duster et al., *Nat. Commun.*, 2024, along with our own paper on the CDK-activating kinase (which predated these papers and showed a S164-phosphorylated CDK7 T-loop).

A statement has been added: “The presence of post-translational modifications of the T-loop is in line with preparations of other CDKs from insect cells (refs. 32-34).”

3) “These interactions appear to be highly specific to cyclin L, as SAP30BP did not form stable complexes when co-expressed with the phylogenetically related cyclins H, K, and T (Fig. 2e) 32.”

A sequence alignment between the different cyclins L, H, K and T with residues highlighted that are involved in mediating interaction to SAP30BP might support this claim.

We are grateful for this suggestion and have added a multiple sequence alignment as suggested. This is now presented in Supplementary Fig. 5a. We have additionally added a structural representation of cyclin surface conservation relative to the sites of SAP30BP binding, as suggested by another reviewer. This is presented in Supplementary Fig. 5b.

In my opinion it is possible to observe a weak interaction with cyclin T in the provided gel and I would therefore also slightly rephrase this sentence (e.g. only weak/transient interactions are formed). But this is only meant as a suggestion.

We agree with the reviewer that a very weak band in the amylose pulldown for cyclin T is visible in Fig. 2e (though we note that we do not observe a corresponding enhancement of the SAP30BP band in the cyclin T pull-down in the left-hand panel). Overall, we believe that the suggested qualifier regarding interaction strength is already contained in the term “stable” complexes in sentence as currently stated, so we prefer to leave the sentence as-is. To clarify further, we have added an additional statement to the figure legend:

“Only His_6-cyclin L2 co-purifies MBP-SAP30BP (nickel-sepharose elution), and MBP-SAP30BP co-purifies His_6-cyclin L2 (amylose elution; *a weak band in the cyclin T elution denoted by an asterisk might correspond to a weakly bound or transiently forming complex)*.”

4) “We note that substantial kinase activity is observed in all CDK11 variants tested, indicating that none of the tested configurations of S752 results in complete inhibition, probably because true substrates can outcompete all configurations of the pseudo-substrate, albeit with variable efficiency”

Affinity measurements with several peptides (phosphorylated and non-phosphorylated) that resemble the C-terminal psuedo-substrate tail and CDK11 substrates, e.g. SF3B1 might shed further light on the inhibitory mechanism of the C-terminal tail.

We appreciate that a full characterisation of the properties of the putative pseudo-substrate within CDK11 will further illuminate the associate regulatory mechanisms. However, in the light of certain technical challenges we faced during the revision (see also responses to reviewer 3, below), we believe that this full characterisation exceeds the scope of the present manuscript and will need to be conducted as a dedicated, independent study.

5) “..., results reported in that study indicate that cyclin binding improves OTS964 affinity for CDKs and suggest that the isolated kinase domain might not be able to fully recapitulate the compound-binding properties of the full-length cellular CDK11 protein 29“.

Since Kelso et al. did not perform affinity measurements using a CDK11-cyclin L complex (due to expression difficulties – which are nicely summarised in this study), it would be interesting to compare binding affinities of OTS964 bound to insolated CDK11 with binding of OTS964 to the CDK11-cyclin L-SAP30BP complex.

We agree that testing this hypothesis was important. We have therefore performed these ITC experiments and found that the affinity difference between CDK11 monomer and CDK11-cyclin L-SAP30BP complex is marginal (approx. two-fold). The data are now shown in Supplementary Fig. 8.

In brief, this is a very well designed and executed study with interesting findings that warrant publication in Nature Communications. The paper is clearly written and the figures are of the same high quality. Congratulations to the authors. Nevertheless, I think the manuscript would benefit from adding some affinity measurements and small text modifications.

We appreciated the positive overall assessment of our study and have made a number of modifications as suggested.

Reviewer #2 (Remarks to the Author):

The study by McGeoch and co-workers presents the high-resolution (2.3 Å) cryo-EM structure of the human CDK11-cyclin L2-SAP30BP complex, which is crucial for the regulation of the spliceosome, e.g. through the phosphorylation of SF3B1. The structure shows that SAP30BP stabilizes cyclin L2 and promotes CDK11 complex assembly by

forming extensive interactions with cyclin L2 and modest interactions with the CDK11 T-loop. A pseudo-substrate sequence motif about 30 residues C-terminal to the kinase domain was identified, acting as an auto-regulatory element modulated by phosphorylation. Additionally, the authors determined the structure of the CDK11 complex bound to the clinical inhibitor OTS964, providing insights into its specificity and mechanism of action. Structural comparisons with off-target CDKs CDK2 and CDK7 explain OTS964's selectivity for CDK11. Overall, the study provides a comprehensive framework for understanding CDK11 structure and regulation, and offers strategies for targeted therapeutic development targeting this transcriptional kinase.

This is a very timely and important study on one of the missing pieces in transcriptional kinase regulation. I appreciate the high standard of structural characterization and the comprehensive analysis of compound binding. I have only a couple of comments and suggestion to this study.

We thank the reviewer for their evaluation of our study and the helpful suggestions provided.

Comments:
Introduction, page 3. Please write out the names for kinases PBK (TOPK).

This has been added: "… mischaracterised as a PDZ-binding kinase (PBK)/Lymphokine-activated killer T-cell originated protein kinase (TOPK) inhibitor."

Results and Discussion, page 6, bottom: Does the magnesium coordination involve any of the pseudo-substrate residues? Although the pseudo-substrate motif is not introduced until the following paragraph, it would be nice if the authors could make a preliminary statement as to whether the density of the second magnesium is only due to the presence of the pseudo-substrate density.

We agree that this is an important question. However, the pseudo-substrate segment is not involved in magnesium coordination. The phosphate group on S752 and any other suitably polarised groups within the pseudo-substrate are too far away from the magnesium to interact with it. We have added a panel that clarifies this question (also in response to similar comments made by reviewer 3). This is now presented in Fig. 3d, f, g.

Page 7: The G-rich loop of CDK11 reads GTYG (but not GTYGG) and encompasses residues 447-450. More common would be the denomination as EEGTYG (445-450), where CDK11 is one of the few exceptions where the first glycine from the consensus sequence GxGTYG is changed to glutamate.

We very much thank the reviewer for spotting this mistake and have corrected this sentence:

"One exception to this is the G-rich loop of CDK11 (residues 445-450, sequence EEGTYG), which exhibits weak density, indicating flexibility."

Page 9: To this reviewers' knowledge, the terminology for cyclins is that they are composed of two cyclin boxes (usually with high structural homology), which are designated as first and second cyclin box, but not N- and C-terminal cyclin domain.

We thank the reviewer for pointing out this inaccuracy. We have now adopted the terminology of Noble, Endicott, et al. (TIBS 1997), who use N- and C-terminal "cyclin box fold", which is also in line with the terminology used by reviewer 3. This nomenclature is now used throughout the manuscript and figures.

Figure 5: The kinases in panels e and f should be labelled as CDK7 and CDK2, similar to CDK11 in panels b and c.

We thank the reviewer for pointing this out. This has been corrected.

Figure 6: I consider the CDK11 dimerization model in panel b highly unlikely, but this is also mentioned in the text. Any chances, that the linker region between the C-term of the kinase domain and the pseudo-substrate motif pS752 (in cis-assembly) could be resolved in the density map upon different filtering/masking?

The density is not fully continuous, but limited b-factor sharpening and low-pass filtering the OTS964-containing map to 3.3 Å resolution reveals additional density that stretches towards the last built residue on the N-terminal side of the gap between the C-terminal kinase lobe of CDK11 and the pseudosubstrate segment. This observation supports the conclusion that this segment is connected to CDK11, but the density is not of sufficient quality to reliably build a continuous model connected to the C-terminus of the CDK11 C-terminal kinase lobe.

A rendering of this density is now presented in Supplementary Fig. 9j, and this is mentioned in the text:

"Indeed, applying limited b-factor sharpening of -10 $Å^2$ and low-pass filtering the cryo-EM density of the OTS964-bound complex to 3.3 Å resolution shows further density extending from the pseudo-substrate segment towards its connection to the C-terminal kinase lobe (Supplementary Fig. 9j). Even though the connecting density is too weak to build a reliable model into it, this observation supports the assignment of the density occupying the CDK11 substrate binding site to a sequence element of the kinase itself."

Supplementary Fig. 1: The pseudo-substrate motif at position 752 should be indicated in the bar diagram of panel a.

This information has now been added as suggested.

Supplementary Fig. 7: I suppose, it should read CDK11B-cyclin L in panel a.

CDK11B-cyclin K is accurate (now Supplementary Fig. 11). This experiment was

performed by Reaction Biology using an in-house enzyme (see also the response to a comment from reviewer 3 and the updated figure legend for more detail).

Reviewer #3 (Remarks to the Author):

In this manuscript McGeoch et al. present the structure of the CDK11-cyclinL-SAP30BP complex. CDK11-cyclin L has proved difficult to express recombinantly and this structure provides an elegant structural explanation for the role on SAP30BP in stabilising the cyclin L fold and promoting a catalytically active CDK11 conformation. The interaction of SAP30BP with cyclin L offers an exciting first example of how an extended unstructured protein interacts with a cyclin. Although structures of other transcriptional CDK-cyclin complexes have been determined, this example is the first of such an extended interaction interface and will be greeted with great interest by those interested in the control of gene transcription, protein regulation and small molecule inhibitor design. The findings are important and will implications for the mechanisms by which other transcriptional regulators might affect transcriptional CDK function. The determination of the structure of CDK11-cyclin L-SAP30BP bound to the potent CDK11 inhibitor OTS964 provides the first example of CDK11-cyclin L bound to an ATP-competitive inhibitor and together with the structural comparison of the inhibitor bound to monomeric CDK11 is an informative structure for further inhibitor design.

We thank the reviewer for their careful assessment of our manuscript and their insightful comments and suggestions.

The inclusion of structures for OTS964 bound to CDK7 and CDK2 digress somewhat from the paper's title but are well determined and increase the number of cryo-EM determined CDK-cyclin- ATP competitive inhibitor complexes which are still small in number.

While we do believe that the CDK2- and CDK7-bound inhibitor structures are relevant in the wider context of our work on CDK11, we agree that they are not as central to the paper’s conclusions as the remaining parts are. We have therefore shortened the discussion of these two structures in the main text and provide the full discussion as Supplementary Notes 1 and 2 instead.

Listed below are a number of major and minor comments that the authors might want to address in a revised manuscript:

Major comments:

1. The interaction between cyclin L and SAP30BP

Page 5. The interaction between cyclin L and SAP30BP is extensive, and the structure reveals an unusual circling of the cyclin by SAP30BP. However, there is very little description of the set of interactions between SAP30BP and cyclin L that are proposed to assist cyclin L stabilisation. CDK substrates and regulators employ a wide variety of Short Linear Motifs (SLiMs) to bind to the CDK-cyclin module. The manuscript would be improved if the section were to be expanded to include a more detailed analysis of the

interactions, with accompanying figures to illustrate. Are SLiM sequences/ docking sequences driving the SAP30BP interaction indicated?

We are grateful for this request for further information on the unusual interaction between SAP30BP and cyclin L2. There are two extended stretches of SAP30BP that form meaningful interactions with cyclin L2. Neither of these is using the known hydrophobic groove region on the cyclin, and considering the extensive interfaces contributed by the docked alpha-helices, concluding that these segments drive the interaction would not be warranted. Additionally, SLiMs often contain conserved motifs that are found in multiple interactors (e.g. RxL/Cy motifs interacting with cyclins), and we do not currently possess evidence that this is the case here. Overall, the extended segments of SAP30BP are certainly contributing to the interaction with cyclin L, but we do not think that we should refer to them as SLiMs. To illustrate our observations, we have provided additional figure panels, which are presented in Supplementary Fig. 4.

It would be helpful to have a supplementary figure compiling the cyclin L, K and T sequence conservation and the cyclin L residues that interact with SAP30BP to enable comparative analysis.

We appreciate this suggestion and we have now included this information in Supplementary Fig. 5.

2. Pull down experiments to demonstrate an interaction between cyclin L and SABP30BP Figure 2g: In vitro pulldown of cyclin L2 in complex with SAP30BP (WT and 4A mutant. The control lanes showing sample input need to be included. At the moment, there is no evidence to show that the SAP30P4A mutant has been successfully expressed.

The reviewer is correct. This information has now been added Fig. 2g. We apologise for this omission.

3. Structural evidence for a pseudo substrate mechanism of CDK11 regulation. The most convincing evidence that CDK11 can accommodate a pseudo-substrate sequence in the active site is provided by the supporting electron density in the cryo-EM maps. The implications of this finding for CDK11 regulation will be of great interest to the field. It would be the first example of a CDK being regulated by this mechanism.

We appreciate the reviewer's comments on this topic, which have guided us in improving this aspect of the manuscript. While we will address the specific points below, it will be helpful to highlight from the outset that we have re-structured the treatment of this finding in order to more clearly separate our direct evidentiary findings from more speculative mechanistic interpretations. The latter are now contained in a separate Discussion section, and the figure panels relating to the discussion have been removed from the results figures (both from main figures and supplementary figures) and instead have been collected in a supplementary figure that is only referenced from within the discussion itself. We appreciate that given the complexity of the system and the multiple substrates and pathways impacted by CDK11, conclusive support for (or refutation of) some of the detailed mechanistic implications of our findings will require substantial

amounts of additional work that will exceed the scope of our current manuscript. At the same time, we believe that the extended structural and biochemical analysis provided with this revision support the conclusions drawn in the current version of our manuscript.

However, the manuscript extends the interpretation of this observation to propose a model in which CDK11 activity is (i) regulated by pseudo-substrate autoinhibition that is (ii) additionally subject to regulation by reversible S752 phosphorylation. The manuscript should include more substantial further experimentation to support this mechanistic model. This evidence should include both complementary orthogonal and more quantitative approaches to support it. The evidence currently provided is broadly supportive of the model but not definitive.

We appreciate these observations and, as outlined above, have more clearly segregated our results from the discussion of possible mechanistic implications. In addition, we have conducted additional experimental work, as outlined below.

Comments and points that should be addressed are:

(i) It would be helpful to understand the structural context of this pseudo-substrate sequence (text and figure). Can sufficient sequence be built N- and C-terminal to the motif to further support the sequence register within the active site? Could the text be expanded to include a description of how it emerges at the N-terminus from the CDK fold and how the sequence post the pseudo-substrate motif engages with the CDK domain.

The density from the core CDK fold to the part of the pseudo-substrate visualised in the cryo-EM map is not continuous. However, limited b-factor sharpening and low-pass filtering of the OTS964-containing complex shows additional density that is consistent with our interpretation (even though it is not detailed enough to reliably build an atomic model into it). A rendering of this putative connecting density is now shown in Supplementary Fig. 9j.

(ii) The current figures (3c and 3d) have phosphorylated S752 sat behind the peptide backbone pointing away from the viewer. Please include views that more clearly illustrate the features of the pocket into which S752 points and elaborate the interactions made.

We agree that this was not ideal and thank the reviewer for highlighting the issue. We have now provided additional views (i) with the density (Fig. 3d), (ii) in the context of the atomic model and (iii) visualising the positioning of S752 relative to the P_{-3} to P_{+3} positions observed in true substrates bound to CDKs (Fig. 3g).

(iii) Within the proposed pseudo-substrate sequence, please indicate which residues occupy the P-3 to P+3 positions (where P is the site of phosphotransfer). (It would be helpful to understand the sequence register of the proposed CDK11 pseudosubstrate sequence aligned to that of CDK2). Please label on Figure panels 3c and d. The CDK2-cyclin A-peptide structure (3QHR, Bao et al), has peptide substrate sequence PKTPKKAKKL with the T residue in the P position.

We have provided additional figure panels (Fig. 3g, i) that provide detailed information on the positioning of S752 relative to the positioning of true substrates in CDK active sites.

We have also added further information to the text:

"The position of the phosphorylatable residue in actual substrates (denoted P_0) is occupied by P755 in active site-occluding peptide in CDK11, precluding catalysis from occurring. The neighbouring P_{+1}, P_{-1}, P_{-2}, and P_{-3} positions are occupied by P756, R754, P753, and the phosphorylatable S752 (Fig. 3f, g). Proline in the P_{+1} position satisfies the canonical sequence preference of CDKs, which are proline-directed [35,44-46]. Indeed, the configuration of the CDK11 active site-occluding peptide with prolines in the P_{+1} and P_{-2} positions and a positively charged residue in the P_{-1} position closely resembles the structure of a CDK2 complex bound to a synthetic peptide (Fig. 3f-i) [41]. This indicates that the CDK11 segment occupying its own substrate-binding site contains residues mimicking the properties of true CDK substrates in several positions in the immediate vicinity of the P_0 acceptor site. In positions P_{+4} and P_{+6} at the C-terminal side of the P_0 position, two glycine residues (G759 and G761) are positioned such that their amide protons can form interactions with the phosphorylated side chain of T595 within the CDK11 T-loop (Supplementary Fig. 6a, b). These interactions appear to replace the salt bridge observed between the T-loop phosphate and the positively charged P_{+3} residue in substrates bearing the full (S/T)-P-X-(R/K) consensus sequence (Supplementary Fig. 6c) [35,41]."

Please clarify the statement "The phosphate is accommodated in a pocket near the CDK11 active site (Fig. 3c, d)." Does the CDK11 phosphorylated S752 residue occupy the P position, and where is this pocket in relation to the CDK11 catalytic Asp?

The phosphorylated S752 occupies the P_{-3} position. The P_0 position is occupied by a proline, thereby blocking phosphorylation of the pseudo-substrate. The S752-phosphate is not in close proximity to the AMP-PNP phosphates, the catalytic aspartate, or the bound magnesium ions. This has been clarified in Fig. 3d, g and in the text:

"The phosphate is accommodated in a pocket near the CDK11 active site, forming interactions with the side chains of CDK11 K524, K564, and S566 (Fig. 3c, d). This pocket is distinct from the active site pocket itself, and the phosphorylated S752 does not form direct interactions with the bound AMP-PNP nucleotide, the catalytic aspartate ($CDK11^{p110}$ D580), or the bound magnesium ions (Fig. 3f, g)."

How does this affect the way in which the visualized peptide is a substrate peptide mimetic? S752 is partially phosphorylated in the sample and the structure was determined bound to AMP-PNP. Please elaborate how the S752 phosphate is accommodated in the presence of a non-hydrolysable ATP analog.

We apologise for leaving this ambiguous in our initial analysis. The S752-phosphate and the γ-phosphate of AMPPNP do not compete for the same binding pocket (please see Fig. 3d, g for detailed views of the relative arrangement of these chemical groups).

The current description of the peptide binding mode suggests that it is not quite equivalent in sequence and structural features to pseudosubstrate binding as exemplified previously by peptides bound to CDK2 or PKA.

We appreciate the reviewer's concern and hope that the additional information provided with this revision (as detailed in the responses to the comments above) clarifies the similarities and differences between our proposed CDK11 pseudo-substrate and previous structural and biochemical data.

4. Biochemical evidence for a pseudosubstrate mechanism of CDK11 regulation
On page 7 it states that CHK2-dependent CDK11 S752 phosphorylation enhances splicing in a cell-based assay. The paper by Choi et al cited in support of this statement (Ref 36) reported that mutation of CDK11 S752 to Ala did not affect kinase activity, but abrogated CDK11 homodimerisation and its splicing-activating activity. They suggested that S752 phosphorylation was required for homodimerisation.

The prediction from the model proposed in this paper is that S752 phosphorylation would inhibit CDK11 catalytic activity by promoting a more stabilised pseudo-substrate bound conformation. This model would thus appear to contradict the results reported in Choi et al where S752 phosphorylation was proposed to be required for CDK11 homodimerisation and splicing activity.

Though these separate experiments might be reporting on different mechanisms that regulate CDK11 activity and could be reconciled, it will require substantial further work to deconvolute. Thus, there may be multiple mechanisms by which CDK11 S752 phosphorylation status may regulate CDK11 activity. The molecular mechanism might not be straightforward, and any revised model has to consider these prior findings.

We agree with this assessment. We do not currently understand the mechanistic details of how our observations connect to the functional effects observed by Choi et al.; overall, our findings suggest that these regulatory mechanisms are more complex, and that further functional work is required to tease these apart. However, we also believe that this would extend beyond the scope of our current study. We have highlighted this apparent discrepancy in our Discussion in order to best inform the reader, and we propose two possible molecular mechanisms that could explain all observations (Supplementary Fig. 12b, c).

(i) Kinase assay results reported in Figure 3f and g. The following points should be addressed:
(i.i) The fold changes are small and though statistically significant, the assay needs to be challenged by performing it with at least two (preferably 3) biological replicates over a more sampled time range. However, currently the assay results do not present a consistent story between timepoints, and don't include a sufficient kinetic range. Western blotting as the sole technique used to support this proposed mechanistic model is not sufficiently convincing. The results of using mutants to emulate phospho-amino acid properties should be treated with caution.

We have now repeated this experiment with three biological replicates and with increased temporal samplings. The results are consistent with our prior experiments. These data are presented in Fig. 4.

While we note that phosphomimetic and non-phosphorylatable mutants are widely used to assess the effects of the phosphorylation status of phosphorylatable residues, we also agree that phosphomimetic mutations may not always be faithful mimics of the mechanistic effects of true phosphorylation. To avoid possible confounding effects, e.g. arising from variably phosphorylated S752 in WT CDK11, we have opted for separate panels for display of the data from CDK11 WT and a CDK11 C-terminal deletion mutant (ΔC), where the mechanistic implications are very clear, and the experiment using the S752A and S752E point mutations with well-defined charge states of residue 752. We note that the results match our predictions, with the phosphomimetic mutation (S752E) causing a reduction of kinase activity relative to the non-phosphorylatable mutation (S752A).

(i.ii) Characterisation of CDK11 activity should be carried out using an alternative quantitative assay format that can provide kinetic characterisation. Inhibition studies with OTS964 were performed by Reaction Biology (though noting CDK11 was bound to cyclin K). This information suggests that CDK11 is amenable to assaying in the ADPGloTM format to permit quantitative kinetic characterisation.

We note that the experiments at Reaction Biology used a radiometric kinase assay. However, we have attempted to incorporate ADP-Glo assays into our work (see below).

The manuscript argues that cyclin L association with SAP30BP stabilises the CDK11-cyclin L complex providing an opportunity to more fully characterise its WT activity and the activity of the proposed mutants. For example, use of this assay would enable characterisation of the expected effects of +/- pseudosubstrate sequence on Km measurements which should be included in support of the model.

Supported by the enzyme assay experts at the ICR Centre for Cancer Drug Discovery (CCDD), we have attempted to implement an ADPGlo assay for CDK11-cyclin L-SAP30. Unfortunately, these efforts were impeded by persistent experimental issues that were independent of the CDK11 variant, or the substrate type (recombinantly expressed or chemically synthesised peptide) used. Given that these assays are performed routinely at the CCDD, eliminating instrumentation or methodological issues as possible root causes, we concluded that these issues were specifically associated with our biological system.

Therefore, while we agree that a full kinetic characterisation of CDK11 and its pseudo-substrate mutants is important to unravel the mechanistic details of CDK11 regulation, particularly in the context of different types of substrates, we believe that our current data support the conclusions in our manuscript, and that a full characterisation needs to be the subject of a dedicated, separate study.

Given the above considerations, further experimental evidence needs to be provided to support the inclusion of panel 3h in a Results figure.

Panel 3h has been removed from this main text results figure and is now placed in a supplementary figure exclusively referred to from the Discussion section. The discussion itself clearly highlights which remaining questions will need to be addressed by further experimentation.

(v) Enzyme inhibition assays, CDK activities and comparison to structures. Please consider commenting on the following:

(v.i) CDK2 is not phosphorylated on the activation loop for structure determination but presumably is in the kinase assay outsourced to Reaction Biology.

We have compared the crystal structure of a CDK2-cyclin A complex with phosphorylated T-loop (Russo et al., *Nat. Struct. Mol. Biol.* **3**: 696-700, 1996; PDB 1JST) to our CDK2-cyclin A-OTS964 structure, and we conclude that the phosphorylation state of the T-loop does not affect the conformation of the residues in the vicinity of the inhibitor. There are differences between these two structures, particularly in the N-terminal kinase lobe, but these are likely connected to the binding of the inhibitor, not the phosphorylation state of the protein.

A comparison is now shown in Supplementary Fig. 11.

(v.ii) CDK11 is assayed bound to cyclin K (a non-cognate cyclin), but the structure is determined bound to cyclin L. Please consider commenting on whether structural inferences of inhibitor binding to CDK11 bound to cyclin L reflect the activity measured bound to cyclin K.

As referenced in the main text, the purpose of the experiment shown in Supplementary Fig. 11a, b was to establish the inhibitory properties of OTS964 against CDK7-cyclin H-MAT1 complexes, for which we could not find published inhibition data that were directly (numerically) comparable to the well-documented inhibitory properties of OTS964 against CDK11 complexes (e.g. Lin et al., *Sci. Transl. Med.* **11**: eaaw8412, 2019). In addition to the CDK7- and CDK2-containing off-target complexes, we included the CDK11 complex available at Reaction Biology as a benchmark. The results of this experiment revealed inhibitory properties that are highly consistent with the previous data on CDK11-cyclin L (Lin et al: K_D = 40 nM; our data IC_{50} = 41 nM). Our ITC data (Supplementary Fig. 8) are also generally consistent with this affinity range and show only marginal difference between CDK11 monomer and the cyclin-containing complex.

Our interpretations of the CDK11-OTS964 interaction therefore do not rely on the CDK11-cyclin K data from Reaction Biology, and we do not think that our interpretations were negatively impacted by the use of these values for the purpose of comparison to the much lower affinities of OTS964 to CDK7 and CDK2.

We have added a statement to the figure legend: “The values for CDK11B-cyclin K obtained are in agreement with literature data on endogenous CDK11B-cyclin L2 complexes (ref. 5) and in vitro data (ref. 4), thus confirming that OTS964 affinity for CDK2 and CDK7 is indeed much lower than for CDK11-containing complexes.”

Minor comments:

1. Paragraph 2 page 4. Please state which residue ranges are modelled in the final structure for each protein.

This has been added: CDK11B^{p110} residues 427-740 and 751-762; cyclin L2 residues 67-302; SAP30BP residues 98-215.

2. Please add the expressed construct residue ranges to Supplementary Figure 1A.

The constructs were expressed in the configurations shown in Supplementary Fig. 1a (i.e. the residue ranges given are already the expressed construct residue ranges). The constructs were combined into complexes as shown in Supplementary Fig. 1b.

3. Figure 1. This Figure would have more impact if it only displayed structures, and it should provide experimental evidence of map quality in the main manuscript. Consider to include (i) exemplar local electron density with modelled structure (consider moving Figure S2e-f) and (ii) overall cryo EM map coloured to identify proteins (appropriately labelled). Consider keeping the view in the same orientation as Figure S2B for ease of comparison. Would suggest that the SDS-PAGE purification be moved to Figure S1. To orient the view, consider displaying CDK11-cyclin L in the "canonical" kinase fold view. Then indicate the rotations required to generate the other views that best illustrate the SAP30BP binding mode.

We have applied these suggestions, and we have clarified in the figure legends that the views in Fig. 1b and c as well as Supplementary Fig. 2b are the same.

4. Figure labelling: Figure 1C: Label cyclin N-CBF and C-CBF. Label selected α-helices and ID all α-helices on SAP30BP. Label all atoms/molecules drawn as spheres (green spheres, additional phosphate cluster in RHS panel).

These suggestions have been implemented.

5. For local resolution analysis in Figure S2b- Label key features.

As suggested, we have labelled the protein subunits in this depiction.

Would suggest that this figure panel is in the same view as Figure 1C.

We agree with this suggestion, and we can confirm that this is the case.

Statement p5: " SAP30BP residues 98-184 form six α-helical segments connected by

extended linkers that encircle the C-terminal cyclin fold of cyclin L." Figure 1C rather shows it draping around and over both cyclin L CBFs. Please comment further.

We thank the reviewer for identifying that inaccuracies existed in this statement. The residue range in question (SAP30BP 98-184) only encompasses the first five helices, which do encircle the C-CBF (as visualised e.g. in Fig. 2a). We have added a figure reference to Fig. 2a to this statement and have corrected the number of helical segments. The statement, now accurate, reads:

“This ring around cyclin L is sealed by a salt bridge between SAP30BP residues R110 and D184 (Fig. 2a, c, d). Subsequent residues of SAP30BP interact with the N-terminal cyclin fold of cyclin L, terminating in a long, sixth, alpha-helix that extends into the solvent and likely continues beyond the last residue (K215) modelled in our structure (Fig. 1c, 2c, d).”

6. To clarify the experimental evidence in support of the pseudosubstrate inhibition model, could the following statement be re-written to distinguish the experimental data (included in Fig. 3c) from the AlphaFold3 modelling.

"Features of the observed density agree with the identity of CDK11 residues 751-762 (TSPRPPEGGLGF) predicted to occupy this site by AlphaFold3 30 (Fig. 3c).

Features of the observed density agree with the identity of CDK11 residues 751-762 (TSPRPPEGGLGF) (Fig. 3c). This sequence is predicted to occupy this site by AlphaFold330 (Supplementary Figure 3).

We appreciate the suggestion and have implemented this change as proposed. The panel showing the structure prediction and the cryo-EM density is now part of main text Fig. 3:

“Features of the observed density agree with the identity of CDK11 residues 751-762 (TSPRPPEGGLGF; Fig. 3c, d). This sequence is predicted to occupy this site by AlphaFold3 31 (Fig. 3e).”

7. Supplementary Figure 3 does not illustrate the AlphaFold3 model in the vicinity of the active site in support of a CDK11 sequence bound within the active site cleft. Please include a figure to support the statement above.

This has now been included as Fig. 3e.

8. Figure 3C, expanded panel. Please include labelling to ID CDK11 sequence features and the proposed pseudo-substrate sequence register.

A more detailed panel with residue numbers is now shown in Fig. 3d.

6. Figure 4g. Can the colour contrast between CDK8 and MED12 be heightened.

We agree that the colour choice was not ideal and have updated the figure with a new colour scheme that renders the distinction more clearly (now Fig. 5g, h, i).

Though the N- and C-CBFs are coloured in distinct shades, please label.

This has been added.

The views in panels (a), (d) and (g) look similar, also are the views in panels (c), (f), (i) and (l) comparable? Could the figure legend confirm the structures have been superimposed and the views in each cluster of figure panels are the same.

The structures have been superposed and the viewing direction for each column is the same. This information has been added to the figure legend:

"For visualisation, the complexes were superposed on the CDK subunit and each column in the figure shows all complexes from the same viewing direction."

7. The assay format used by Reaction Biology (radiometric or ADP-GloTM) should be included in the methods.

The kinase assay involved radiometric detection of ^{33}P incorporation. This information (along with further experimental detail) has been added to the corresponding section in the methods:

"The assays were performed as radiometric kinase assays, measuring incorporation of 33P from [gamma-33P]-ATP. ATP concentrations were set to the apparent ATP-Km of the respective enzyme. Reactions were run for 60 min at 30°C before quenching with 2% (v/v) H3PO4. Incorporation of 33Pi was determined using a microplate scintillation counter. Raw data were normalized using high controls (full enzyme activity without inhibitor) and low controls (unspecific binding of radioactivity in the absence of both enzyme and inhibitor) on the same plates."

8. Please include a description in the Cryo-EM sample preparation as to why a 21-mer cryo-peptide was included.

We have added the following statement to explain the use of the cryo-peptide:

"The cryo-peptide was included because the complex showed extreme preferred orientation when grids were prepared using conventional methods, precluding determination of an interpretable reconstruction."
